# Characterization of Cyclic *N*‐Acyliminium Ions by Infrared Ion Spectroscopy

**DOI:** 10.1002/chem.202104078

**Published:** 2021-12-29

**Authors:** Jona Merx, Kas J. Houthuijs, Hidde Elferink, Eva Witlox, Jasmin Mecinović, Jos Oomens, Jonathan Martens, Thomas J. Boltje, Floris P. J. T. Rutjes

**Affiliations:** ^1^ Institute for Molecules and Materials, Synthetic Organic Chemistry Radboud University Heyendaalseweg 135 6525 AJ Nijmegen The Netherlands; ^2^ Institute for Molecules and Materials, FELIX Laboratory Radboud University Heyendaalseweg 135 6525 AJ Nijmegen The Netherlands; ^3^ Department of Physics, Chemistry and Pharmacy University of Southern Denmark Campusvej 55 5230 Odense Denmark

**Keywords:** DFT calculations, heterocycles, ion spectroscopy, *N*-acyliminium ion, stereoselectivity

## Abstract

*N‐*Acyliminium ions are highly reactive intermediates that are important for creating CC‐bonds adjacent to nitrogen atoms. Here we report the characterization of cyclic *N*‐acyliminium ions in the gas phase, generated by collision induced dissociation tandem mass spectrometry followed by infrared ion spectroscopy using the FELIX infrared free electron laser. Comparison of DFT calculated spectra with the experimentally observed IR spectra provided valuable insights in the conformations of the *N*‐acyliminium ions.

## Graphical abstract

*N‐*Acyliminium ions (NAIs) are highly reactive intermediates that are important for the construction of CC‐bonds adjacent to nitrogen atoms. These species have been characterized for the first time in the gas phase by collision induced dissociation tandem mass spectrometry followed by infrared ion spectroscopy (IRIS), which has led to new structural information that can be invoked to explain the diastereoselectivity of reactions involving these cationic intermediates.

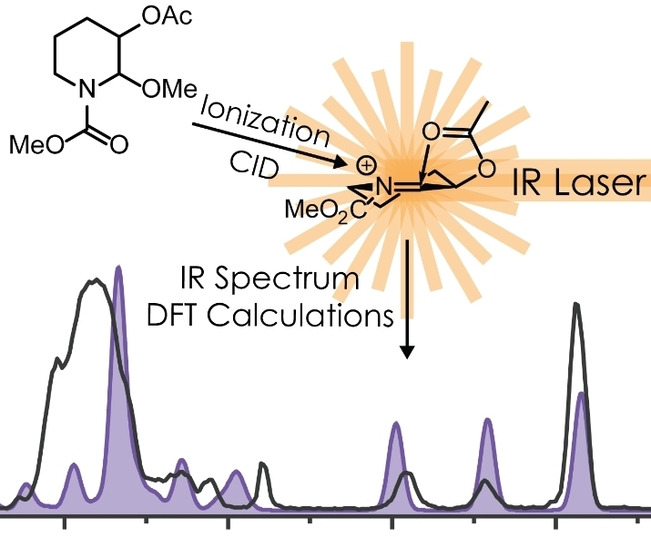

## Introduction

**Scheme 1:**
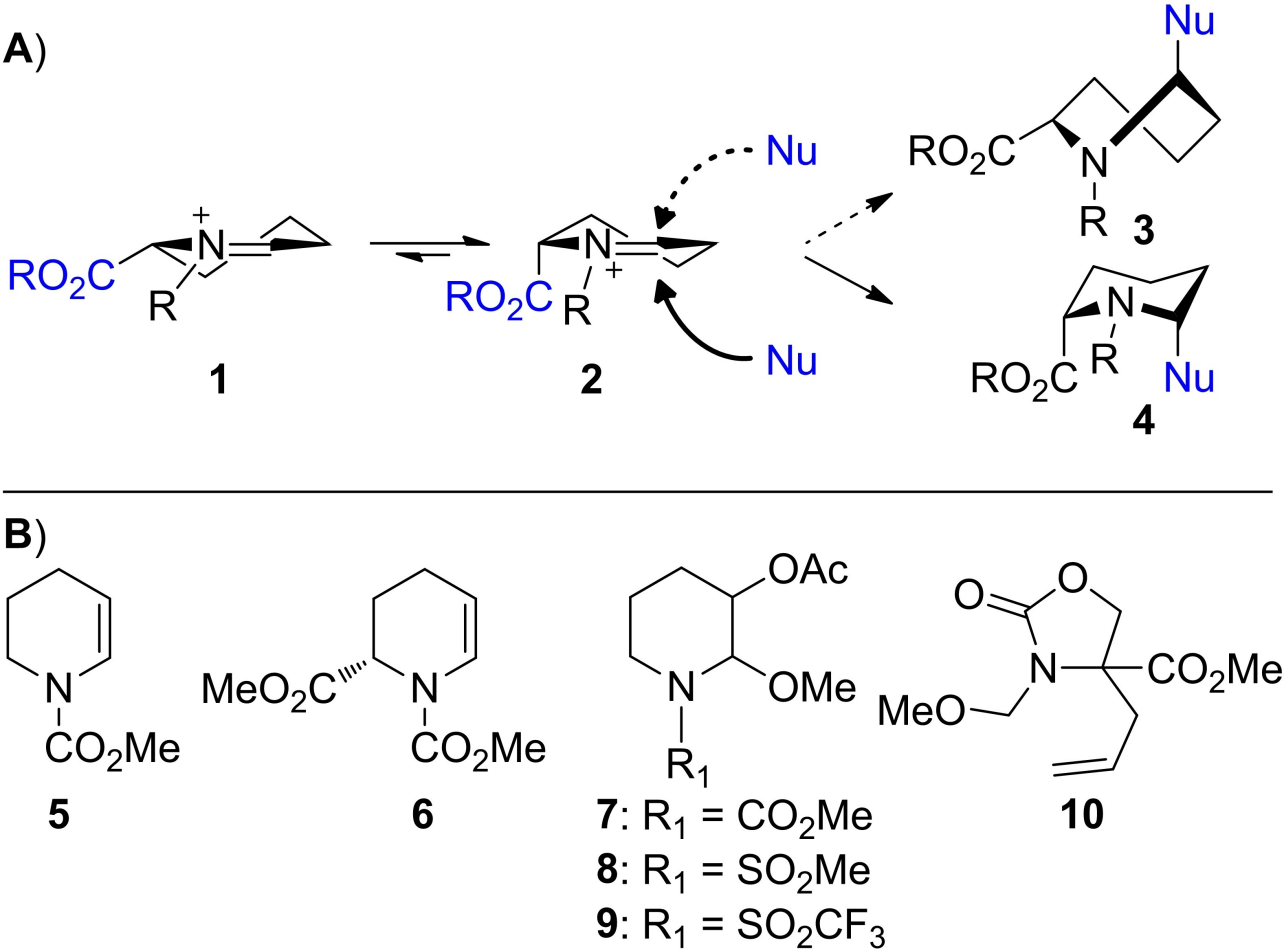
A) Cyclic *N*‐acyliminium ion conformations **1** and **2** and their preferential facial selectivity upon nucleophilic attack; Nu=nucleophile. B) NAI precursor molecules **5–10** used in this study.

One of the main challenges in organic synthesis is the stereoselective synthesis of carbon−carbon (CC) bonds. *N*‐Acyliminium ions (NAIs) are highly reactive electrophiles that have been extensively used for CC‐bond formation at the α‐position of amino groups in the synthesis of biologically relevant nitrogen heterocycles and alkaloid natural products.^[1]^ A useful class of NAIs in this respect is derived from *N*‐acylpiperidines. These precursors can be used to generate cyclic NAIs, which can react with π‐nucleophiles in a highly stereoselective manner. The conformation of a cyclic NAI and the orientation of the substituents determines the facial selectivity of reactions proceeding through this cationic intermediate.^[2]^ For example, 2‐substituted six‐membered ring NAIs exist of equilibrating half‐chair conformers **1** and **2**, of which the latter favors placement of the 2‐substituent in a pseudo‐axial orientation to minimize allylic A^1,3^‐strain between the ester and *N*‐acyl substituent (Scheme 1A).^[3]^ Nucleophilic addition to **2** is expected to proceed preferentially via pseudo‐axial attack directly resulting in chair‐like conformer **4**, whilst pseudo‐equatorial attack would lead to the less stable skew‐boat conformer **3** and is therefore disfavored.^[4]^ Furthermore, the reactivity and stereoselectivity of six‐membered ring NAIs has been modulated by the introduction of stereodirecting acyl groups, which stabilize the cationic center while simultaneously shielding one face from nucleophilic attack.^[5]^

Since the conformations of NAIs are crucial for the stereochemical outcome of CC‐bond formation, it is highly relevant to be able to structurally characterize these intermediates. Spectroscopic characterization of NAIs is, however, challenging due to their limited life‐time and reactive nature. NMR has been used to characterize relatively straightforward acyclic NAIs derived from *N*‐methylbenzylimine.^[6]^ More recently, low temperature NMR measurements combined with density functional theory (DFT) calculations allowed the study of simple alkyl‐ and aryl‐functionalized six‐membered ring *N*‐acyliminium ions.^[7]^ These NMR based approaches have remained limited to NAIs lacking substituents that could engage in neighboring group participation.

Here we report on the use of infrared ion spectroscopy (IRIS) to experimentally characterize NAI conformations and investigate how various participating groups contribute to their stabilization. To this end, electrospray ionization (ESI) was used to generate NAIs of interest in the gas phase, followed by isolation in an ion trap mass spectrometer and characterization via IR multiple‐photon dissociation (IRMPD) spectroscopy over a frequency range of 800–1880 cm^−1^.^[8]^ The IR spectra of these isolated ions provide structural insights, as demonstrated earlier by us^[9]^ and others^[10]^ for glycosylation intermediates. To assess the influence of substituents on the conformation of NAIs, we prepared and characterized a set of *N*‐acylpiperidines varying the *N*‐acyl, 3‐ and 6‐substituents (**5**–**9**, Scheme 1B). In addition, we characterized a rearrangement reaction product of NAI precursor **10** in the gas phase. To the best of our knowledge, IRIS has not previously been applied to the characterization of NAIs.

## Results and Discussion

**Figure 1:**
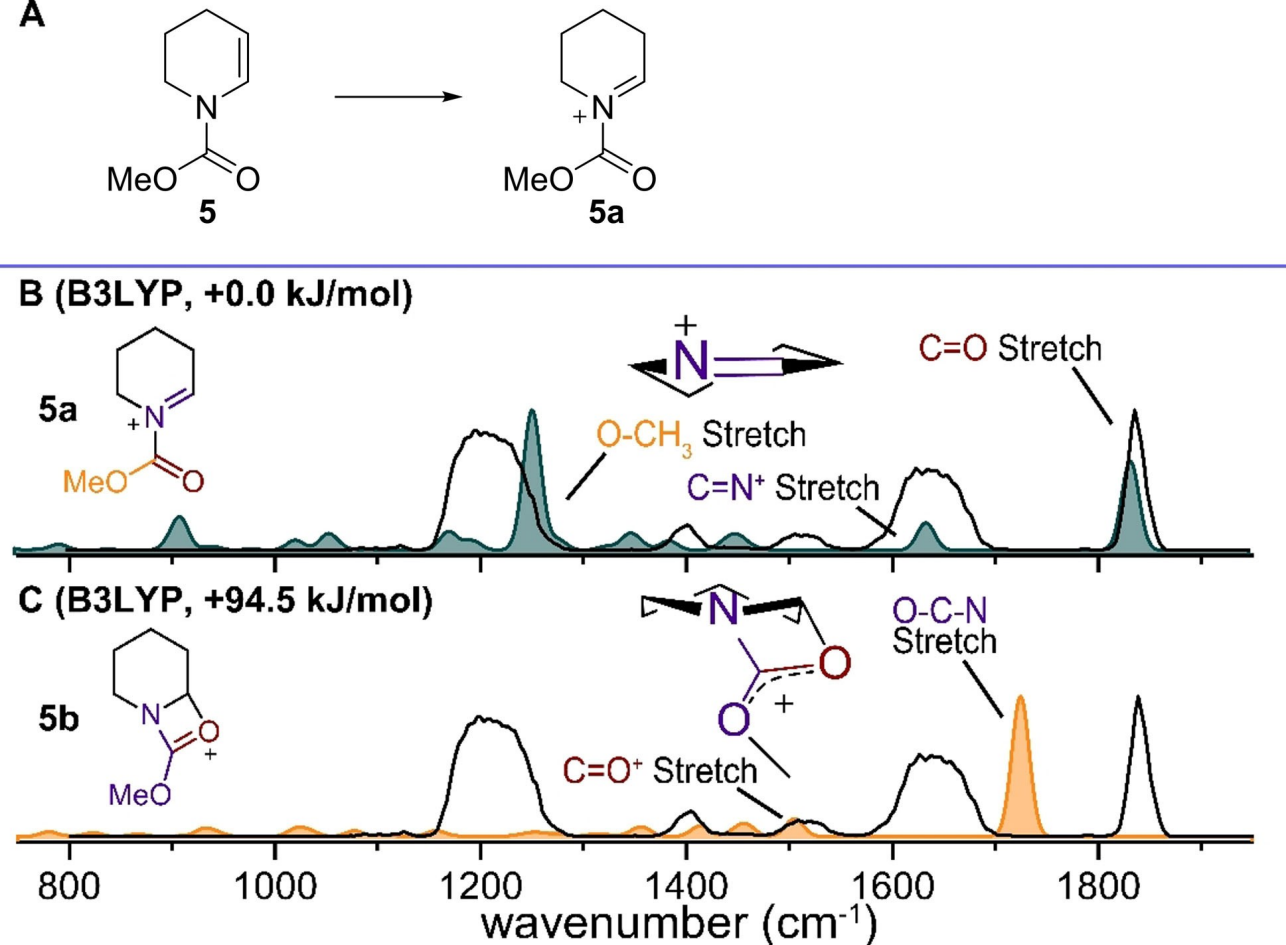
A) Isolation of the NAI (m/z 142) derived from precursor **5**, corresponding to cation **5a**. B) Comparison of the experimental spectrum corresponding to m/z 142 (black line) with the DFT calculated spectrum (B3LYP) of cation **5a** (color filled). C) Comparison of the experimental spectrum corresponding to m/z 142 (black line) with the DFT calculated (B3LYP) spectrum of cation **5b** (color filled). Energies are relative to the lowest‐energy structure, carbamate omitted for clarity in the 3D schematic structures.

To examine the utility of IRIS for the characterization of NAIs in the gas phase we first investigated NAI precursor **5**. Enamine **5** was ionized by ESI and the corresponding [M+H]^+^ ion (*m/z* 142) was isolated (Figure S1) and characterized by IRIS using the FELIX laser operating in the 800–1880 cm^−1^ range (Figure 1).^[8]^ Assignment of diagnostic vibrational bands in the experimental IR spectrum (Figure 1, black line) was achieved by comparison to calculated IR spectra of selected low‐energy structures (Figure 1, color filled lines). Quantum‐chemically predicted vibrational spectra were obtained using the B3LYP density functional and 6‐31++G(d,p) basis set, with starting geometries generated using a previously reported workflow.[^9b^, ^11^] Gibbs free energies (T=298 K) of the final geometries are based on the thermal energy from the frequency analysis combined with the electronic energy of a 2^nd^ order Møller‐Plesset (MP2) single‐point calculation (on the B3LYP geometry). In specific cases, the B3LYP functional was unable to reproduce the observed through‐space interactions and resulting vibrational modes. Therefore, the M06‐2x functional with the same basis set was employed to reoptimize the structure and calculate IR spectra, where the B3LYP geometry was used as a starting point. For structures where the B3LYP and M06‐2x results were ambiguous, a final assessment was made by performing a minimization and frequency analysis using more expensive MP2 calculations using the same basis set. Harmonic vibrational frequencies were scaled with a factor of 0.975 (B3LYP, MP2) or 0.945 (M06‐2x) to account for anharmonicity, unless stated otherwise (see below).

The IR spectrum of the NAI derived from **5** showed various diagnostic signals allowing for the assignment of its structure. For example, peaks corresponding to a C=O stretch (1830 cm^−1^) and C=N^+^ stretch (1636 cm^−1^) were observed and matched well with the calculated IR spectrum of NAI **5a** (Figure 1B). Involvement of the *N*‐acyl group in NAI stabilization (viz. structure **5b**) can be excluded on the basis of an expected significant blueshift of the carbamate vibration, which is not observed experimentally, and the higher calculated energy of +94.6 kJ/mol (Figure 1C). In contrast, the conformational flexibility of the unsubstituted piperidine ring results in deviations in the 1300 to 1375 cm^−1^ and 1480–1580 cm^−1^ region, bands attributed to various C−H bending modes of the piperidine ring, for both calculated structures.

**Figure 2:**
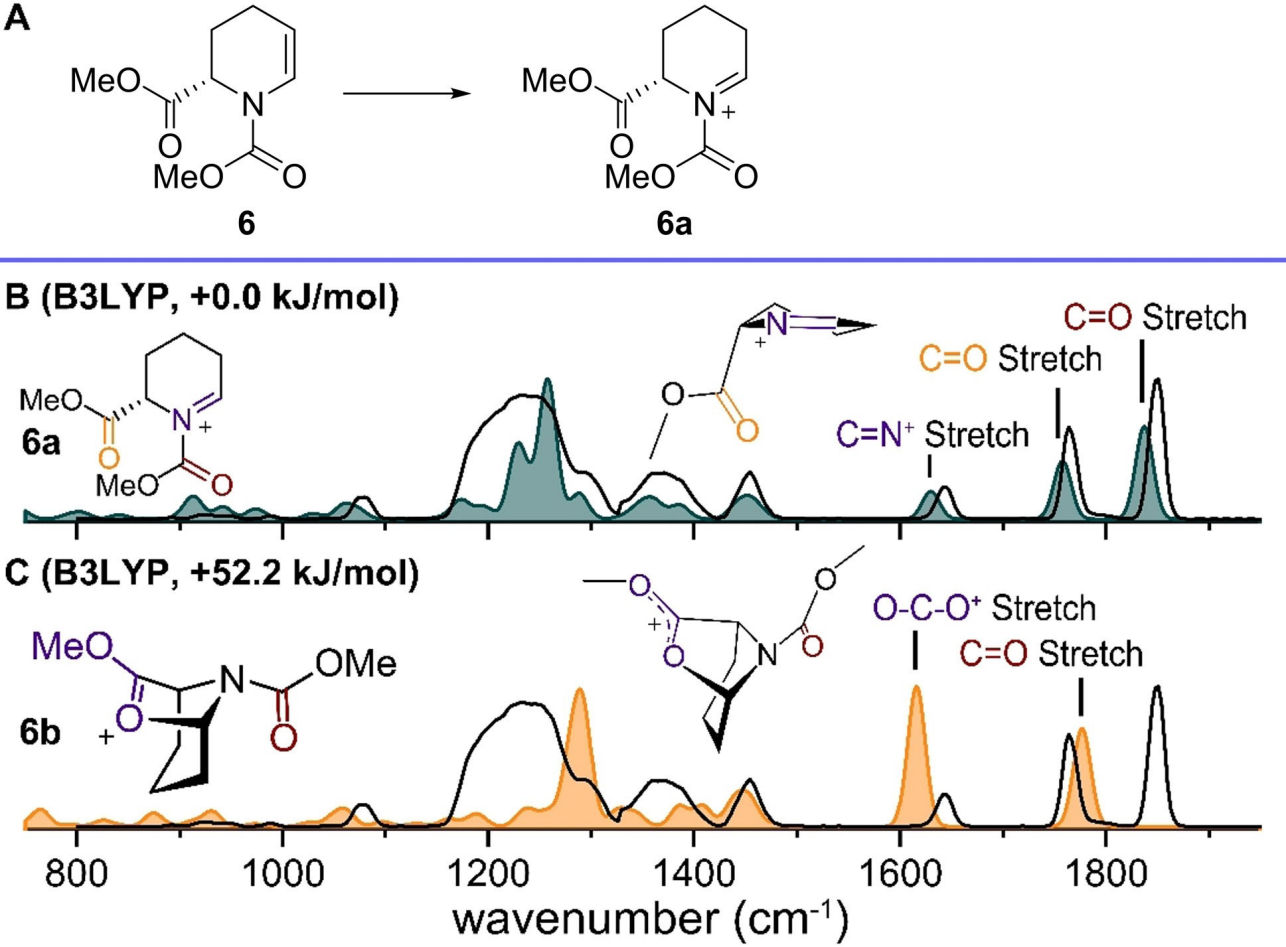
A) Isolation of the ionized of precursor **6** (m/z 200) corresponding to cation **6a**. B) Comparison of the experimental spectrum corresponding to m/z 200 (black line) with the DFT calculated (B3LYP) spectrum of cation **6a** (color filled). C) Comparison of the experimental spectrum corresponding to m/z 200 (black line) with the DFT calculated (B3LYP) spectrum of cation **6b** (color filled). Energies are relative to the lowest‐energy structure, carbamate omitted for clarity in the 3D schematic structure in B.

Next, we investigated enamine **6**, which is substituted at the 2‐position with a carboxylic acid ester to probe its effect on NAI structure and conformation. ESI of **6** (Figure S2) afforded its NAI [M+H]^+^ at *m/z* 200 and was characterized using IRIS. Three distinct bands in the region 1500–1850 cm^−1^ were observed, corresponding to two carbonyl stretches expected for the carbamate and ester and one C=N^+^ stretch vibration. Comparison with the calculated spectrum of the lowest‐energy structure **6a** (Figure 2B) shows excellent overlap with these bands. The conformation of **6a** induces a pseudo‐axial position of the 2‐methyl ester, which likely reduces its electron‐withdrawing effect and allows for stabilization as the ester carbonyl points towards the cationic center. Although the ester appears to stabilize the cationic center, it does so in a non‐covalent manner as a covalent interaction (**6b**) would lead to the absence of the diagnostic carbonyl stretch at 1830 cm^−1^, which is not observed experimentally (Figure 2C). Moreover, the significantly higher calculated energy of **6b** also allows us to safely discard this structure.

**Figure 3:**
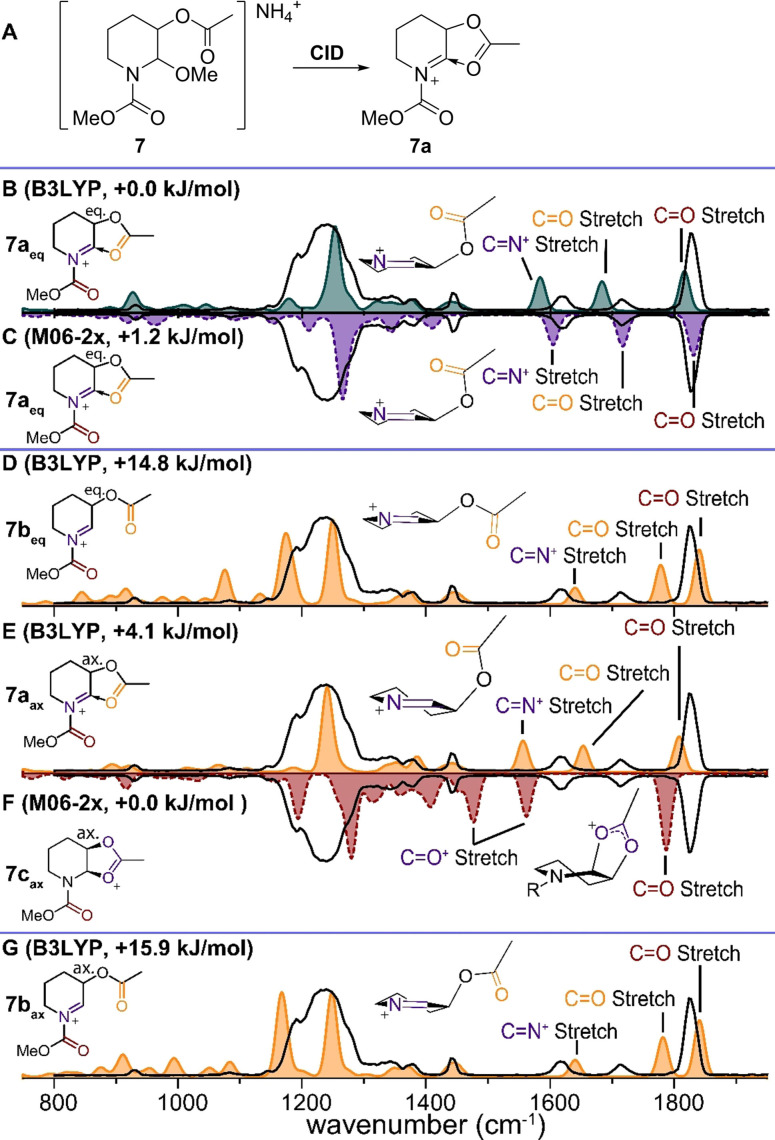
A) CID of precursor **7** results in fragmentation to *m/z* 200, corresponding to the mass of cation **7a**. Comparison of the IR spectrum of *m/z* 200 (black line) with the DFT calculated cations: B) **7a_eq_** (B3LYP). C) **7a_eq_** (M06‐2x). D) **7b_eq_** (B3LYP). E) **7a_ax_** (B3LYP). F) **7c_ax_** (M06‐2x). G) **7b_ax_** (B3LYP). Energies are relative to the lowest‐energy structure, carbamate omitted for clarity in the 3D schematic structures.

*N*‐Acylpiperidines bearing an acetyl group at the 3‐position react predominantly with 2,3‐*trans* selectivity, often attributed to participation of the neighboring ester as also observed in the corresponding glycosylation reactions.^[5b]^ To investigate the potential formation of dioxolenium species we turned our attention to *N*,*O*‐acetal **7** (Figure 3A). An MS/MS scheme was used to generate the NAI derived from **7**. ESI and collision induced dissociation (CID) of the parent ion, [M+NH_4_]^+^ at *m/z* 249, resulted in a fragment corresponding to the NAI at *m/z* 200, which was isolated (Figure S3) and characterized by IRIS. Again, three distinct vibrations above 1500 cm^−1^ were observed corresponding to two carbonyl stretches at 1820 and 1710 cm^−1^ (carbamate and acetyl ester, respectively) as well as a vibration at 1610 cm^−1^ attributed to the C=N^+^ stretch of the NAI.

The experimental IR spectrum aligns reasonably well with the predicted spectrum for the minimum‐energy structure of the set, NAI **7a_eq_** (Figure 3B, solid blue line), where the acetyl ester is positioned pseudo‐equatorially with its carbonyl pointing towards the cationic center. The through‐space stabilization of the ester in structure **7a_eq_** results in a significant energy lowering compared to the NAI **7b_eq_**, in which the neighboring acetyl is directed away from the cationic center (Figure 3D, +14.8 kJ/mol). The through‐space stabilization by the ester (**7a_eq_**) results in a redshift of the acetyl ester C=O stretch, resulting in a 100 cm^−1^ difference between **7a_eq_** and **7b_eq_** for this particular band. In addition, a 50 cm^−1^ redshift is observed for the C=N^+^ vibration. Hence, based on these redshifts it is likely that the acetyl group points towards the cationic center, yet the extent of this stabilization is overestimated by the B3LYP calculations. To better approximate the extent of stabilization in structure **7a_eq_** and **7b_eq_** the M06‐2x functional was employed, which is specifically parametrized for through‐space interactions. This level of theory reveals a significantly better match for the C=O stretch (1715 cm^−1^) and C=N^+^ vibration (1605 cm^−1^) for structure **7a_eq_** (Figure 3C, mirrored dashed purple line), although the match below 1500 cm^−1^ is relatively poor, as shown for example by the deviations near 1410 cm^−1^. The evaluation of **7b_eq_** with M06‐2x did not reveal a significant shift (Figure S4), so that we assign **7a_eq_** as the better fitting conformer. In addition, pseudo‐axial placement of the C‐3 CH bond may aid stabilization via hyperconjugation as postulated for oxycarbenium ions.^[12]^

We also considered conformers where the acetyl group is oriented pseudo‐axially, in which the through‐space stabilization of **7a_ax_** (Figure 3E, +4.1 kJ/mol) is lowest in energy. Comparison of the calculated C=O stretch of the acetyl (1775 cm^−1^) and C=N^+^ stretch (1570 cm^−1^) with the measured spectrum reveals a significantly poorer match as compared with the equatorially oriented acetyl **7a_eq_** (Figure 3B). When employing M06‐2x to better approximate the through‐space interaction of **7a_ax_**, its geometry optimized to the covalent conformer **7c_ax_** (Figure 3F). Although this conformer is slightly lower in energy than conformer **7a_eq_** according to M06‐2x (−1.2 kJ/mol), its predicted spectrum clearly deviates from the experimental spectrum. For example, the dioxolenium stretches at 1610 and 1520 cm^−1^ are not reproduced in the experimental spectrum and we can therefore exclude this conformer from consideration. Finally, the pseudo‐axially oriented conformer **7b_ax_** is considered (Figure 3G), where the acetyl group is directed away from the cationic center. While excellent spectral overlap is found below 1500 cm^−1^, the diagnostic bands attributed to the C=N^+^ stretch (1640 cm^−1^) and in particular the acetyl C=O stretch (1835 cm^−1^) are clearly not matching. As before, evaluation with M06‐2x did not result in a significantly better match (Figure S4). Taken together, comparison of our data resulting from CID of **7** with the calculated spectra of conformers with either pseudo‐axial or pseudo‐equatorial orientation of the acetyl group, we can confidently assign conformer **7a_eq_** as the best match, where the charge of the NAI is stabilized through space by the neighboring acetyl group.

**Figure 4:**
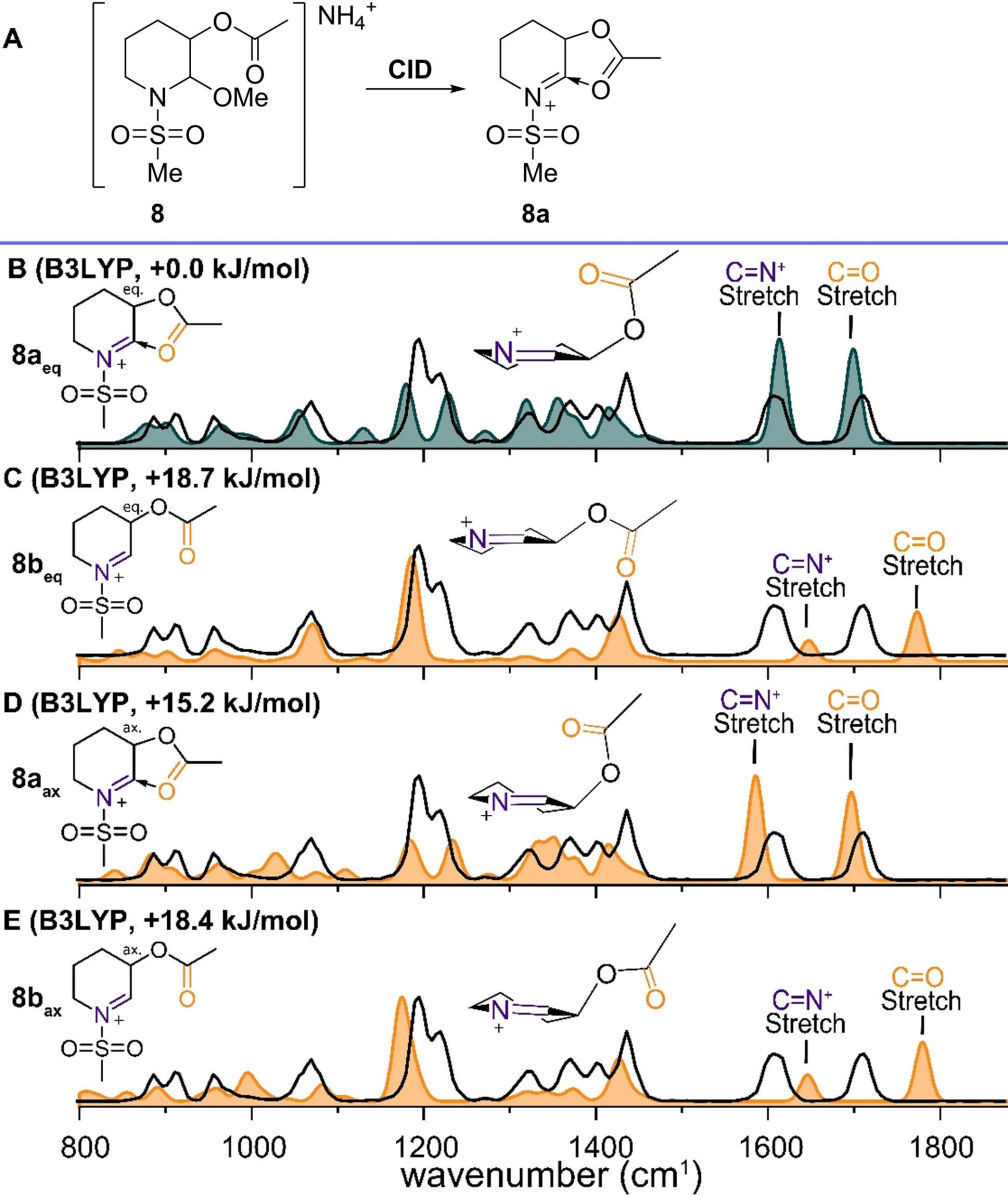
A) CID of precursor **8** results in fragmentation to m/z 220, corresponding to the mass of cation **8a**. Comparison of the spectrum m/z 220 (black line) with the DFT calculated (color filled) cations. B) **8a_eq_** (B3LYP). C) **8b_eq_** (B3LYP). D) **8a_ax_** (B3LYP). E) **8b_ax_** (B3LYP). Energies are relative to the lowest‐energy structure, carbamate omitted for clarity in the 3D schematic structures.

To further investigate the effect of the *N*‐acyl group on the through‐space stabilization by the C‐3 ester, we investigated *N*,*O*‐acetals bearing a more electron‐withdrawing methylsulfonate group. MS/MS afforded the NAI derived from precursor **8** (*m/z* 220, Figure S5), which was subsequently characterized by IRIS. Two diagnostic peaks above 1600 cm^−1^ are characteristic of an acetyl C=O and C=N^+^ stretch (Figure 4B, black line). Since sulfonate vibrations are not modelled well using B3LYP with a conventional scaling factor of 0.975, the vibrations involving sulfur were scaled by 1.028 (Figures S9 and S10), based on previous observations for benchmark systems.^[13]^ The lowest‐energy structures for NAIs derived from **8** involved through‐space stabilization of the pseudo‐equatorial C‐3 acetyl as observed for precursor **7**. Indeed, the B3LYP calculated spectrum of the through‐space stabilized structure **8a_eq_** closely reproduces the major diagnostic peaks corresponding to the C=O (1710 cm^−1^) and C=N^+^ (1610 cm^−1^) stretch modes (Figure 4B), resulting in an excellent match. In contrast, for conformer **8b_eq_** in which the acetyl group points away from the NAI cationic center, the ester carbonyl stretch is calculated at 1790 cm^−1^ and the C=N^+^ stretch is shifted by 30 cm^−1^ to 1640 cm^−1^ with respect to the global minimum energy conformation **8a_eq_**. Evaluating the pseudo‐axial conformers, **8a_ax_** and **8b_ax_**, a similar trend was observed with the structure enjoying through‐space stabilization (**8a_ax_**) in better agreement with the experimental data. Whilst the pseudo‐axial conformer **8a_ax_** (Figure 4D) shows excellent spectral overlap with the measured data below 1500 cm^−1^, the C=N^+^ stretch (1585 cm^−1^) and the C=O stretch of the acetyl (1695 cm^−1^) are in poorer agreement compared to **8a_eq_** (Figure 4B). Stabilizing the cation by increasing the electron‐withdrawing nature of the substituent leads to more pronounced through‐space stabilization by the neighboring acetyl group.

**Figure 5:**
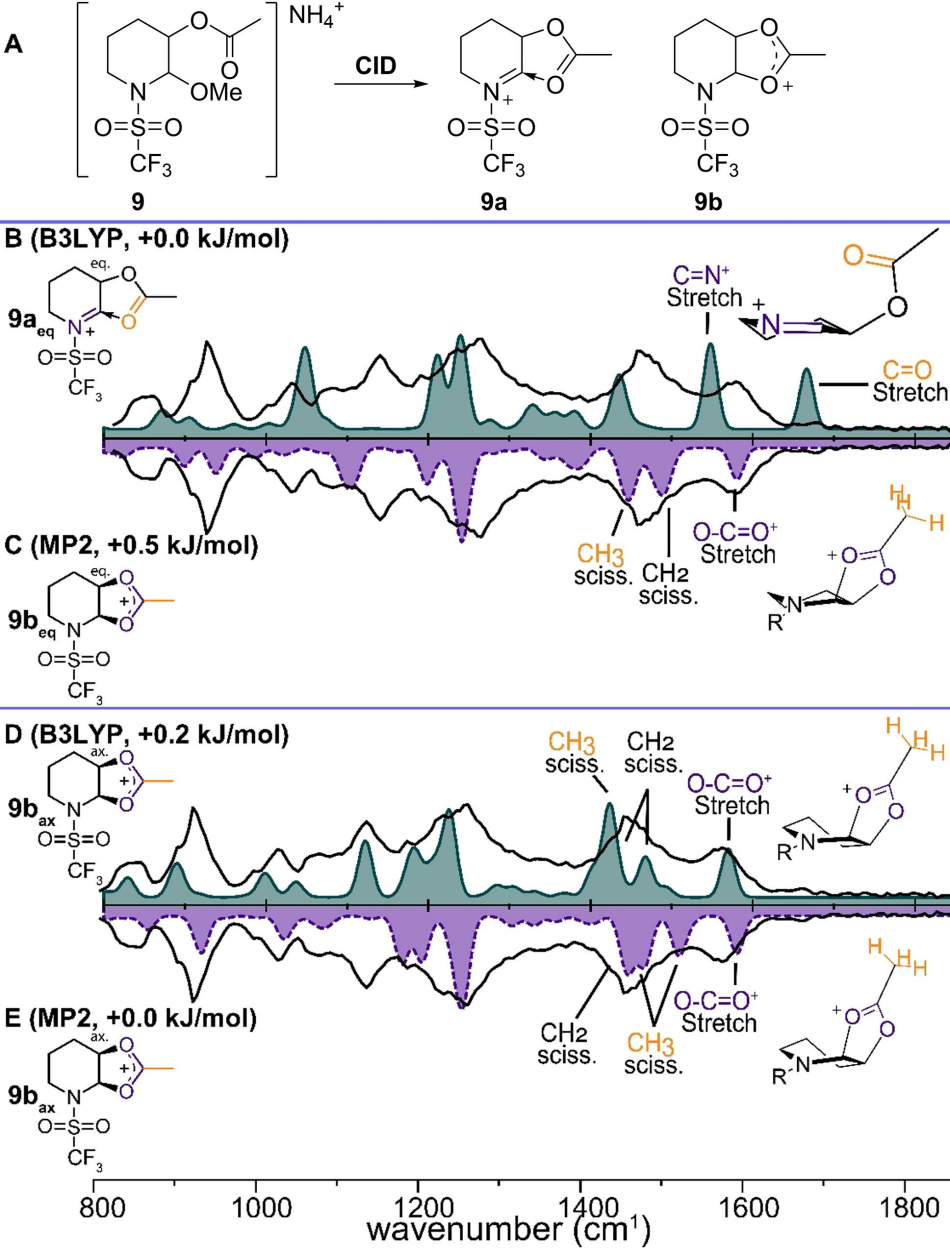
A) CID of precursor **9** results in fragmentation to m/z 273, corresponding to the mass of cations **9a**–**c**. Comparison of the spectrum m/z 273 (black line) with the DFT calculated spectrum of cations **9a** and **9b** (color filled) of: B) **9a_eq_** (B3LYP) C) **9b_eq_** (MP2). D) **9b_ax_** (B3LYP). E) **9b_ax_** (MP2). Energies are relative to the lowest‐energy structure, carbamate omitted for clarity in the 3D schematic structures.

To investigate this effect further, we prepared precursor **9** containing a trifluoromethanesulfonyl group. The IRIS spectrum resulting from the MS/MS product of **9** (*m/z* 273, Figure S11) displays broad peaks in the 1400–1600 cm^−1^ range (Figure 5B, black line). The lack of sharp bands could indicate a mixture of (interconverting) conformers. The two broad bands in the 1400–1600 cm^−1^ range are indicative of a dioxolenium species, further corroborated by the absence of a diagnostic acetyl C=O stretch in the 1650–1800 cm^−1^ range. B3LYP calculations reveal two iso‐energetic conformers, **9a_eq_** and **9b_ax_**. Comparison of the B3LYP calculated spectra with experiment suggests a better match for dioxolenium ion **9b_ax_** (Figure 5D, light blue line). The dioxolenium OCO stretches at 1570 cm^−1^ and 1465 cm^−1^, the CH_2_ scissoring modes of the rigidified piperidine ring at 1445, 1470 and 1490 cm^−1^ as well as characteristic vibrations of the methyl group (1400 and 1420 cm^−1^) show good overlap for **9b_ax_**. In contrast, the predicted spectrum for conformation **9a_eq_** (Figure 5B, light blue line) displays a clear mismatch of the acetyl C=O stretch (1650 cm^−1^). The negligible computed energy difference (0.2 kJ/mol) combined with the significantly different computed IR spectra, prompted further computational investigation at the MP2 level. A geometry optimization starting from **9a_eq_** and **9b_ax_** both results in a dioxolenium conformation, where equatorial attack results in conformation **9b_eq_** (Figure 5C) and axial attack results in the half chair conformation **9b_ax_** (Figure 5E). The negligible energy difference (0.5 kJ/mol) combined with the broadened bands in the experimental spectrum suggest that a mixture of both **9b_eq_** and **9b_ax_** is present, likely with a low interconversion barrier.

**Scheme 2:**
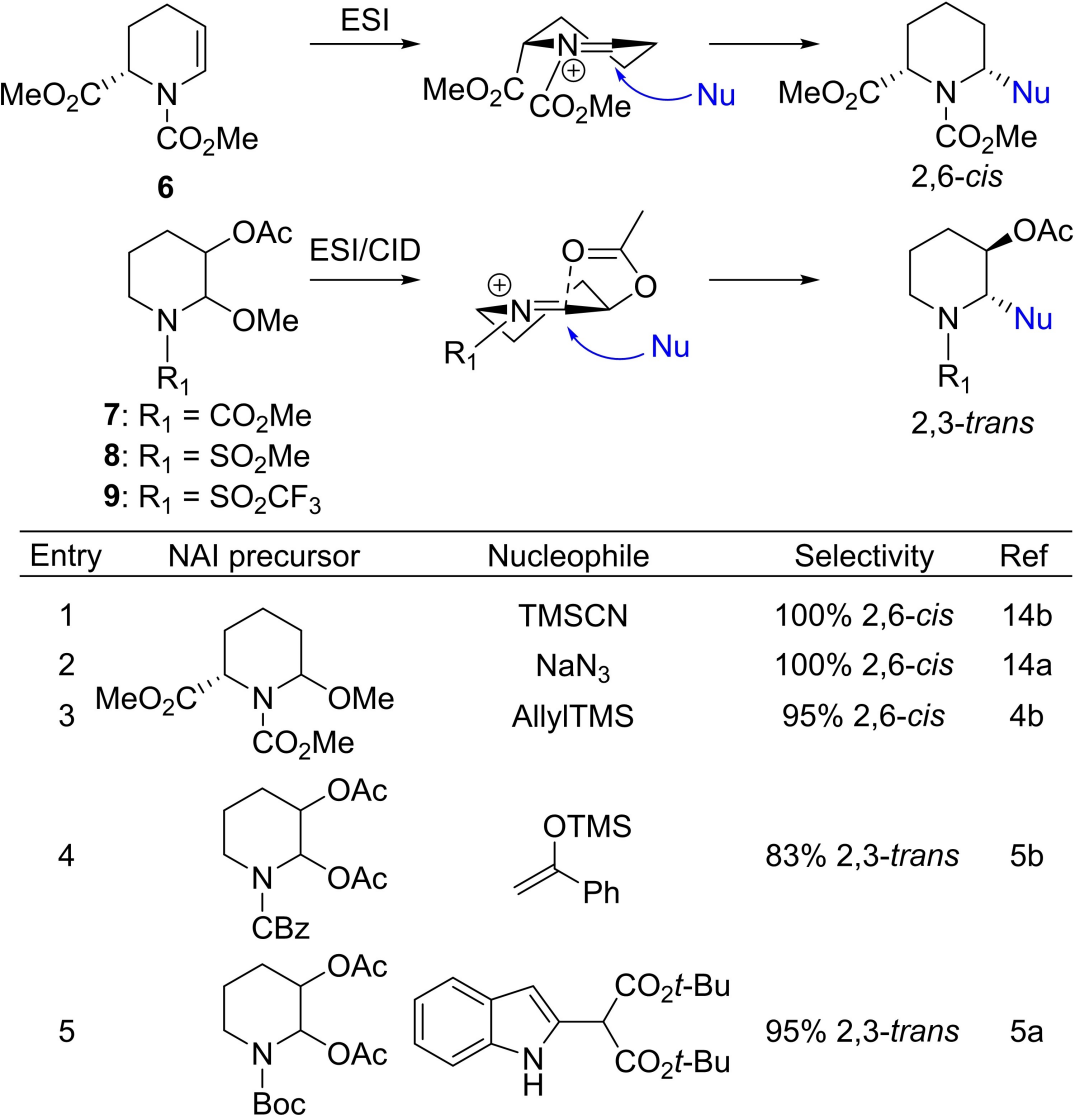
NAI structures characterized by IRIS and the expected facial selectivity of the nucleophile approach (blue). Observed selectivity of nucleophilic addition in the solution phase (Table).

The characterized conformations of 2‐ (**6**) and 3‐substituted (**7**–**9**) NAIs are expected to lead to *cis‐* and *trans*‐addition of the nucleophile, respectively (Scheme 2). Comparison with similar NAI precursors substituted at the 2‐ (entries 1–3)[^4b^, ^14^] or 3‐position (entries 4–5)[^5a^, ^5b^] that have been reacted with nucleophiles in solution indeed confirm this expected facial selectivity. Although this does not necessarily mean that the gas‐phase NAIs are the same as those present in solution, these results demonstrate good correlations in these examples.

**Figure 6:**
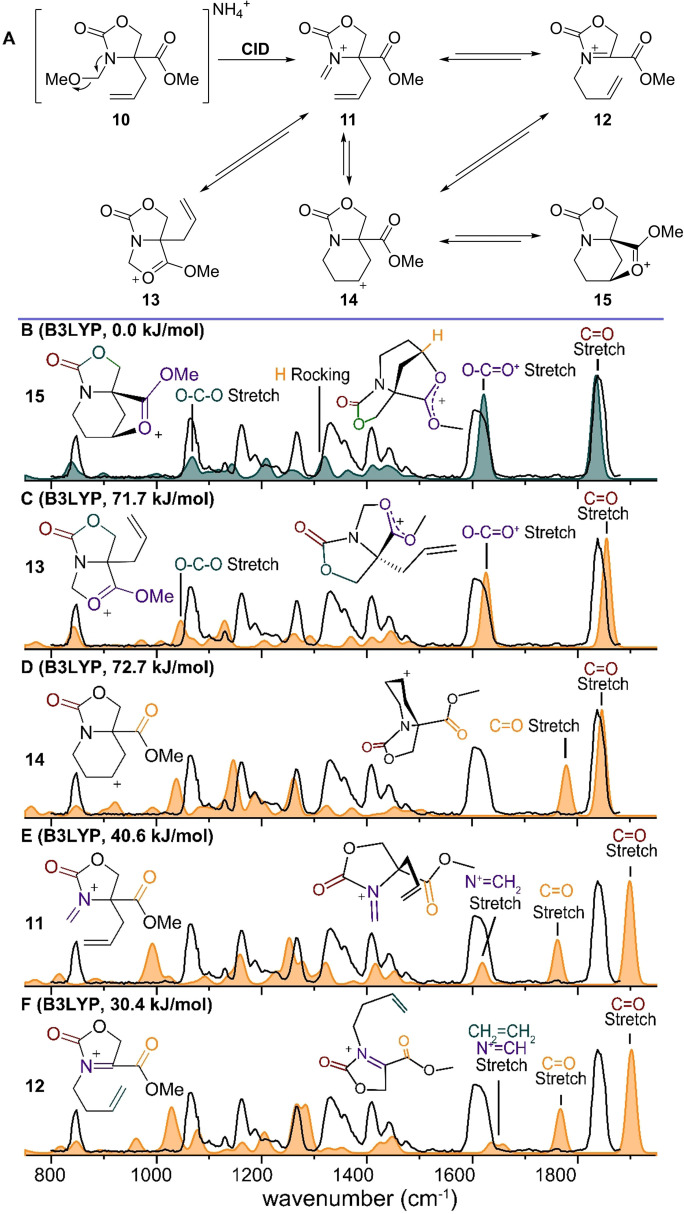
CID of precursor **10** results in fragmentation to m/z 198, corresponding to the mass of cations **11**–**15**. Comparison of the spectrum m/z 273 (black line) with the DFT calculated cations **11**–**15** (color filled) of: B) **15** (B3LYP). C) **13** (B3LYP). D) **14** (B3LYP). E) **11** (B3LYP). F) **12** (B3LYP). Energies are relative to the lowest‐energy structure, carbamate omitted for clarity in the 3D schematic structures.

Finally, we investigated NAI rearrangements in the gas phase. We anticipated that NAI **11** derived from precursor **10** could rearrange via pathways akin to a 2‐aza‐Cope or aza‐acyliminium Prins rearrangement followed by ester participation (Figure 6).^[15]^ Generation of NAI **11** (*m/z* 198, Figure S6) was achieved via CID MS/MS of electrosprayed **10** and the resulting cation was characterized using IRIS. The experimentally observed spectrum contains two distinct bands above 1500 cm^−1^, one indicative of a carbonyl stretch (1820 cm^−1^), while the feature at 1600 cm^−1^ could involve a C=N^+^ stretch or a dioxolenium O−C=O^+^ stretch. The two structures most consistent with the experimental spectrum result from acetyl participation before (**13**) or after rearrangement (**15**), as their calculated IR spectra reproduce these diagnostic features best. The initially formed NAI **11** and intermediates in the rearrangement, **12** and **14**, are not detected on the basis of poor spectral overlap in the region above 1500 cm^−1^ (see Figures 6D, 6E and 6F). In contrast, the calculated spectra of ions **13/15** (Figures 6B and 6C) show an excellent overlap with the measured spectrum, in particular the characteristic O−C=O^+^ stretch (1620 cm^−1^) of the dioxolenium ion as well the predicted C=O stretch (1820–1840 cm^−1^) of the carbamate. As has been observed in the previous system, stabilization by formation of a dioxolenium ion of the nitrogen centered cation is not favored over through‐space stabilization. In addition, the relative energy of the dioxolenium stabilized ion **13** (Figure 6C, 71.7 kJ/mol) is significantly higher than that of NAI **11** (Figure 6E, 40.6 kJ/mol) and, in line with our previous findings, covalent stabilization of the NAI is not expected; therefore, we can safely exclude structure **13** from consideration. In contrast, covalent stabilization was found to be significantly lower in energy for the dioxolenium ion **15** (Figure 6B, 0.0 kJ/mol) versus the carbon‐centered cation **14** (Figure 6D, 72.7 kJ/mol). Closer examination of this calculated spectrum reveals particularly intense vibrations of the C−H bond attached to the carbon stabilized by the dioxolenium ion (Figure 6B, 1320 cm^−1^) as well as the O−C−O stretch of the cyclic carbamate (Figure 6B, 1070 cm^−1^), which are well represented in the experiment. Hence, we conclude that generation of NAI **11** leads to a rearrangement to structure **15** in the gas phase.

## Conclusion

In conclusion, we generated and isolated *N*‐acyliminium ions by ESI and CID in the gas phase and characterized them by IR ion spectroscopy in combination with quantum‐chemical calculations. The diagnostic vibrations yield valuable insights in the conformation of the ions in the absence of counter ions and solvent. We observed that the resulting *N*‐acyliminium ions are generally stabilized by esters through space, by the pseudo‐equatorial oriented acetyl group, but do not form dioxolenium ions except in case the more electron‐withdrawing *N*‐triflate group was employed. Although the B3LYP functional in general performed well in reproducing the experimental IR spectra, the through‐space interaction specifically was better modeled by M06‐2x or higher‐level MP2 calculations. Only in the case of an NAI rearrangement in the gas phase, leading to a secondary carbocation that is no longer stabilized by an adjacent nitrogen atom, a dioxolenium ion was formed, indicating that more reactive cations are needed to enable the formation of dioxolenium ions. This study emphasizes the power of the IRIS methodology to study highly reactive intermediates and gain fundamental insights in their conformation, thereby contributing to the stereochemical model to understand and predict their reactivity.

## Experimental Section

See the Supporting Information for experimental procedures and analytical data.

## Conflict of interest

The authors declare no conflict of interest.

1

## Supporting information

As a service to our authors and readers, this journal provides supporting information supplied by the authors. Such materials are peer reviewed and may be re‐organized for online delivery, but are not copy‐edited or typeset. Technical support issues arising from supporting information (other than missing files) should be addressed to the authors.

Supporting Information

## Data Availability

The data that support the findings of this study are available from the corresponding author upon reasonable request.
