# Supplementary material for: Characterization of Cyclic N‐Acyliminium Ions by Infrared Ion Spectroscopy
Source: Chemistry. 2021 Dec 29;28(9):e202104078. doi: 10.1002/chem.202104078 (PMC9302692; doi:10.1002/chem.202104078)
Supplement: Supplementary file 1 — Supporting Information [file CHEM-28-0-s001.pdf]

Chemistry–A European Journal

Supporting Information

Characterization of Cyclic *N*-Acyliminium Ions by Infrared Ion Spectroscopy

Jona Merx, Kas J. Houthuijs, Hidde Elferink, Eva Witlox, Jasmin Mecinović, Jos Oomens, Jonathan Martens, Thomas J. Boltje,* and Floris P. J. T. Rutjes*

Table of Contents

Experimental details infrared ion spectroscopy and quantum chemical calculations.....................3

CID- and IR-spectra 4

General Synthetic Methods..13

^{1}H- and ^{13}C-NMR Spectra of Compounds 1-5 17

Gaussian Coordinates...29

Experimental details infrared ion spectroscopy and quantum chemical calculations

The purified samples were dissolved in ACN (1 ml) and further diluted with MeOH to yield 1 mM stock solutions. From these stock solutions 10 µl was added to MeOH/H_2O (1/1, 1.5 ml) containing 0,1 % ammonium acetate. The resulting solutions were infused in a 3D quadrupole ion trap (QIT) mass spectrometer (Bruker, Amazon Speed ETD, Bremen, Germany), which was coupled to the beamline of the free-electron laser FELIX. Positively charged ions generated by electrospray ionization (ESI) were m/z-isolated. Where specified, the ions were subjected to 40 ms of collision-induced dissociation, after which the fragment ion of interest was mass-isolated. The resulting N-acyliminium ions (NAIs) were then irradiated with 1 or 2 macropulses from FELIX at a repetition rate of 10 Hz, each pulse having an energy of 20 to 160 mJ and bandwidth of 0.5% of the center frequency. The absorption of infrared light increases the internal energy of the NAIs, leading to their unimolecular dissociation and the generation of fragments. An IR spectrum is constructed by monitoring the wavelength-dependent dissociation and calculating the fragmentation yield, $-\ln[1 - \sum I(\text{fragment ions})/\sum I(\text{all ions})]$. A linear laser power correction is applied to this yield and the wavelength is calibrated using a grating spectrometer.
Gas-phase geometries and corresponding IR absorption spectra of the presented ions were calculated using a previously reported workflow. SMILES structure formats of the NAIs of interested served as input for the workflow using the cheminformatics toolbox RDKit. After generating 500 conformations using the distance geometry algorithm with subsequent energy minimization with the MMFF94 force field, the 20 (30 for NAIs of **9** and **9**) most diverse structures were selected. This diversity was determined by performing hierarchical clustering on the root-mean-squared distance between geometries. The selected geometries were submitted to Gaussian 16 for geometry optimization and frequency calculations at the semi-empirical PM6 level. Geometries within 40 kJ/mol (80 kJ/mol for NAIs of **8** and **9**) of the minimum-energy geometry were optimized using density functional theory with the B3LYP functional and 6-31++G(d,p) basis set, after which a harmonic frequency calculation was performed. The Gibbs free energy of each geometry was computed by combining the thermal energy from the frequency calculation with the electronic energy from an MP2 single-point calculation at the B3LYP geometry, again using the 6-31++G(d,p) basis set. The IR frequencies were scaled by 0.975 as an approximate correction for anharmonicity, and convoluted with a Gaussian lineshape function with a full-width-at-half-maximum of 20 cm^{-1}. Additionally, IR spectra based on M06-2x and MP2 levels of theory with the 6-31++G(d,p) basis set were calculated for selected geometries. Final B3LYP structures were chosen as starting geometries, which were minimized with the selected method after which a vibrational analysis was performed. Presented Gibbs free energies are based on the combined electronic and thermal energy, both at the level of the selected method. For M06-2x and MP2 spectra were scaled with the factors 0.945 and 0.975, respectively. Vibrations involving sulfur atoms were scaled by a factor of 1.028 (independent of theory chosen), based on previous observations for benchmark systems (for B3LYP). Sulfur vibrations were selected by extracting the absolute movement of the sulfur atom in each normal mode from the Gaussian16 output file and using a threshold of 0.1 Å to assign the modes as a sulfur vibration.

CID- and IR-spectra

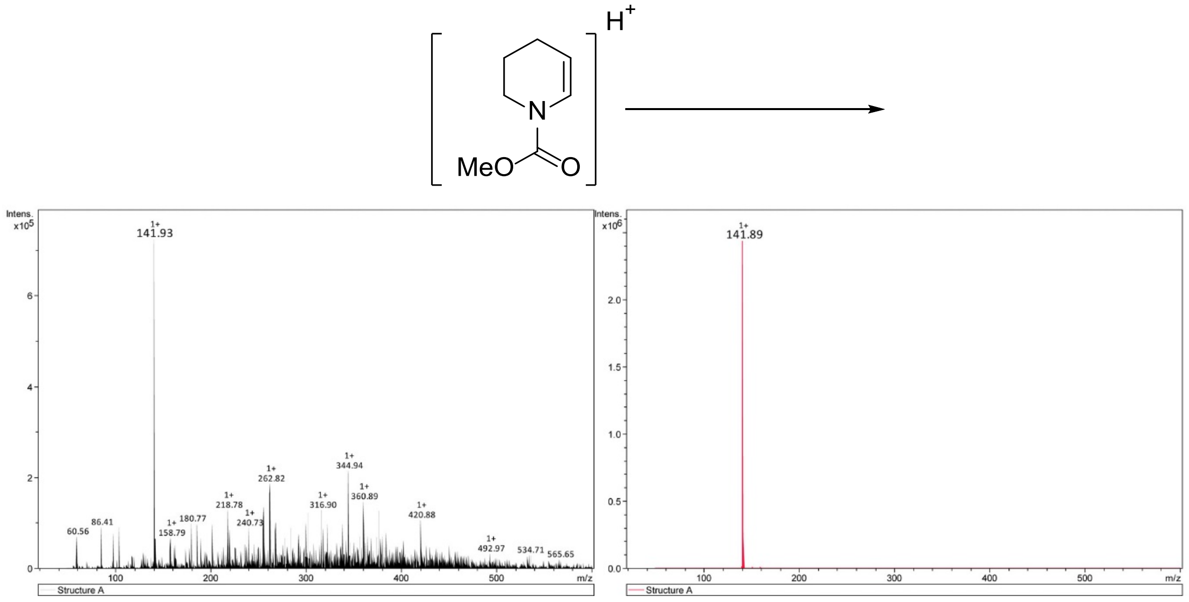

Figure S1. Isolation of compound **5**.

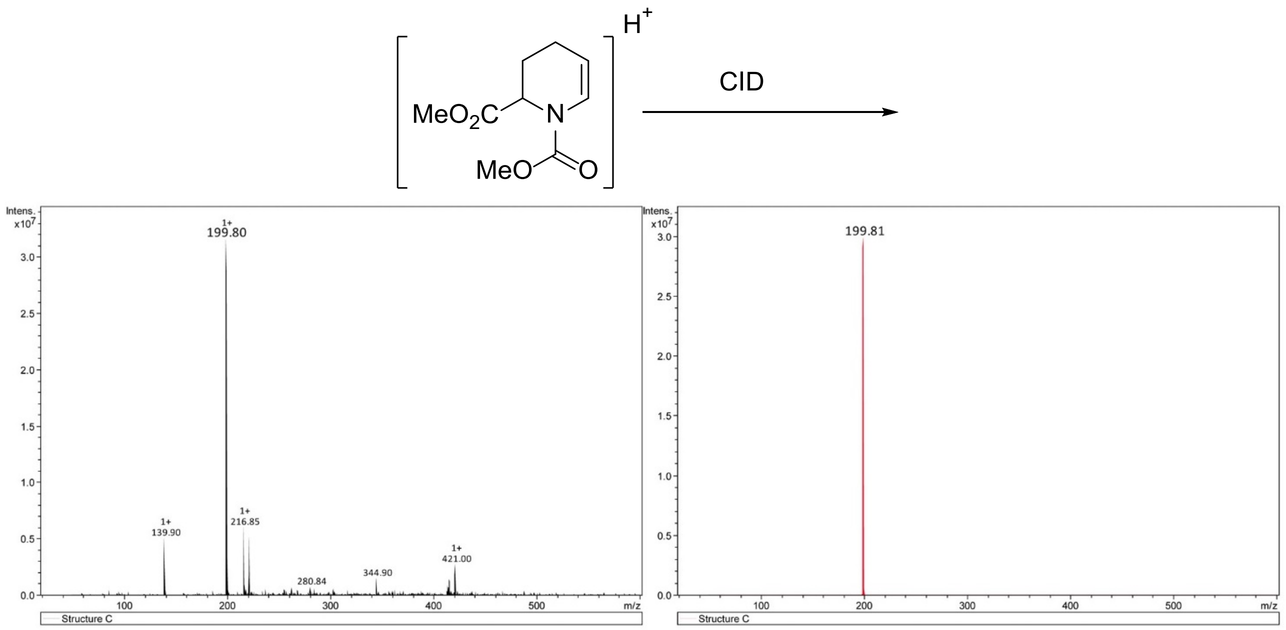

Figure S2. Isolation of compound **6**.

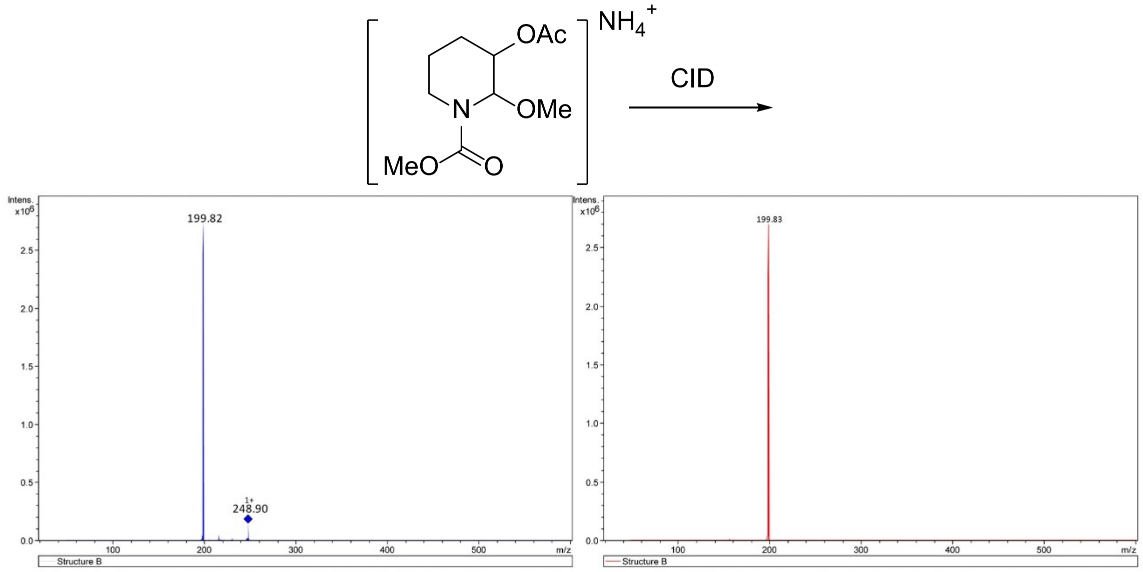

Figure S3: CID to give compound **7**

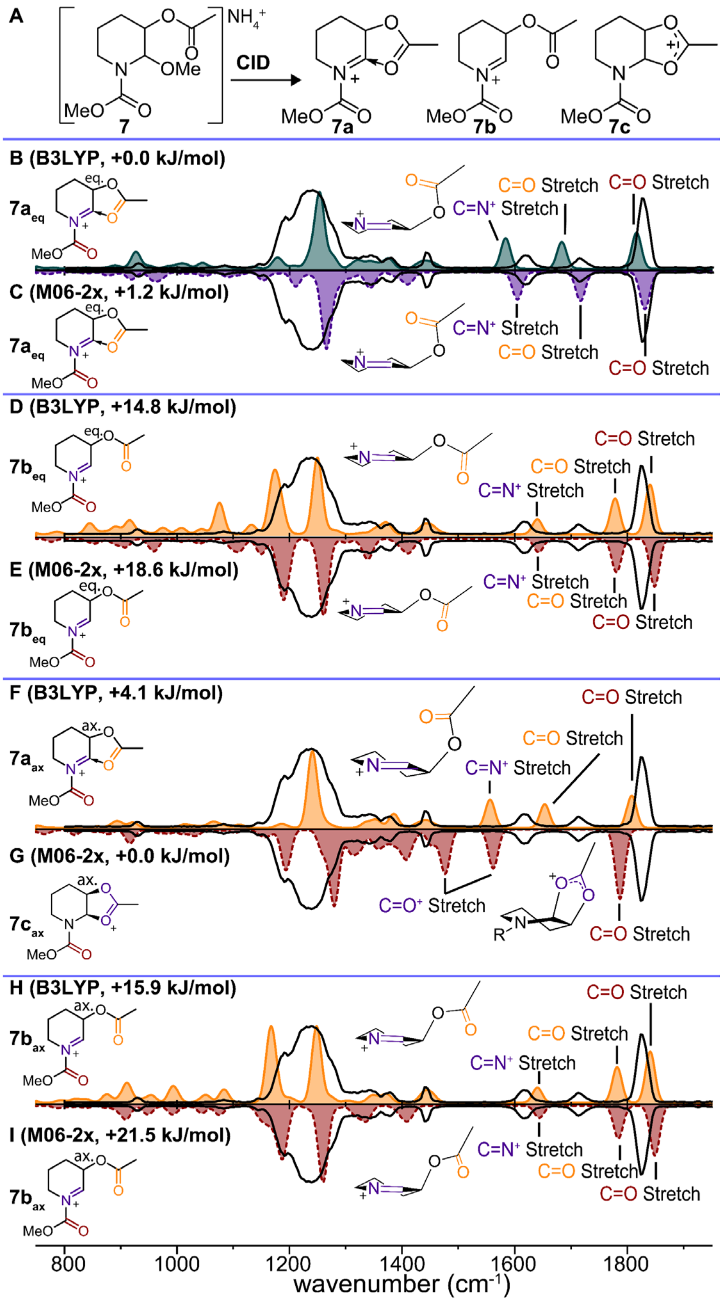

Figure S4 A) CID of precursor **7** results in fragmentation to m/z 200, corresponding to the mass of cations **7a-c**. Comparison of the spectrum m/z 200 (black line) with the DFT calculated cations **7a**, **7b** and **7c** (colour filled) of: B) B3LYP spectrum of cation $\mathbf{7a}_{\mathbf{eq}}$, C) MO6-2X spectrum of cation $\mathbf{7a}_{\mathbf{eq}}$ (mirrored in x-axis), D) B3LYP spectrum of cation $\mathbf{7b}_{\mathbf{eq}}$, E) MO6-2X spectrum of cation $\mathbf{7b}_{\mathbf{eq}}$ (mirrored in x-axis). F) B3LYP spectrum of cation $\mathbf{7a}_{\mathbf{ax}}$, G) MO6-2X spectrum of cation $\mathbf{7a}_{\mathbf{ax}}$ (mirrored in x-axis), H) B3LYP spectrum of cation $\mathbf{7b}_{\mathbf{ax}}$ and I) MO6-2X spectrum of cation $\mathbf{7b}_{\mathbf{ax}}$ (mirrored in x-axis). Energies are relative to the lowest-energy structure, carbamate omitted for clarity in the 3D schematic representation of the molecule.

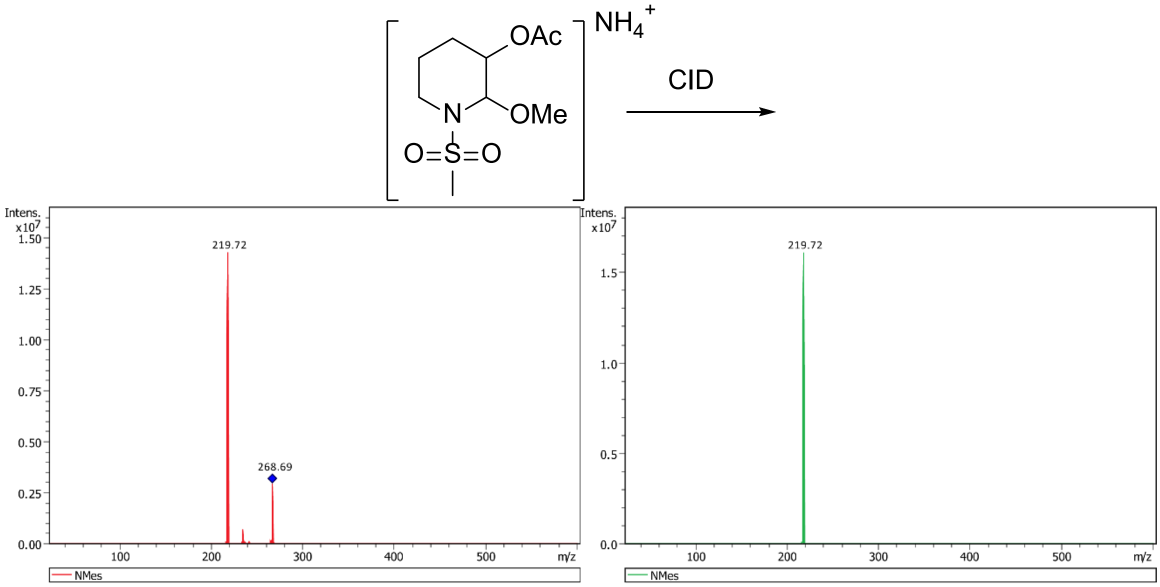
OAc
OMe
N
O=S=O
NH4+
CID
Intens. x10^7
219.72
268.69
m/z
NMes
219.72
NMes

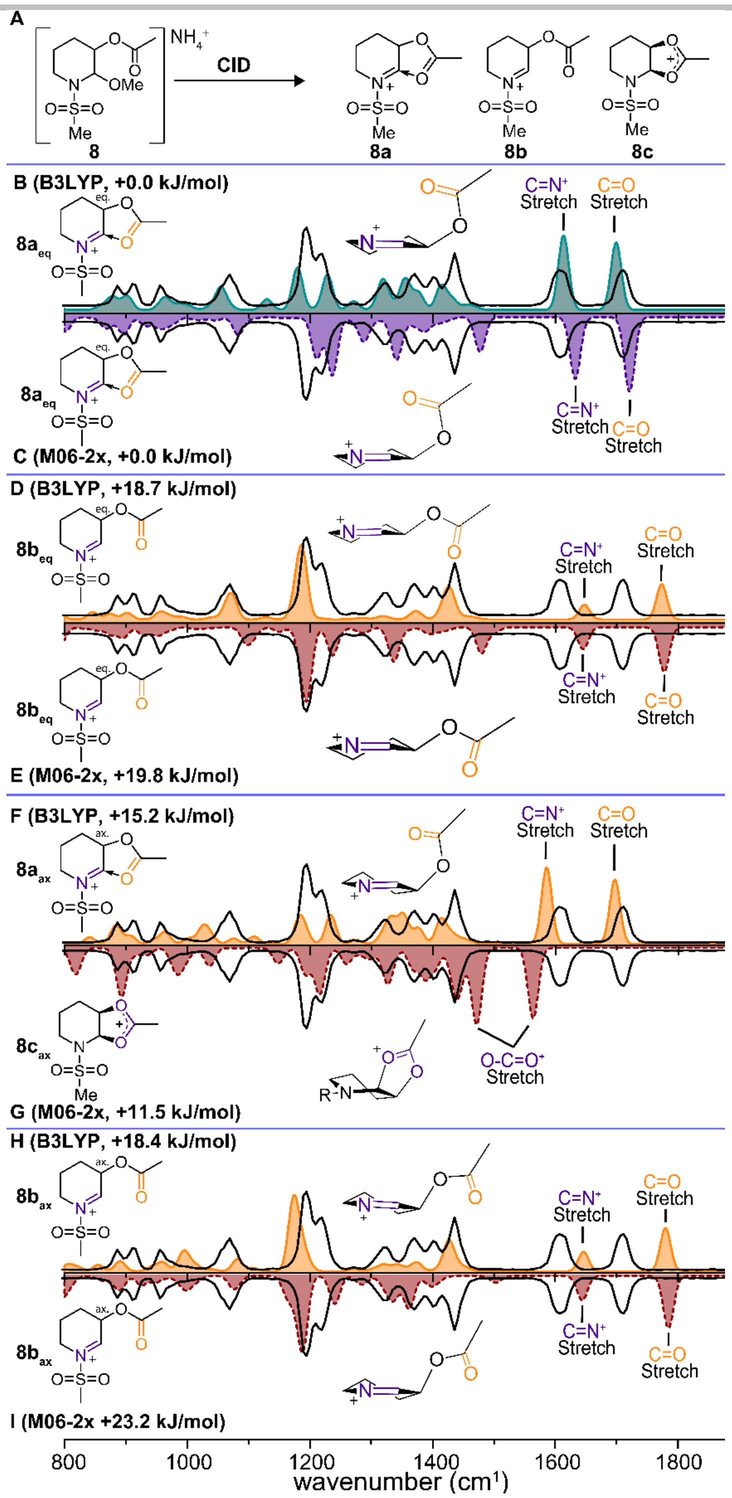

Figure S6: A) CID of precursor **8** results in fragmentation to m/z 220, corresponding to the mass of cations **8a-c**. Comparison of the spectrum m/z 220 (black line) with the DFT calculated cations **8a**, **8b** and **8c** (colour filled) of: B) B3LYP spectrum of cation **8a**$_{eq}$, C) MO6-2X spectrum of cation **8a**$_{eq}$ (mirrored in x-axis), D) B3LYP spectrum of cation **8b**$_{eq}$, E) MO6-2X spectrum of cation **8b**$_{eq}$ (mirrored in x-axis), F) B3LYP spectrum of cation **8a**$_{ax}$, G) MO6-2X spectrum of cation **8c**$_{ax}$ (mirrored in x-axis), H) B3LYP spectrum of cation **8b**$_{ax}$ and I) MO6-2X spectrum of cation **8b**$_{ax}$ (mirrored in x-axis). Energies are relative to the lowest-energy structure, carbamate omitted for clarity in the 3D schematic representation of the molecule.

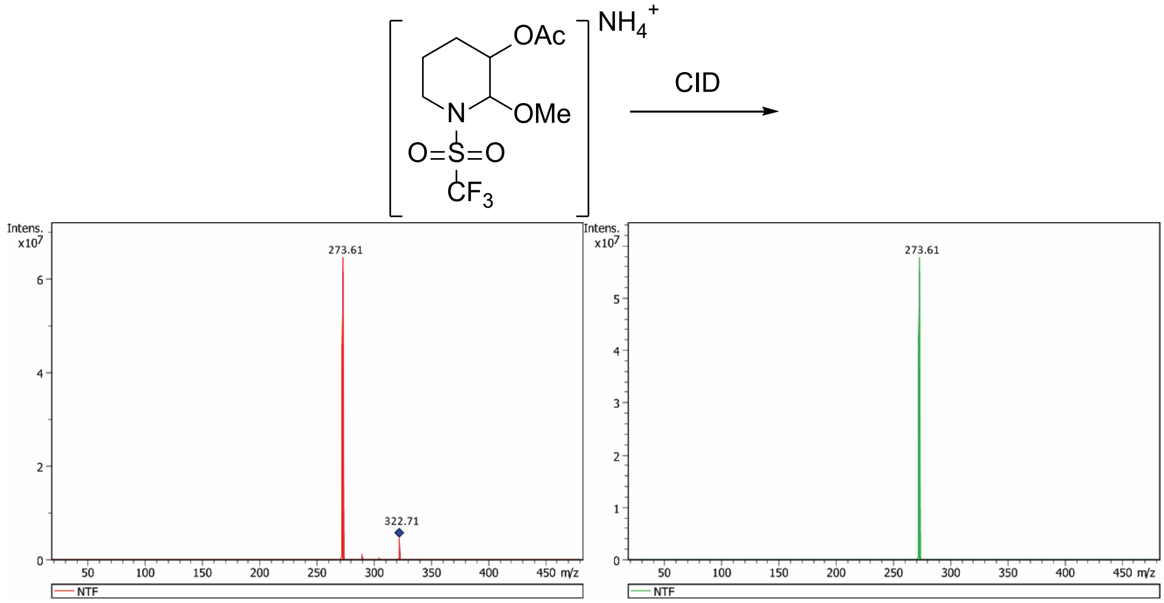
OAc
OMe
N
O=S=O
CF3
NH4+
CID
Intens.
x10^7
273.61
322.71
273.61
m/z
NTF
NTF

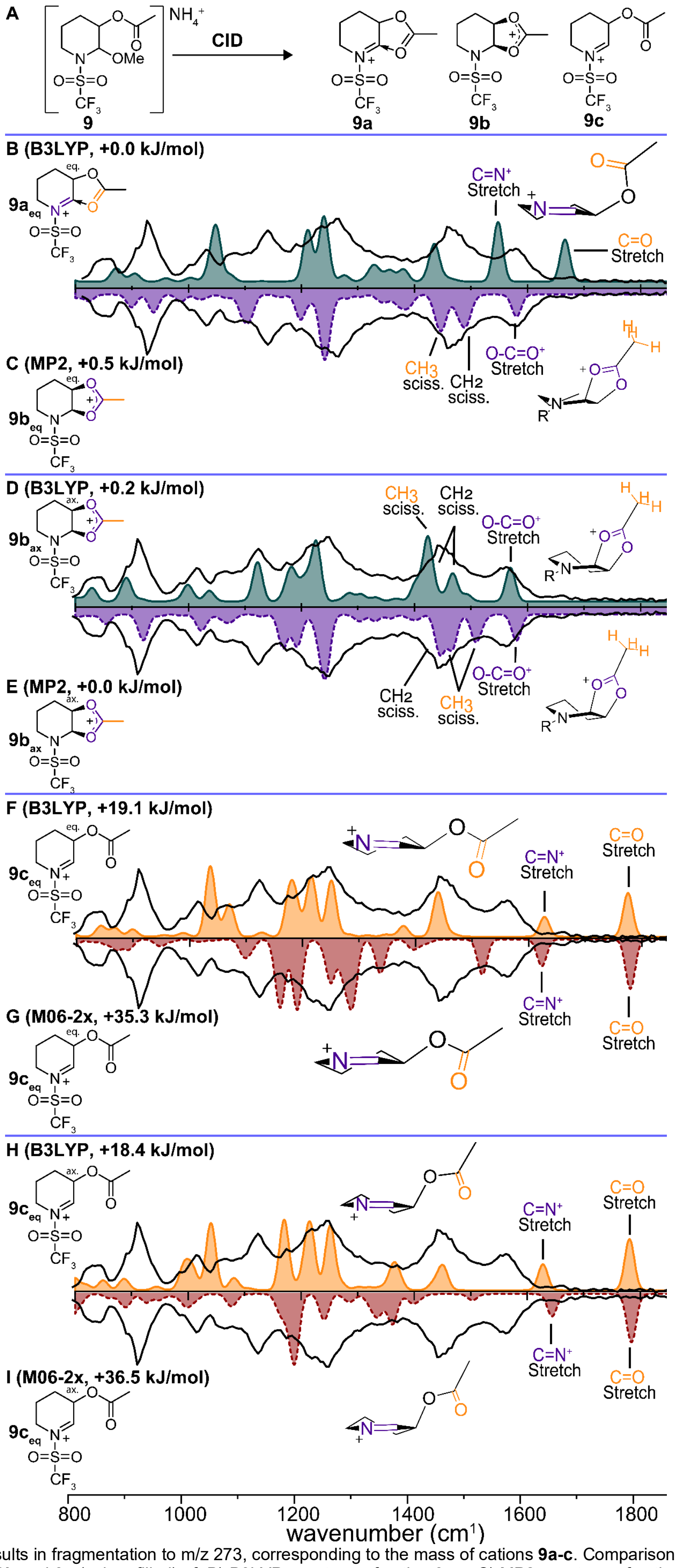

Figure S8: A) CID of precursor **9** results in fragmentation to m/z 273, corresponding to the mass of cations **9a-c**. Comparison of the spectrum m/z 273 (black line) with the DFT calculated cations **9a**, **9b** and **9c** (colour filled) of: B) B3LYP spectrum of cation $\mathbf{9a}_{\mathbf{eq}}$, C) MP2 spectrum of cation $\mathbf{9b}_{\mathbf{eq}}$ (mirrored in x-axis), D) B3LYP spectrum of cation $\mathbf{9b}_{\mathbf{ax}}$, E) MP2 spectrum of cation $\mathbf{9b}_{\mathbf{ax}}$ (mirrored in x-axis). F) B3LYP spectrum of cation $\mathbf{9c}_{\mathbf{eq}}$, G) MO6-2X spectrum of cation $\mathbf{7c}_{\mathbf{eq}}$ (mirrored in x-axis), H) B3LYP spectrum of cation $\mathbf{9c}_{\mathbf{eq}}$ and I) MO6-2X spectrum of cation $\mathbf{9c}_{\mathbf{eq}}$ (mirrored in x-axis). Energies are relative to the lowest-energy structure, carbamate omitted for clarity in the 3D schematic representation of the molecule.

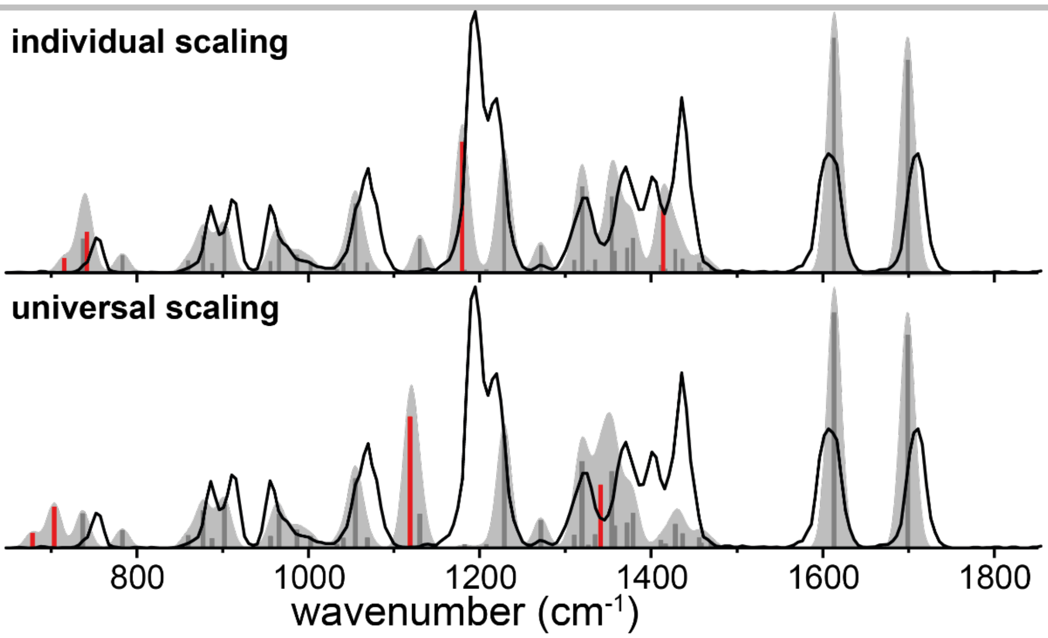

Figure S9: The measured IR ion spectrum of m/z 220 (black lines) generated from **8** compared to computational spectra of the assigned structures that employ individual scaling (top) and universal scaling (bottom). In the case of individual scaling the vibrations involving sulfur (red bars) are scaled by a factor of 1.028, as opposed to 0.975, which is used for all other vibrations.

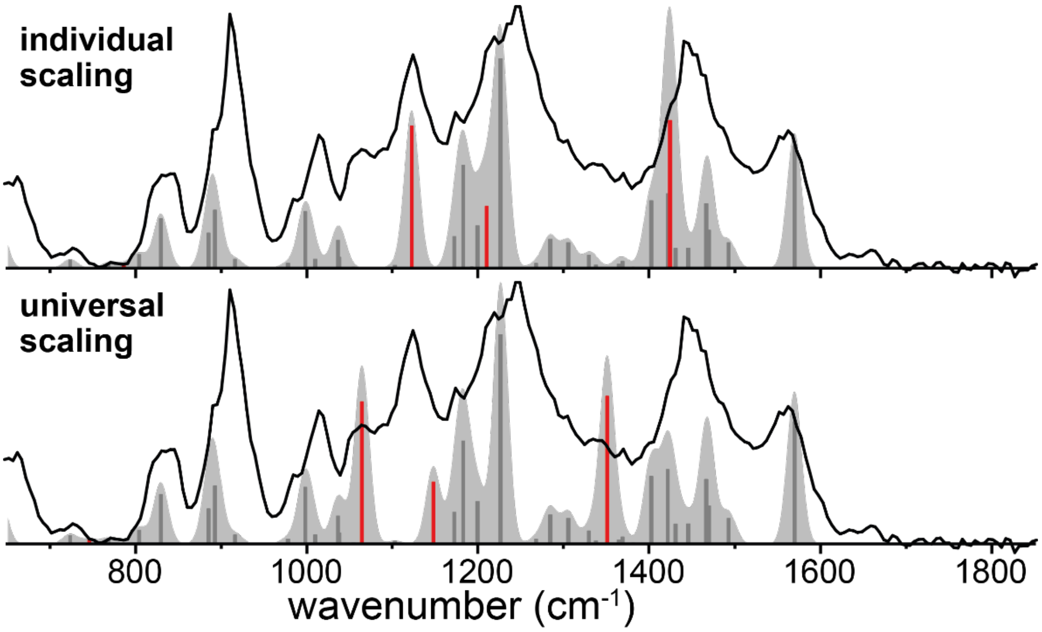

Figure S10: The measured IR ion spectrum of m/z 274 (black lines) generated from **9** compared to computational spectra of the assigned structures that employ individual scaling (top) and universal scaling (bottom). In the case of individual scaling the vibrations involving sulfur (red bars) are scaled by a factor of 1.028, as opposed to 0.975, which is used for all other vibrations.

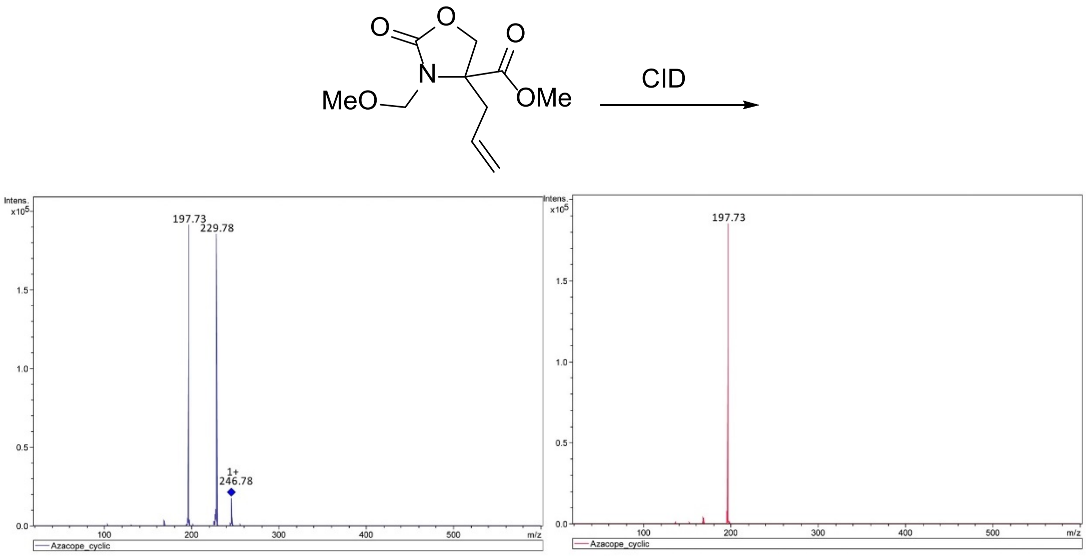

Figure S11: CID of compound **10**

General Synthetic Methods

^{1}H and ^{13}C NMR spectra were recorded on a Bruker 400 MHz or 500 MHz spectrometer. Chemical shifts are reported in parts per million (ppm) relative residual solvents as the internal standard. NMR data is presented as follows: chemical shift, multiplicity (s = singlet, d = doublet, t = triplet, dd = doublet of doublets, m = multiplet and/or multiple resonances), coupling constant in hertz (Hz), integration. All NMR signals were assigned on the basis of ^{1}H NMR, ^{13}C NMR, COSY, HSQC and HMBC experiments. Mass spectra were recorded on an JEOL AccuTOF CS JMST100CS mass spectrometer. Automatic flash column chromatography was performed using Biotage Isolera Spektra One, using SNAP cartridges (Biotage, 30-100 µm, 60 Å), 10-50 g. TLC-analysis was conducted on Silicagel F254 (Merck KGaA) with detection by UV-absorption (254nm) where applicable or by spraying with 6% sulphuric acid in water containing 5 wt% Ammonium molybdate, 0.2 wt%. DCM, THF and toluene were freshly distilled. All inert reactions were carried out under argon atmosphere using flame-dried flasks. Purification by column chromatography over SiO_2, unless otherwise specified.

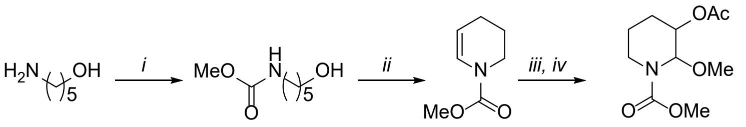

S1 **S2** **5** **7**

Scheme S1. Synthesis of precursors **5** and **7**. Reagents and conditions: i) $MeCO_2Cl$, TEA, DCM, 95%; ii) PCC/Si_2O, DCM, 45%; iii) Oxone, $NaHCO_3$, MeOH; iv) Ac_2O, DMAP, TEA, DCM, 51% over two steps.

Methyl (5-hydroxypentyl)carbamate (S2)
To a solution of 5-amino-1-pentanol (3.0 g, 29.1 mmol) in water/ethanol (1:1, 60 mL) was added solid K_2CO_3 (8.04 g, 58.2 mmol). The reaction mixture was cooled with an icebath and methyl carbonochloridate (2.97 mL, 43.6 mmol) was dropwise added. The resulting mixture was strirred for 16 hwhile allowed to warm to rt. The mixture was diluted with DCM (50 mL) and the layers were separated. The organic layer was washed with 0.1 M HCl (10 mL) and brine (10 ml), dried over Na_2SO_4 and concentrated *in vacuo*. The resulting residue was purified by column chromatography (5% MeOH in DCM) to furnish methyl (5-hydroxypentyl)carbamate as a yellow oil (4.5 g, 95%). R_f = 0.45 (MeOH/DCM, 1:9); ^{1}H NMR (500 MHz, Chloroform-*d*) δ 12.07 (s, 1H), 4.72 (s, 1H), 3.70 – 3.64 (m, 4H), 3.64 (s, 1H), 3.19 (s, 2H), 1.71 – 1.48 (m, 4H), 1.42 (t, *J* = 7.2 Hz, 2H). ^{13}C NMR (126 MHz, Chloroform-*d*) δ 62.7, 45.8, 40.9, 32.2, 28.7, 22.9; HRMS (ESI) $[M + Na]^+$ calcd. for $C_7H_{15}NNaO_3$: 184.0950, found: 184.0930.

Methyl 3,4-dihydropyridine-1(2*H*)-carboxylate (5)
PCC (6 g, 30 mmol) and silica gel (6 g) were dried for 24 h *in vacuo*, mixed and ground in a mortar. The resulting powder was suspended in anhydrous DCM (250 mL). **S2** (2.770 g, 17.18 mmol) dissolved in anhydrous DCM (20 mL) was added in one portion and the reaction mixture was sonicated until completion (~1-2 h). The mixture was concentrated *in vacuo*. The resulting residue was filtered over Celite and thoroughly washed with Et_2O and concentrated *in vacuo*. The residue was further purified by column chromatography (5% EtOAc/heptane) which allowed the isolation of methyl 3,4-dihydropyridine-1(2H)-carboxylate (1.1 g, 45%) as a yellowish oil. R_f = 0.45 (1:1 EtOAc heptane); ^{1}H NMR (500 MHz, Chloroform-*d*, rotamers) δ 6.86 (d, *J* = 8.5 Hz, 1H, minor), 6.73 (d, *J* = 8.5 Hz, 1H, major), 4.94 (m, 1H, minor), 4.85 (m, 1H, major) 3.74 (s, 3H), 3.59 (m, 2H), 2.04 (tdd, *J* = 6.1, 3.9, 2.0 Hz, 2H), 1.83 (p, *J* = 6.2 Hz, 2H). ^{13}C NMR (126 MHz, Chloroform-*d*) δ 125.5, 125.0, 106.6, 106.4, 53.0, 52.9, 42.5, 42.3, 21.8, 21.6, 21.4; HRMS (ESI) $[M + H]^+$ calcd. for $C_7H_{12}NO_2$: 142.0868, found: 142.0897.

Methyl 3-acetoxy-2-methoxypiperidine-1-carboxylate (7)
A solution of **5** (486 mg, 0.37 mmol) in MeOH (10 mL) was added dropwise to a suspension of Oxone® (1.37 g, 4.48 mmol) and $NaHCO_3$ (434 mg, 5.16 mmol) and the mixture was stirred overnight at room temperature. After removal of the solids by filtration, the remaining filtrate was diluted with DCM (30 mL) and the organic phase was washed with sat. aq. $NaHCO_3$ (15 mL), brine (15 mL), dried over Na_2SO_4 and concentrated *in vacuo*. This residue was dissolved in MeCN (5 mL), subsequently acetic anhydride (79 µl, 0.84 mmol), TEA (49.5 µl, 0.36 mmol) and DMAP (7.7 mg, 63 µmol) were added to this suspension. After completion conversion, methanol (2 µl) was added to the mixture and stirring was continued for another 5 min. The mixture was concentrated *in vacuo* and the resulting residue was purified by column chromatography (15% EtOAc/heptane) resulting in **7** as a yellow oil (320 mg, 40%). R_f=0.25 (EtOAc/Hep, 30:70); ^{1}H NMR (500 MHz, $CDCl_3$, rotamers) δ 5.23 (d, *J* = 63.9 Hz, 1H), 4.90 (d, *J* = 35.2 Hz, 1H), 4.05 – 3.83 (m, 1H), 3.75 – 3.67 (m, 3H), 3.31 – 3.19 (m, 3H), 2.97 (dt, *J* = 23.8, 13.2 Hz, 1H), 2.03 (s, 3H), 1.98 – 1.86 (m, 1H), 1.73 (d, *J* = 14.5 Hz, 2H), 1.43 (t, *J* = 11.6 Hz, 1H); ^{13}C NMR (126 MHz, $CDCl_3$, rotamers) δ 170.3, 82.5, 68.3, 68.0, 55.2, 54.8, 52.8, 38.5, 38.0, 29.8, 23.5, 21.2, 19.6, 19.4; HRMS (ESI) $[M + Na]^+$ calcd. for $C_{10}H_{17}NNaO_5$: 254.1004, found: 254.0985.

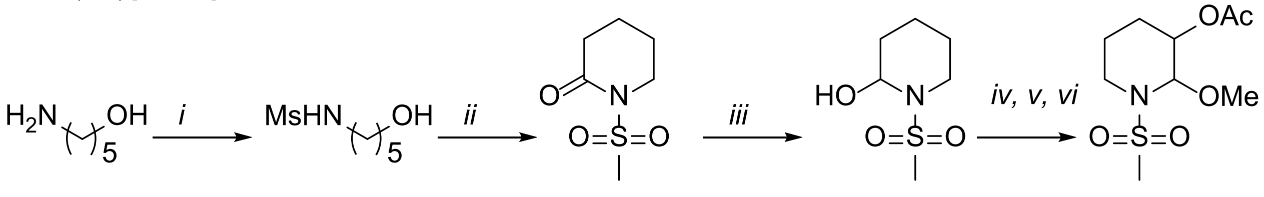

S1 **S3** **S4** **S5** **8**

Scheme S2. Synthesis of precursor **8**. Reagents and conditions: i) $(MeSO_2)_2O$, TEA, MeCN, 75%; ii) PCC/Si_2O, DCM, 59%; iii) DiBAL-H, THF, 52%, iv) mCPBA, $NaHCO_3$, MeOH; iv) Ac_2O, DMAP, TEA, DCM, 51% over two steps.

N-(5-Hydroxypentyl)methanesulfonamide (S3)
A solution of 5-aminopentan-1-ol (1.0 g, 9.7 mmol) in MeCN (50 mL) was cooled to 0º C, subsequently TEA (1.6 ml, 12 mmol) and Ms_2O (1.7g, 9.7 mmol) were added. After complete conversion, the solution was concentrated *in vacuo*. The resulting residue was purified by column chromatography (80%->100% EtOAc/ heptane) resulting in 1.32 g of a viscous oil (75 %, 7.3 mmol). R_f = 0.5 (1:9 MeOH:DCM); ^{1}H NMR (400 MHz, $CDCl_3$) δ 4.26 (dd, *J* = 13.5, 7.2 Hz, 1H), 3.67 (t, *J* = 6.3 Hz, 2H), 3.16 (td, *J* = 7.0, 6.2 Hz, 2H), 2.96 (s, 3H), 1.67 – 1.54 (m, 6H), 1.51 – 1.41 (m, 2H), 1.34 (s, 1H); ^{13}C NMR (101 MHz, $CDCl_3$) δ 69.7, 43.1, 40.6, 37.6, 29.7, 28.7, 22.6; HRMS (ESI) $[M + Na]^+$ calcd. for $C_6H_{15}NNaO_3S$: 204.0670, found: 204.0660.

1-(Methylsulfonyl)piperidin-2-one (S4)
A solution of **S3** (1.0 g, 5.6 mmol) was dissolved in DCM (60 mL), silica (2 g) and PCC (2.4g, 11.2 mmol) were added. The quickly black turning reaction was stirred overnight, subsequently Celite (10g) was added, and allowed to stand for 15 mins. The residue was filtered over Celite, washed with copious amounts of DCM. The solvent was removed *in vacuo*, the residue was purified by column chromatography (0->100% Et_2O/pentane) resulting in isolation of 580 mg **S5** (59%, 3.3 mmol) contaminated with an unknown impurity. R_f = 0.18 (Et_2O); ^{1}H NMR (400 MHz, $CDCl_3$) δ 3.81 – 3.71 (m, 2H), 3.33 (s, 3H), 2.61 – 2.46 (m, 2H), 1.93 – 1.78 (m, 4H); ^{13}C NMR (101 MHz, $CDCl_3$) δ 171.8, 46.2, 34.2, 23.2, 20.5.

1-(Methylsulfonyl)piperidin-2-ol (S5)
A solution of **S4** (391 mg, 2.21 mmol) in THF (12 mL) was cooled to -78ºC, DIBAL-H (2.4 mL, 1.0 M, 2.4 mmol) was dropwise added. After 2 h, the reaction was quenched with addition of sat. aq. potassium sodium tartrate and further diluted withDCM (10 mL). After separation of the layers, the aqueous phase was extracted with DCM (2x 5 mL), the combined organic phases were washed with brine (5 mL), dried over $MgSO_4$ and concentrated *in vacuo*. Purification by column chromatography (20->100% Et_2O/pentane) yielded 205 mg of **S5** (52%, 1.15 mmol) with recovery of 40 mg of the starting material **S4** (10%, 0.23 mmol). R_f = 0.32 (Et_2O); NMR ^{1}H NMR (400 MHz, $CDCl_3$) δ 5.54 (dt, *J* = 5.0, 2.6 Hz, 1H), 3.58 – 3.49 (m, 1H), 3.18 (td, *J* = 12.0, 2.8 Hz, 1H), 2.92 (s, 3H), 2.44 (d, *J* = 4.5 Hz, 1H), 2.00 – 1.49 (m, 6H); ^{13}C NMR (101 MHz, $CDCl_3$) δ 67.8, 40.5, 39.4, 32.1, 25.1, 17.3, HRMS (ESI) $[M + Na]^+$ calcd. for $C_6H_{13}NNaO_3S$: 202.0514, found: 202.0450.

2-Methoxy-1-(methylsulfonyl)piperidin-3-yl acetate (8)
A microwave vial equipped with a stirring bar was charged with a solution of **S5** (98 mg, 0.54 mmol) and *p*-TsOH (1 mg, 0.05 mmol) in DCE (1.5 mL). The microwave was set to 160 ºC, and the solution was kept at this temperature for 2 h. The solution was diluted with DCM (5 mL), washed with sat. aq. $NaHCO_3$ and the organic layer was dried over $MgSO_4$ resulting in a 88 mg of a crude product which was used without further purification. Rf 0.36 (Pentane:Et_2O, 1:1). The crude product was dissolved in MeOH (3 mL), mCPBA (113 mg, 0.65 mmol) was added in portions at 0º C. The solution was left standing at this temperature for 2 h, subsequently quenched with sat. aq. $NaHCO_3$ and extracted with DCM (3 x 5 mL). The combined organic layers were washed with water (3 mL), brine (3 mL), dried over $MgSO_4$ and concentrated *in vacuo*. Without any further purification the residue was taken up in DCM (4 mL), TEA (114 µL, 0.82 mmol) and acetic anhydride (62 µL, 0.65 mmol) were added. The solution was stirred overnight, diluted with DCM (6 mL), washed wit sat. aq $NaHCO_3$ (5 mL), dried over $MgSO_4$ and concentrated *in vacuo*. The resulting residue was purified by column chromatography (0%->30% EtOAc/ heptane) to yield 46 mg of an oil (34%, 0.18 mmol). R_f = 0.52 (Et_2O); ^{1}H NMR (400 MHz, $CDCl_3$) δ 4.98 (q, *J* = 2.6 Hz, 1H), 4.90 (dd, *J* = 2.6, 1.0 Hz, 1H), 3.64 – 3.55 (m, 1H), 3.39 (s, 3H), 3.15 (td, *J* = 12.9, 2.8 Hz, 1H), 2.95 (s, 3H), 2.08 (s, 3H), 1.98 – 1.75 (m, 3H), 1.53 (dddt, *J* = 11.9, 5.9, 3.9, 2.0 Hz, 1H); ^{13}C NMR (101 MHz, $CDCl_3$) δ 170.0, 84.5, 67.2, 55.7, 40.4, 40.1, 23.0, 21.3, 19.4; HRMS (ESI) $[M + Na]^+$ calcd. for $C_9H_{17}NNaO_5S$: 274.0725, found: 274.0709.

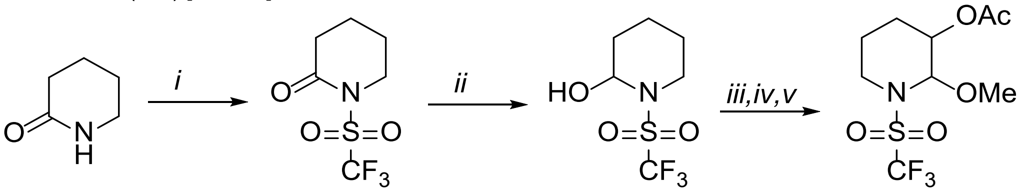

Scheme S3. Synthesis of precursor **9**. Reagents and conditions: i) Tf_2O, TEA, DCM, 97%; ii) DiBAL-H, THF 60%; iii) pTsOH.H_2O, toluene, iv) mCPBA, $NaHCO_3$, MeOH; v) Ac_2O, DMAP, TEA, DCM, 44% over three steps.

1-((Trifluoromethyl)sulfonyl)piperidin-2-one (S7)
A solution of 2-pyrrolidinone (2.0 g, 20 mmol) and TEA (3.4 mL, 24 mmol) in DCM (70 mL) was cooled to -78 ºC, to this solution was added Tf_2O (3.4 mL, 20 mmol) and the resulting red solution was stirred for 4h. The reaction mixture was concentrated *in vacuo*. The resulting residue was purified by column chromatography (5%->35% EtOAc/ heptane) to yield 4.5 g of an colorless oil (97%, 19.5 mmol). R_f = 0.65 (hept:EtOAc = 1:1); ^{1}H NMR (400 MHz, $CDCl_3$) δ 3.89 (dd, *J* = 6.7, 4.8 Hz, 2H), 2.82 – 2.60 (m, 2H), 2.01 – 1.87 (m, 4H); ^{13}C NMR (101 MHz, $CDCl_3$) δ 170.4, 119.7 (q, *J* = 325 hz), 49.3, 34.6, 23.2, 20.3.

1-((Trifluoromethyl)sulfonyl)piperidin-2-ol (S8)
A solution of **S8** (4.1 g, 17.6 mmol) in THF (75 mL) was cooled to -78 ºC, to this solution was added DiBAL-H (20.2 mL, 20.2 mmol, 1M in hexanes) and the resulting solution was stirred for 1 h and allowed to warm to -20 ºC. The reaction was quenched by addition of sat. aq. potassium sodium tartrate (10 mL) and vigorously stirred overnight. The resulting mixture was diluted with DCM (25 mL), the layers were separated. The aqueous phase was extracted with DCM (3x 25 mL), the combined organic phases were washed with brine (5 mL), dried over $MgSO_4$ and concentrated *in vacuo*. The resulting residue was purified by column chromatography (5%->35% EtOAc/ heptane) to yield 2.4 g of an colorless oil (60%, 10.3 mmol).). R_f = 0.75 (hept:EtOAc = 1:1); ^{1}H NMR (400 MHz, $CDCl_3$) δ 5.57 (q, *J* = 3.3 Hz, 1H), 3.85 – 3.66 (m, 1H), 3.49 (tdd, *J* = 12.9, 2.8, 1.2 Hz, 1H), 2.67 (d, *J* = 7.7 Hz, 1H), 2.01 – 1.93 (m, 1H), 1.89 – 1.76 (m, 2H), 1.74 – 1.51 (m, 3H); ^{13}C NMR (101 MHz, $CDCl_3$) δ 119.3 (q, *J* = 325 hz), 78.1, 42.2, 31.4, 25.1, 17.0.

2-Methoxy-1-((trifluoromethyl)sulfonyl)piperidin-3-yl acetate (9)

A microwave vial equipped with a stirring bar was charged with a solution of **S8** (1.95 g, 8.4 mmol) and *p*-TsOH.H_2O (16 mg, 0.08 mmol) in Toluene (2 mL). The microwave was set to 160 °C, and the solution was kept at this temperature for 3 h. The solution was diluted with DCM (20 mL), washed with sat. aq. $NaHCO_3$ (5 mL) and the organic layer was dried over $MgSO_4$. The resulting residue was taken up in MeOH (30 mL) and was subsequently cooled with an ice bath. To this solution was added mCPBA (1.74 g, 10.11 mmol) and the reaction was left stirring vigorously overnight while allowed to warm up to rt. The reaction was quenched by the addition of sat. aq. $NaHCO_3$ (10 mL) and further diluted by addition of EtOAc (30 mL). The layers were separated, the aqueous phase was extracted with EtOAc (3x 10 mL), the combined organic phases were washed with brine (10 mL), dried over $MgSO_4$ and concentrated *in vacuo*. The resulting residue was taken up in DCM (40 mL) followed by the addition of TEA (1.76 mL, 12.64 mmol), Ac_2O (0.95 ml, 10.1 mmol) and DMAP (103 mg, 0.84 mmol). The resulting solution was left standing overnight. The reaction was quenched by the addition of sat. aq. $NaHCO_3$ (10 mL) and further diluted by addition of DCM (30 mL). The layers were separated, the aqueous phase was extracted with EtOAc (3x 10 mL), the combined organic phases were washed with brine (10 mL), dried over $MgSO_4$ and concentrated *in vacuo*. Purification by column chromatography (5%->35% EtOAc/ heptane) yielded 1.1 g of **9** as an oil (44%, 3.7 mmol over 3 steps). R_f = 0.67 (hept:EtOAc = 1:1); ^{1}H NMR (500 MHz, $CDCl_3$) δ 4.94 – 4.90 (m, 1H), 4.89 – 4.84 (m, 1H), 3.72 – 3.64 (m, 1H), 3.38 (s, 3H), 3.29 (td, *J* = 13.2, 2.6 Hz, 1H), 2.05 (s, 3H), 2.01 – 1.85 (m, 2H), 1.82 – 1.75 (m, 1H), 1.58 – 1.49 (m, 1H); ^{13}C NMR (126 MHz, $CDCl_3$) δ 170.1, 119.8 (q, *J* = 322 hz), 85.6, 67.3, 55.9, 42.0, 22.7, 20.8, 19.3.

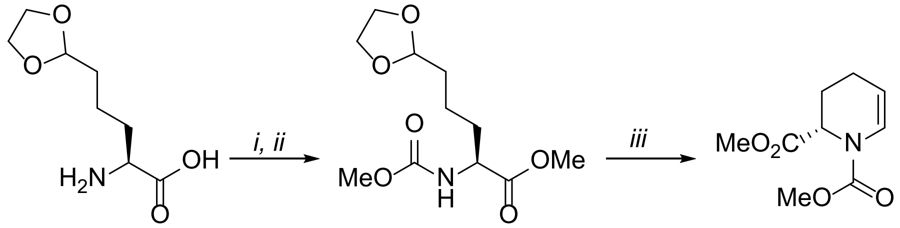

S9 **S10** **6**

Scheme S4. Synthesis of precursor **6**. Reagents and conditions: i) $MeCO_2Cl$, NaOH, $NaHCO_3$, dioxane, H_2O; ii) MeI, K_2CO_3, DMF, 44% over two steps; iii) pTsOH, DMF, toluene, 62%.

Methyl (*S*)-5-(1,3-dioxolan-2-yl)-2-((methoxycarbonyl)amino)pentanoate (S10)

L-Allysine ethylene acetal (**S9**) (1.0 g, 5.29 mmol) was suspended in dioxane (5 mL) and cooled to 0 °C. A solution of 2N NaOH (2.7 mL) was slowly added, followed by $NaHCO_3$ (0.4 g, 5 mmol) and methyl carbonochloridate (0.4 mL, 5 mmol). After stirring overnight, the solvent was removed under *in vacuo* and a solution of 2.5% $NaHCO_3$ (25 mL) was added. The unreacted methyl carbonochloridate was extracted with Et_2O (3 x 10 mL). The aqueous solution was acidified by dropwise addition of 6M HCl (2.25 mL) to pH = 3 and the product was extracted with EtOAc (3 x 15 mL). The combined organic layers were washed with brine (10 mL), dried over Na_2SO_4 and concentrated in vacuo, which was used in the next step without further purification. Rf=0.63 (MeOH/DCM, 10:90). To a solution of the crude material (0.67 g, 2.71 mmol) in dry DMF (5 mL) was added portionwise K_2CO_3 (0.316 g, 2.29 mmol) at 0 °C and the suspension was stirred vigorously at that temperature for 10 min. Then MeI (0.2 mL, 3 mmol) was added dropwise and stirring was continued for 2 h at room temperature. The reaction mixture was quenched by addition of water (5 mL) and the aqueous layer was extracted with EtOAc (3 x 5 mL). The combined organic layers were washed with aqueous $Na_2S_2O_5$ (2.5 wt%, 2 mL), brine (2 mL), dried over Na_2SO_4 and concentrated in vacuo. Purification by column chromatography (hept:EtOAc = 2:1) yielded **S10** (615 mg, 44% over two steps) as a slightly yellow oil. R_f=0.26 (EtOAc/Hep, 1:1); ^{1}H NMR (500 MHz, Chloroform-*d*) δ 5.25 (d, *J* = 8.4 Hz, 1H), 4.84 (t, 1H), 4.40 – 4.32 (m, 1H), 4.00 – 3.90 (m, 2H), 3.89 – 3.78 (m, 2H), 3.75 (s, 3H), 3.68 (s, 3H), 1.93 – 1.83 (m, 1H), 1.77 – 1.62 (m, 2H), 1.55 – 1.41 (m, 2H). ^{13}C NMR (126 MHz, Chloroform-*d*) δ 172.96, 156.52, 104.06, 64.87, 53.79, 52.35, 33.22, 32.42, 19.69; HRMS (ESI) $[M + Na]^+$ calcd. for $C_{11}H_{19}NNaO_4$ 284.1110, found 284.1126.

Dimethyl (S)-3,4-dihydropyridine-1,2(2*H*)-dicarboxylate (6)

To a solution of **S10** (600 mg, 2.30 mmol) in toluene (6 mL) were added anhydrous DMF (90 µL, 1.15 mmol) and pTsOH.H_2O (43.7 mg, 0.23 mmol) and the reaction was stirred at 115 °C for 2 h. After cooling to room temperature the mixture was diluted with EtOAc (10 mL), washed with an aqueous saturated $NaHCO_3$ solution (5 mL), dried over Na_2SO_4 and concentrated *in vacuo*. Purification by column chromatography (heptane:EtOAc = 5:1→3:1) yielded **6** (282 mg, 62 %) as a colorless oil. R_f =0.86 (EtOAc/Hep, 1:1); ^{1}H NMR (400 MHz, Chloroform-*d*, rotamers) δ 6.78 (d, J = 8.4 Hz, 1H), 4.98 – 4.91 (m, 1H), 4.88 – 4.79 (m, 1H), 3.78 (s, 3H), 3.73 (d, J = 5.6 Hz, 7H), 2.40 – 2.27 (m, 2H), 2.04 (s, 1H), 2.04 – 1.82 (m, 2H). ^{13}C NMR (101 MHz, Chloroform-*d*, rotamers) δ 171.6, 171.4, 154.1, 153.9, 124.7, 124.3, 105.6, 105.4, 54.1, 53.8, 53.34, 53.27, 52.5, 23.7, 23.5, 18.6, 18.4; HRMS (ESI) $[M + Na]^+$ calcd. for $C_9H_{13}NNaO_4$: 222.07423, found: 222.07346.

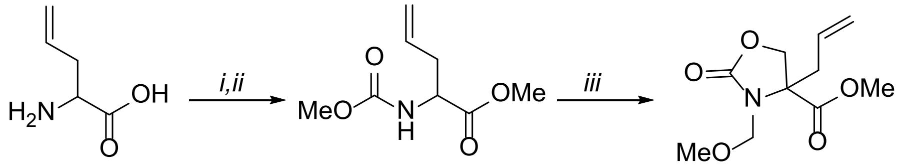

S12 **S13** **10**

Scheme S5. Synthesis of. Reagents and conditions: i) $MeCO_2Cl$, $NaHCO_3$, H_2O; ii) MeI, K_2CO_3, DMF, 83% over two steps; iii) MOMCl, NaH, DMF, 40%.

Methyl 2-((methoxycarbonyl)amino)pent-4-enoate (S12)

To an ice-cold mixture of allylglycine (400 mg, 3.47 mmol) and $NaHCO_3$ (876 mg, 10.43 mmol) in H_2O (16 mL) was added a solution of methyl chloroformate (403 µL, 5.21 mmol) in Et_2O (1.6 mL) and the resulting mixture was allowed to warm to rt. After stirring for 16

h, the mixture was acidified to pH < 3, extracted with EtOAc, dried over $MgSO_4$ and concentrated *in vacuo*. The resulting residue was taken up in DMF (16 mL) and cooled to 0 °C with an ice bath. K_2CO_3 (576 mg, 4.17 mmol) was added in one portion and the mixture was stirred for 15 min. MeI (325 µL, 5.21 mmol) was added and the mixture was allowed to warm to rt and was stirred for 16 h. The reaction mixture was quenched with the addition of 10% $Na_2S_2O_5$ (15 mL), EtOAc (50 mL) was added and the layers were separated. The organic layer was washed with water (3 x 50 mL), brine (10 mL), dried over $MgSO_4$ and concentrated *in vacuo*. The resulting residue was purified by column chromatography (heptane:EtOAc = 5:1→3:1) yielding **S12** (540 mg, 83 %) as a colorless oil. Rf 0.37 (EtOAc/Hep, 4:6); ^{1}H NMR (500 MHz, $CDCl_3$) δ 5.75 – 5.63 (m, 1H), 5.24 – 5.17 (m, 1H), 5.15 (t, *J* = 1.4 Hz, 1H), 5.13 – 5.10 (m, 1H), 4.44 (q, *J* = 6.6 Hz, 1H), 3.74 (s, 3H), 3.68 (s, 3H), 2.53 (ddt, *J* = 27.7, 14.0, 7.1 Hz, 2H); ^{13}C NMR (126 MHz, $CDCl_3$) δ 172.4, 156.4, 131.9, 119.4, 53.4, 52.51, 52.49, 36.9; HRMS [M + Na]$^+$ calcd. for $C_8H_{13}NNaO_4$: 210.0740, found 210.0764.

Methyl 2-allyl-1-(methoxymethyl)-5-oxopyrrolidine-2-carboxylate (10)

A solution of **S12** (77.3 mg, 0.413 mmol) in DMF (4 mL) was cooled to 0 °C with an ice bath, NaH (33.0 mg, 0.83 mmol, 60wt%) was added in one portion. After 30 min at this temperature, Mom-Cl (63 µL, 0.83 mmol) was added and the resulting reddish solution was allowed to warm up to rt and stirred overnight. The reaction was cooled to 0 °C, and another portion of both NaH (33.0 mg, 0.83 mmol, 60wt%) and Mom-Cl (63 µL, 0.83 mmol) were added. After stirring overnight at rt, the mixture was diluted with EtOAc (5 mL). Water (50 mL) was added followed by sepration of the layers. The organic layer was washed with water (3 x 50 mL), brine (10 mL), dried over $MgSO_4$ and concentrated *in vacuo*. The resulting residue was purified by column chromatography (heptane:EtOAc = 5:1) yielding **9** (38 mg, 40 %) as a colorless oil. Rf 0.35 (EtOAc/Hep, 4:6); ^{1}H NMR (500 MHz, $CDCl_3$) δ 5.76 – 5.65 (m, 1H), 5.28 – 5.21 (m, 2H), 4.83 (d, *J* = 11.5 Hz, 1H), 4.80 (d, *J* = 11.5 Hz, 1H), 4.51 (d, *J* = 9.0 Hz, 1H), 4.19 (d, *J* = 9.1 Hz, 1H), 3.80 (s, 3H), 3.36 (s, 3H), 2.77 (ddt, *J* = 14.6, 7.5, 1.1 Hz, 1H), 2.69 (ddt, *J* = 14.6, 7.0, 1.3 Hz, 1H); ^{13}C NMR (126 MHz, $CDCl_3$) δ 171.5, 157.8, 130.1, 121.7, 74.4, 68.8, 65.1, 56.8, 53.2, 38.3; HRMS (ESI) [M + Na]$^+$ calcd. for $C_{10}H_{15}NNaO_5$: 252.0848, found: 252.0835.

^{1}H- and ^{13}C-NMR Spectra of Compounds

5 (^{1}H, 400 MHz, $CDCl_3$)

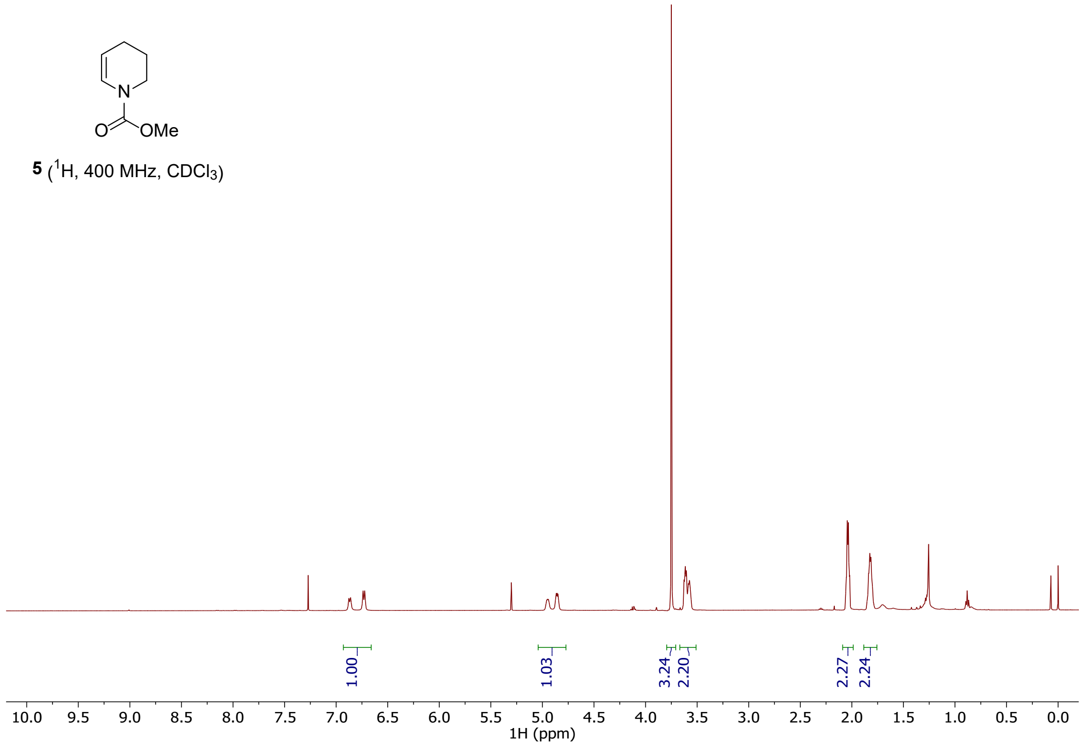

5 (^{13}C, 101 MHz, $CDCl_3$)

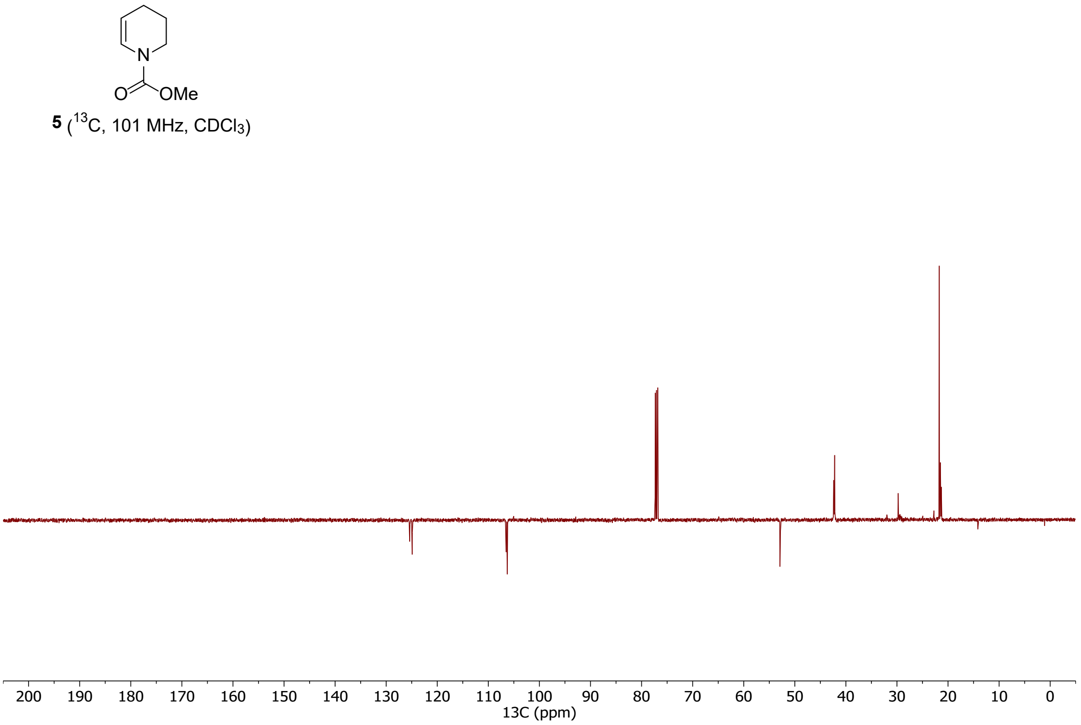

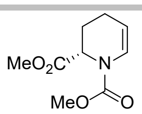

6 (^{1}H, 400 MHz, CDCl$_3$)

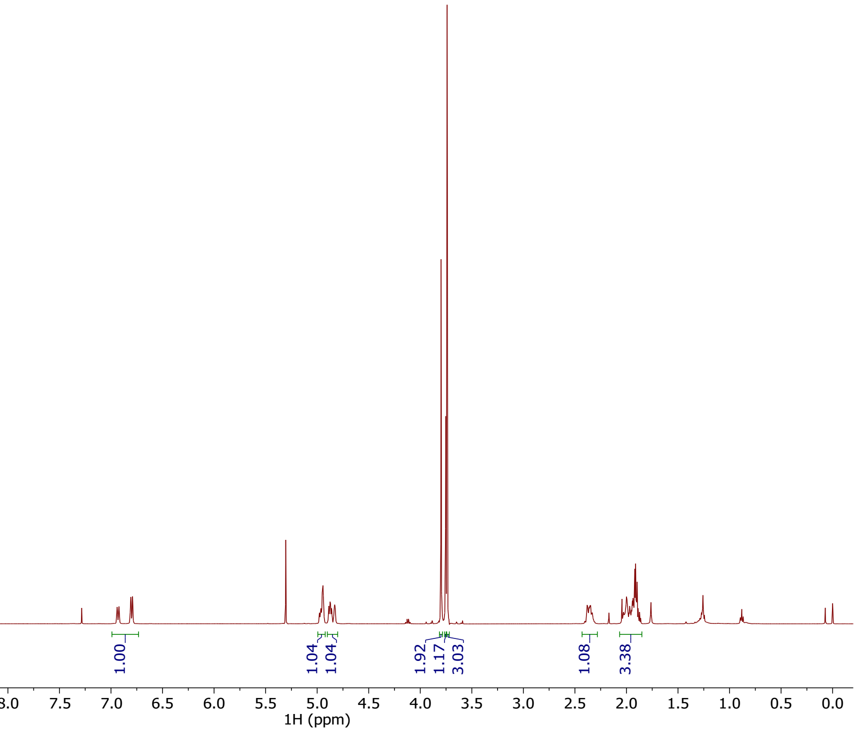

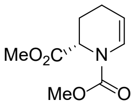

6 (^{13}C, 101 MHz, CDCl$_3$)

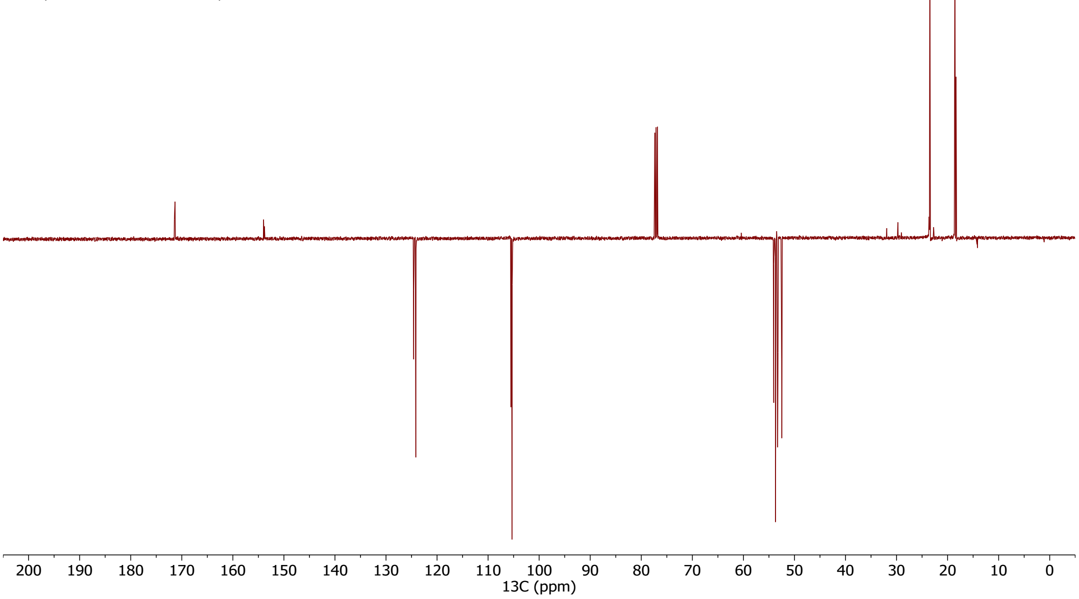

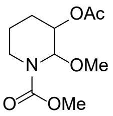

7 (^{1}H, 500 MHz, $CDCl_3$)

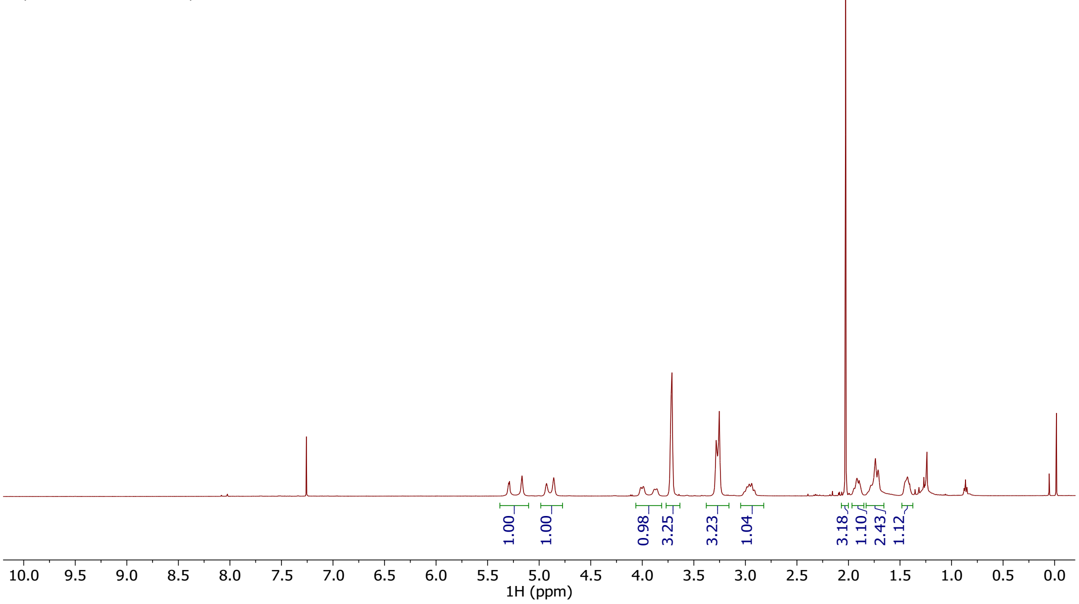

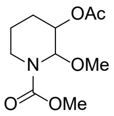

7 (^{13}C, 126 MHz, $CDCl_3$)

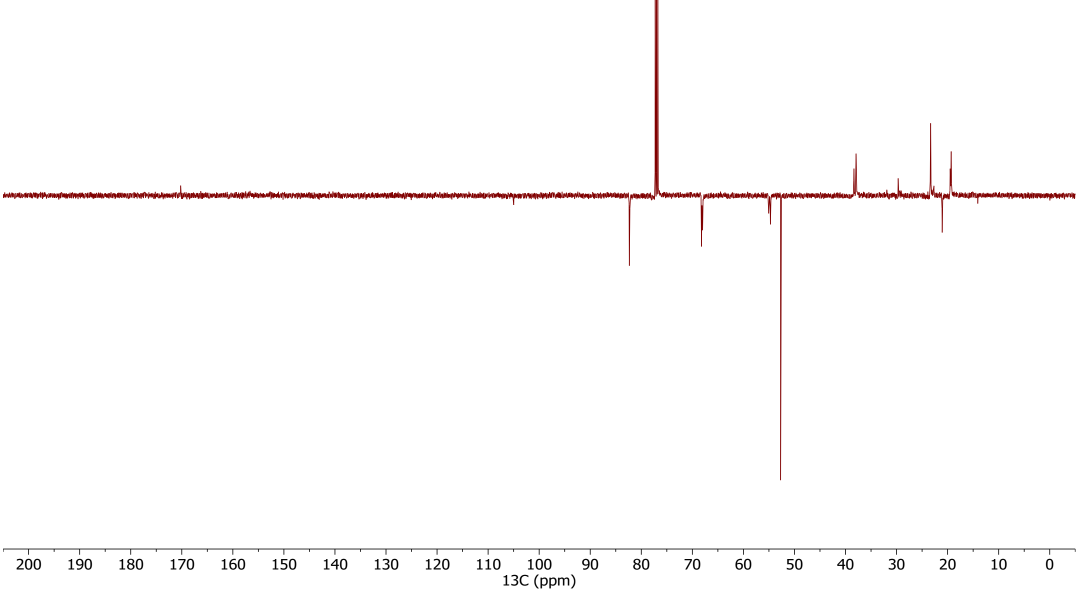

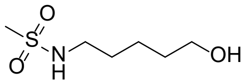

S3 (^{1}H, 400 MHz, $CDCl_3$)

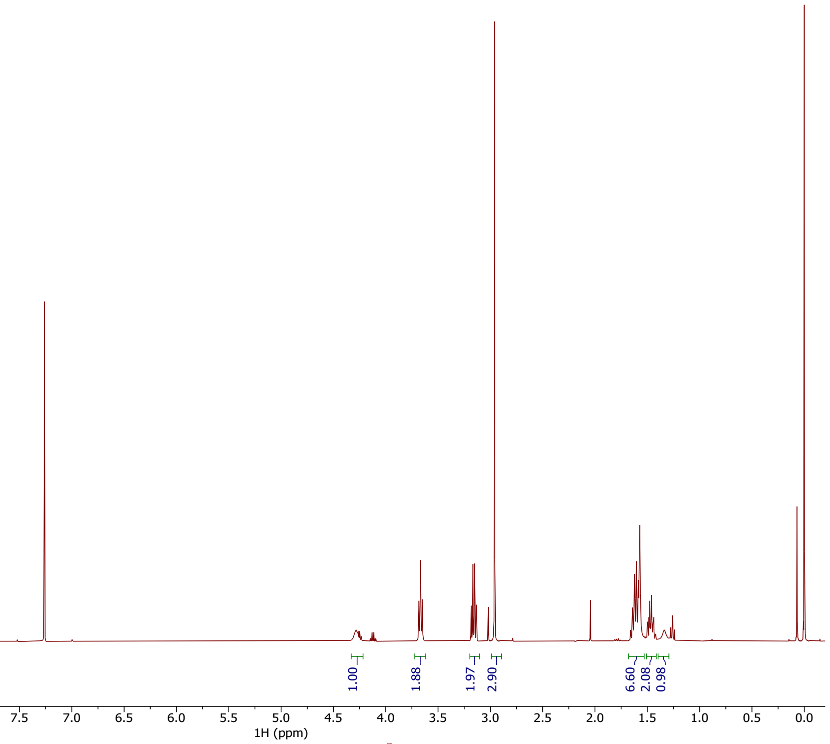

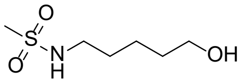

S3 (^{13}C, 101 MHz, $CDCl_3$)

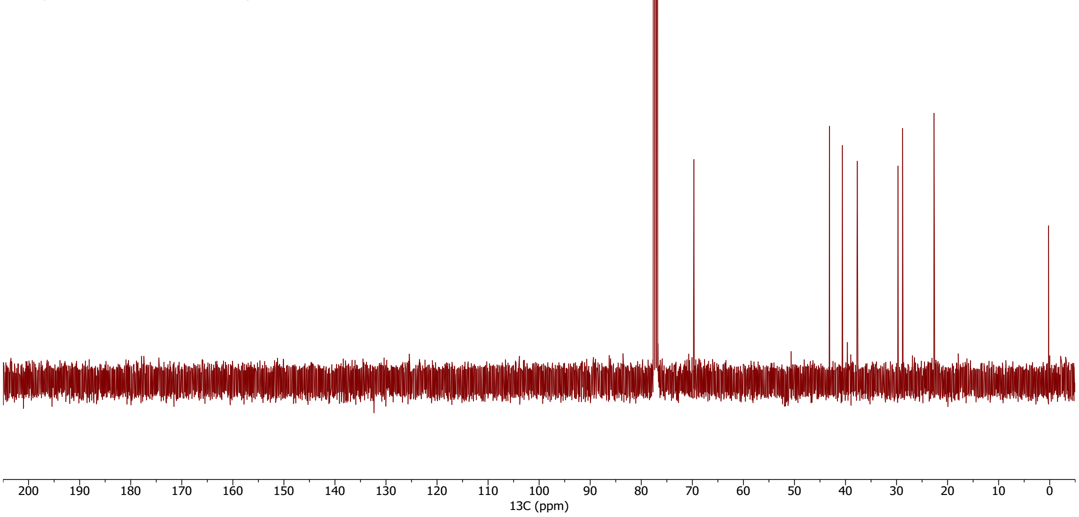

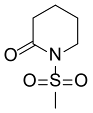

S4 (^1H, 400 MHz, $CDCl_3$)

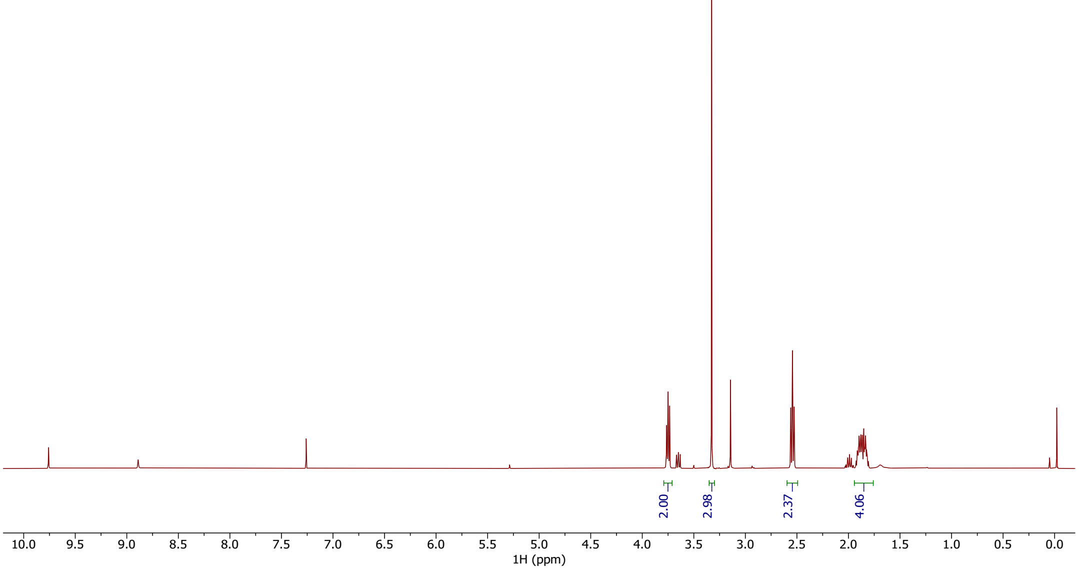

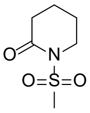

S4 (^{13}C, 101 MHz, $CDCl_3$)

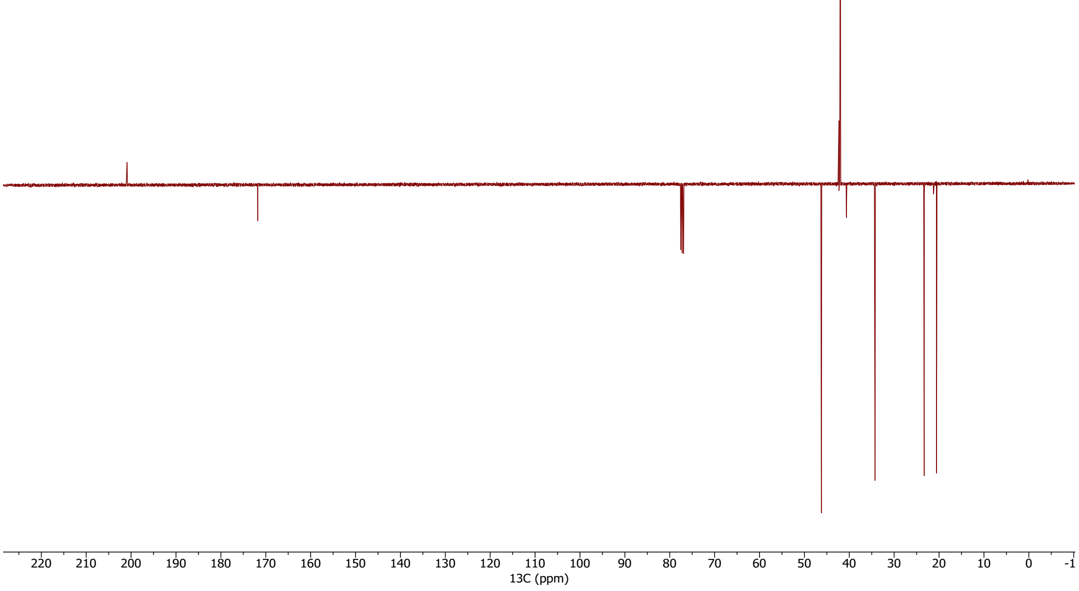

S5 (^{1}H, 400 MHz, $CDCl_3$)

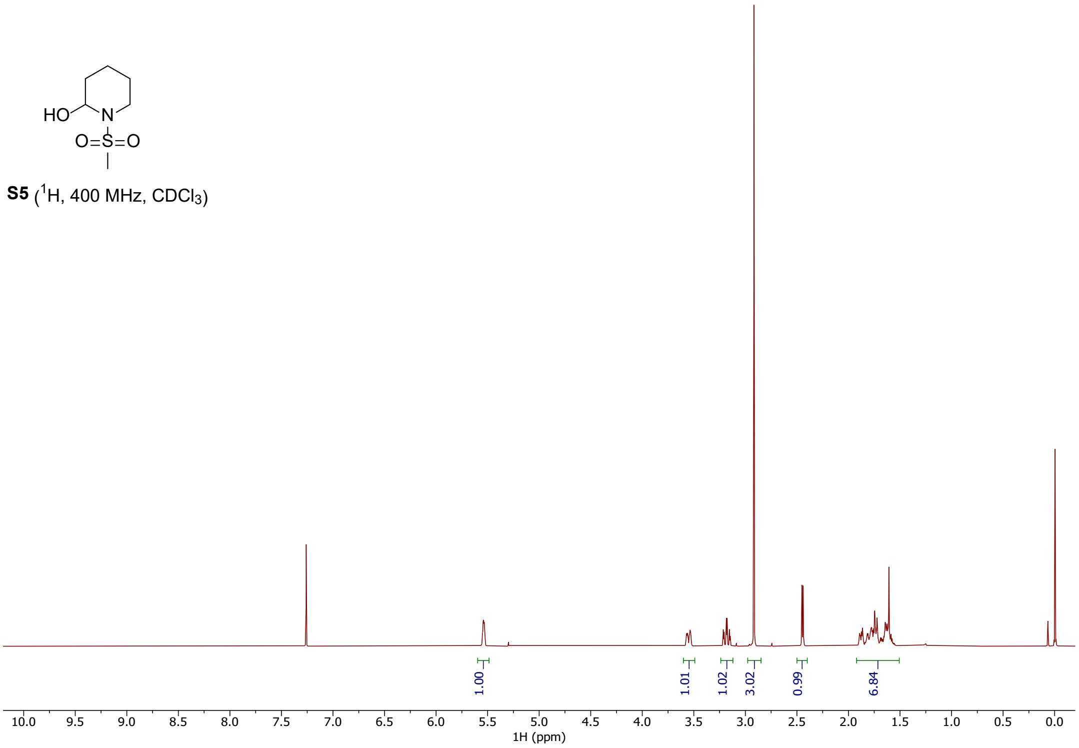

S5 (^{13}C, 126 MHz, $CDCl_3$)

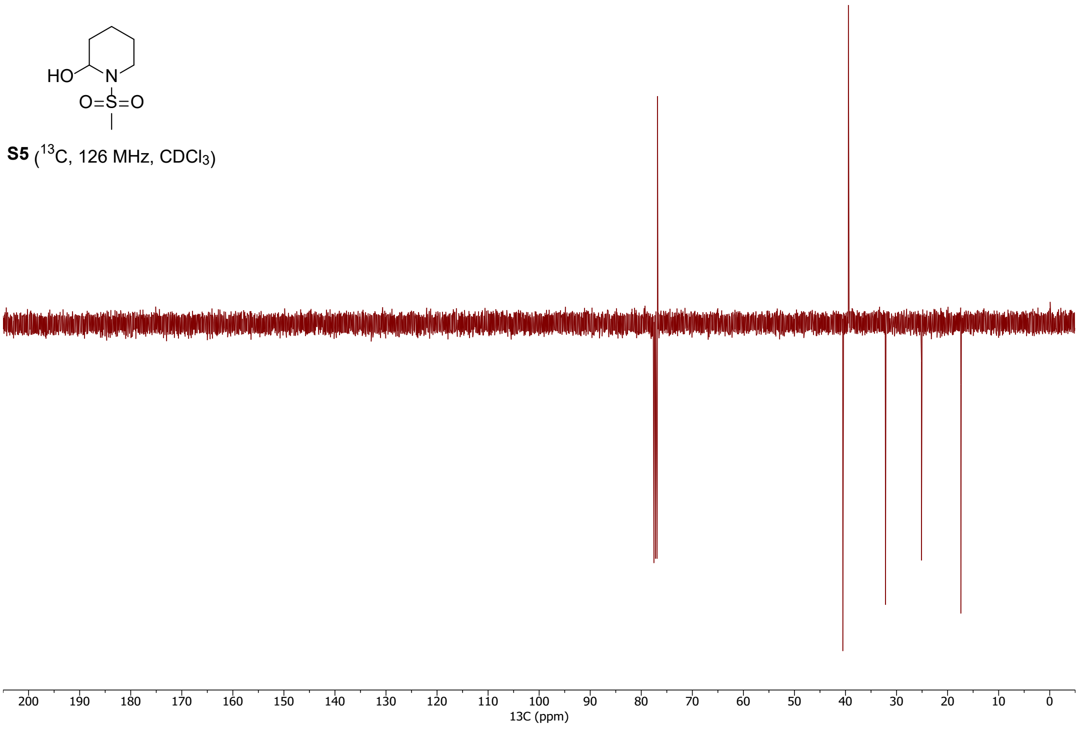

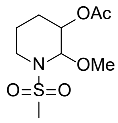

8 (^{1}H, 500 MHz, $CDCl_3$)

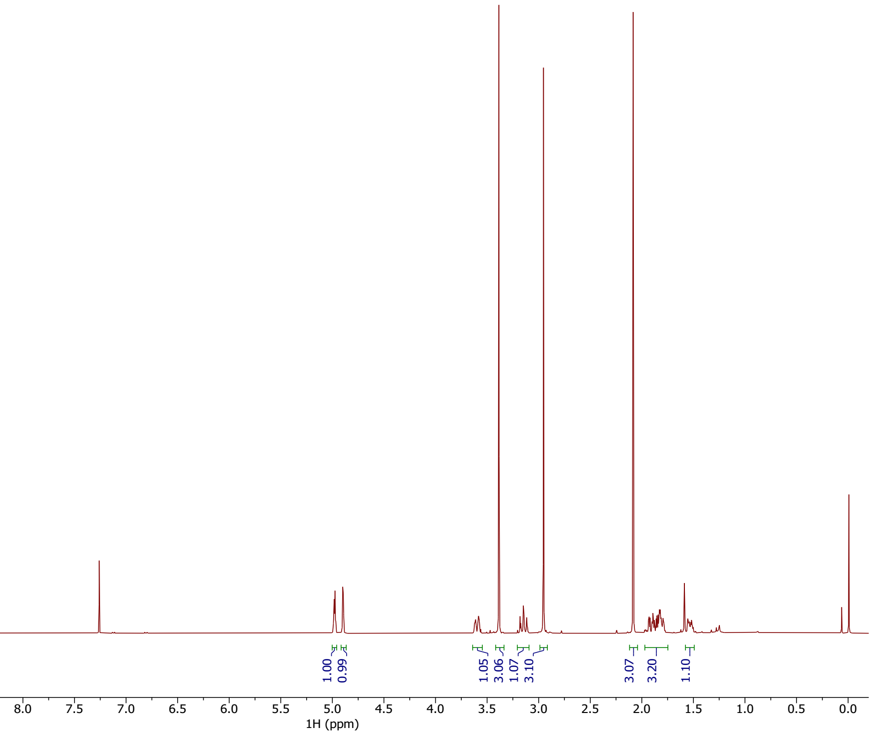

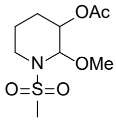

8 (^{13}C, 126 MHz, $CDCl_3$)

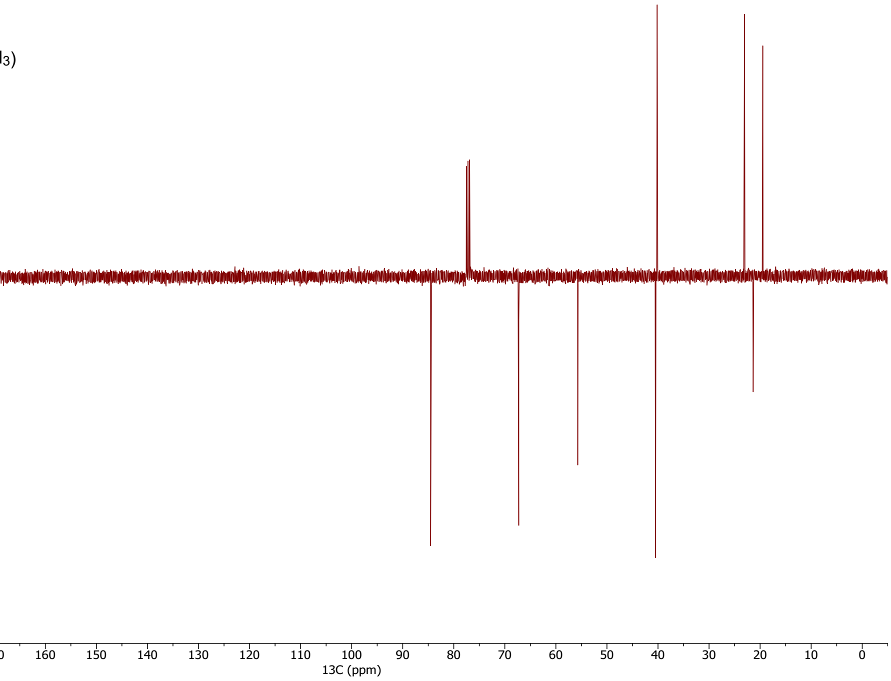

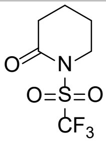

S7 (^{1}H, 400 MHz, $CDCl_3$)

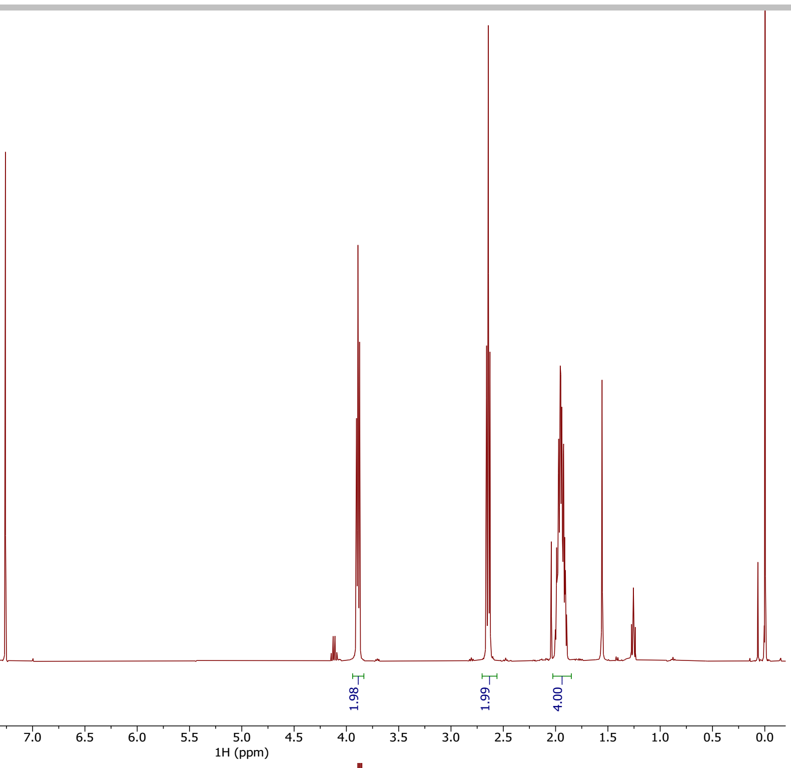

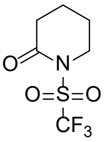

S7 (^{13}C, 101 MHz, $CDCl_3$)

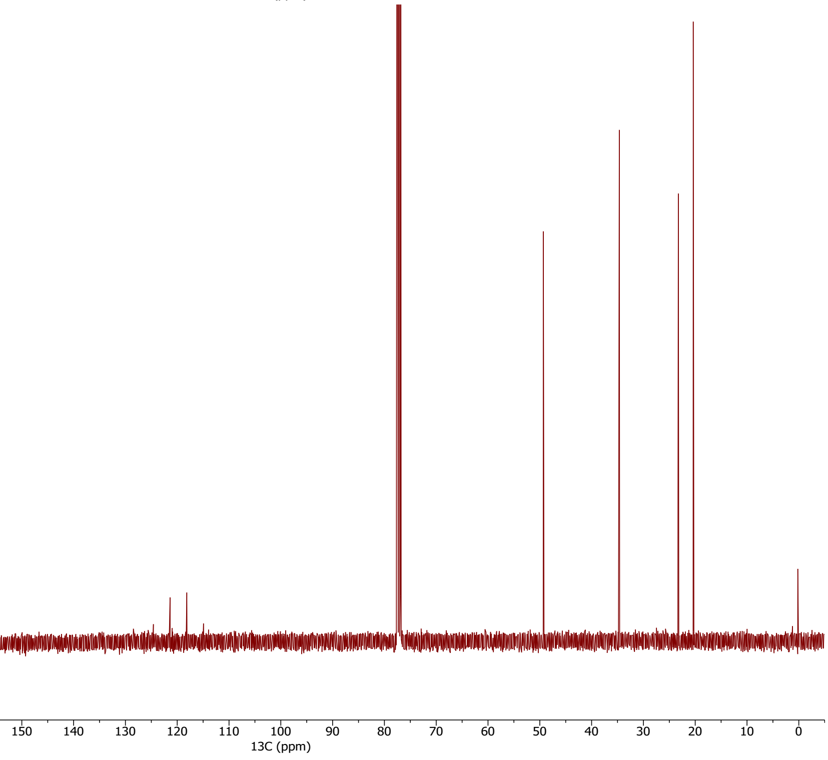

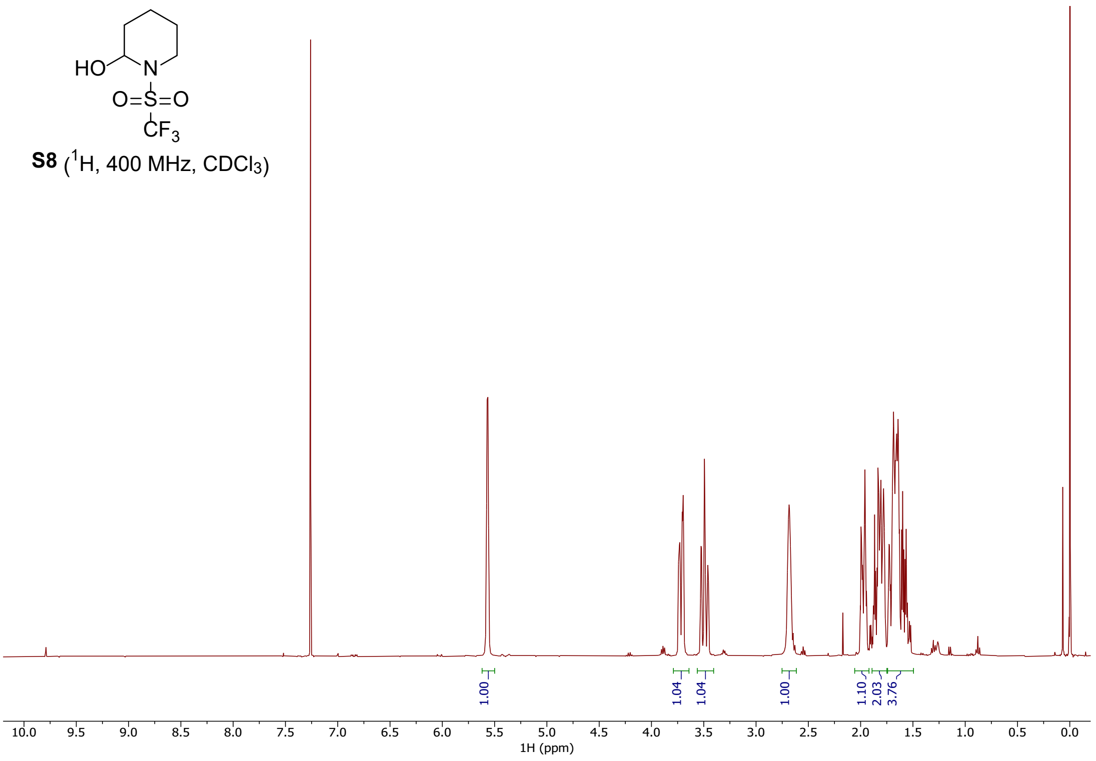
HO
N
O=S=O
CF3
S8 (1H, 400 MHz, CDCl3)
1.00
1.04
1.04
1.00
1.10
2.03
3.76
10.0
9.5
9.0
8.5
8.0
7.5
7.0
6.5
6.0
5.5
5.0
4.5
4.0
3.5
3.0
2.5
2.0
1.5
1.0
0.5
0.0
1H (ppm)

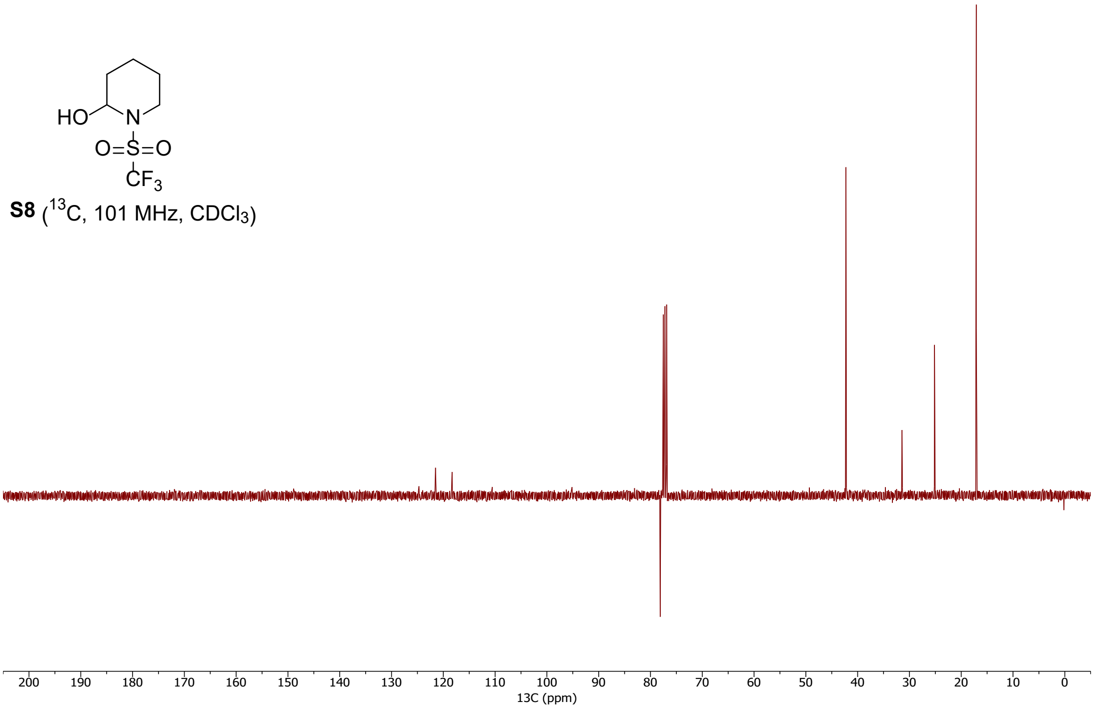
HO
N
O=S=O
CF3
S8 (13C, 101 MHz, CDCl3)
200
190
180
170
160
150
140
130
120
110
100
90
80
70
60
50
40
30
20
10
0
13C (ppm)

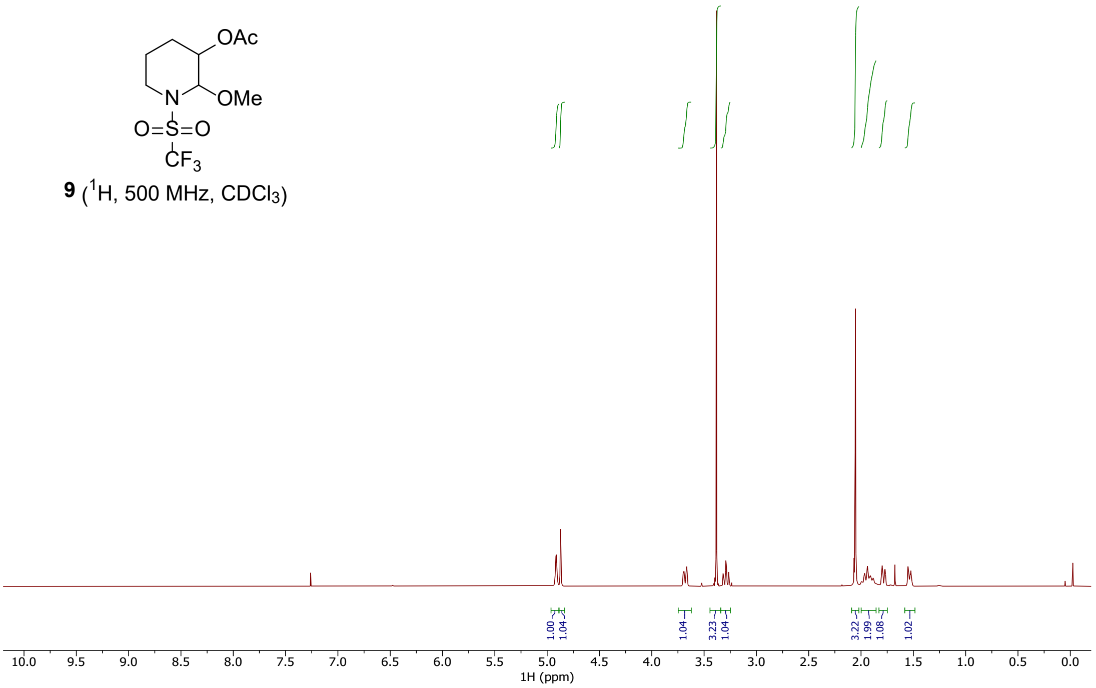

9 (^{1}H, 500 MHz, $CDCl_3$)

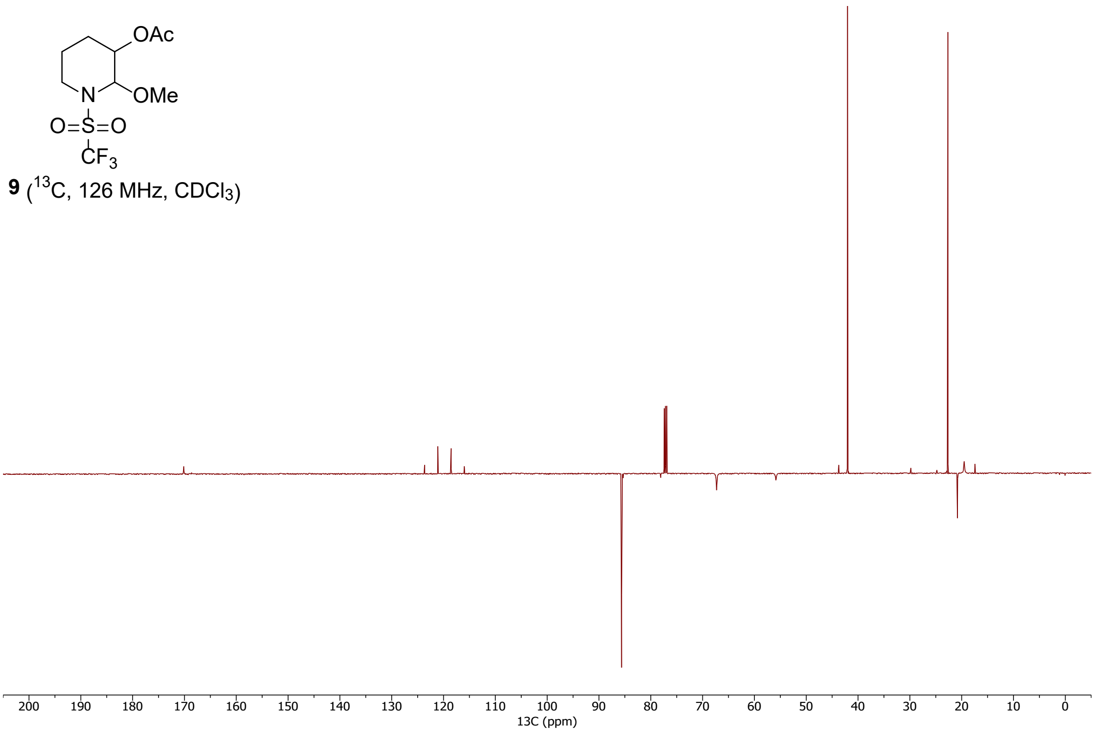

9 (^{13}C, 126 MHz, $CDCl_3$)

S12 (^{1}H, 500 MHz, $CDCl_3$)

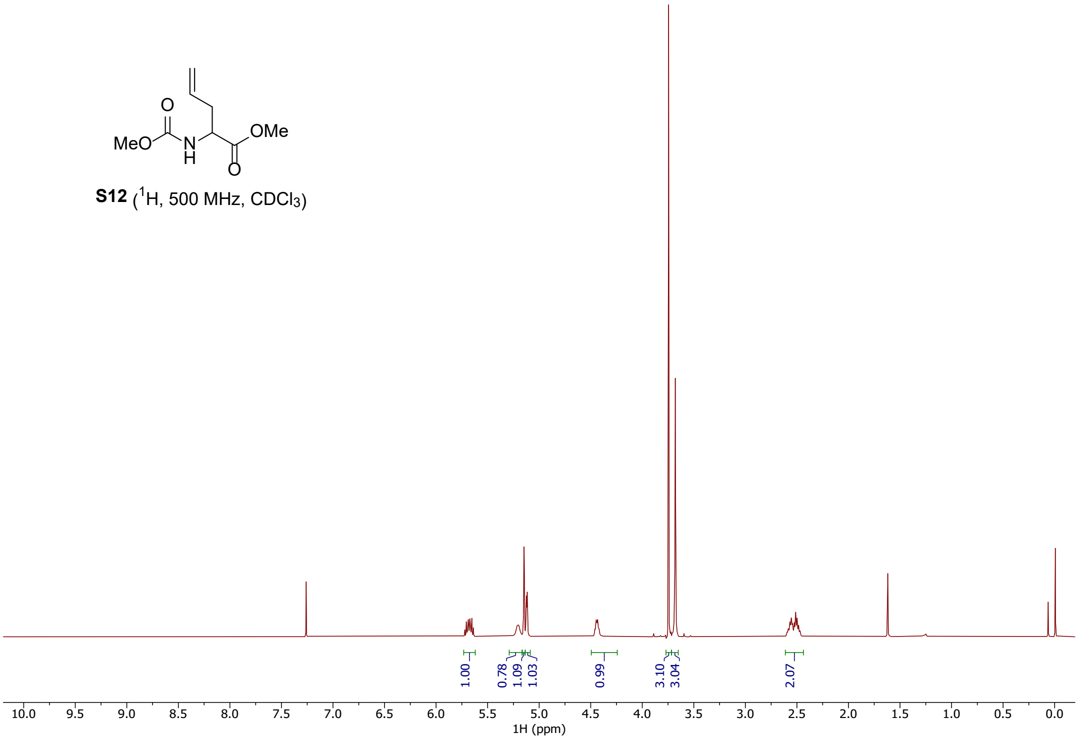

S12 (^{13}C, 126 MHz, $CDCl_3$)

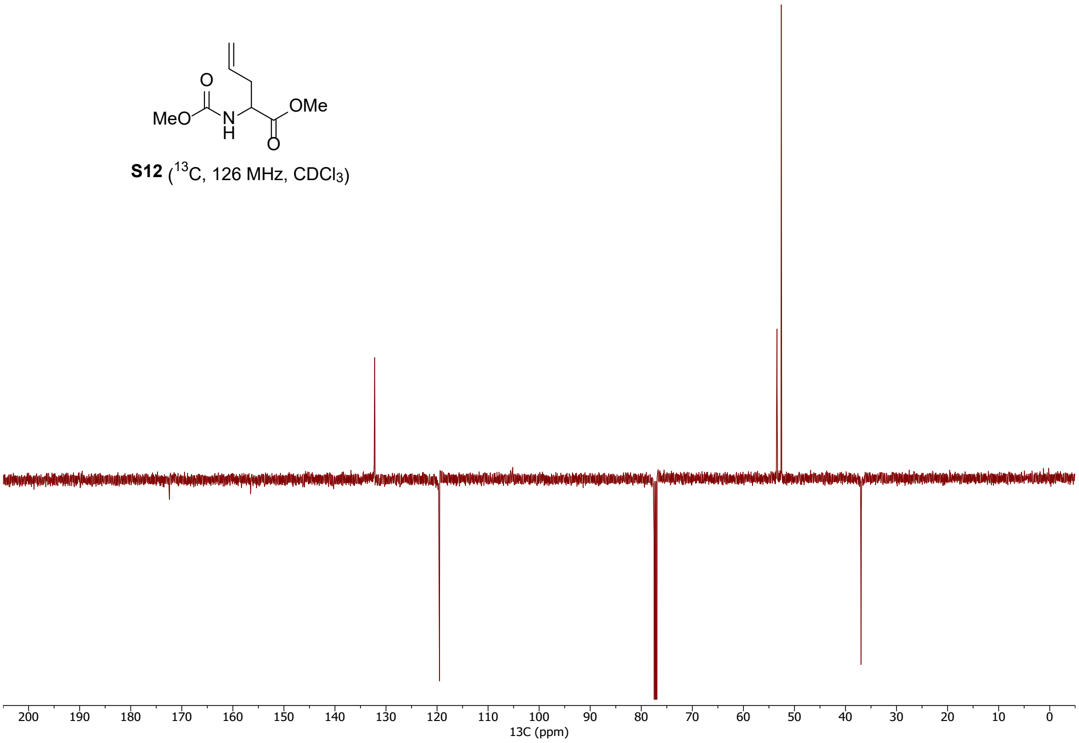

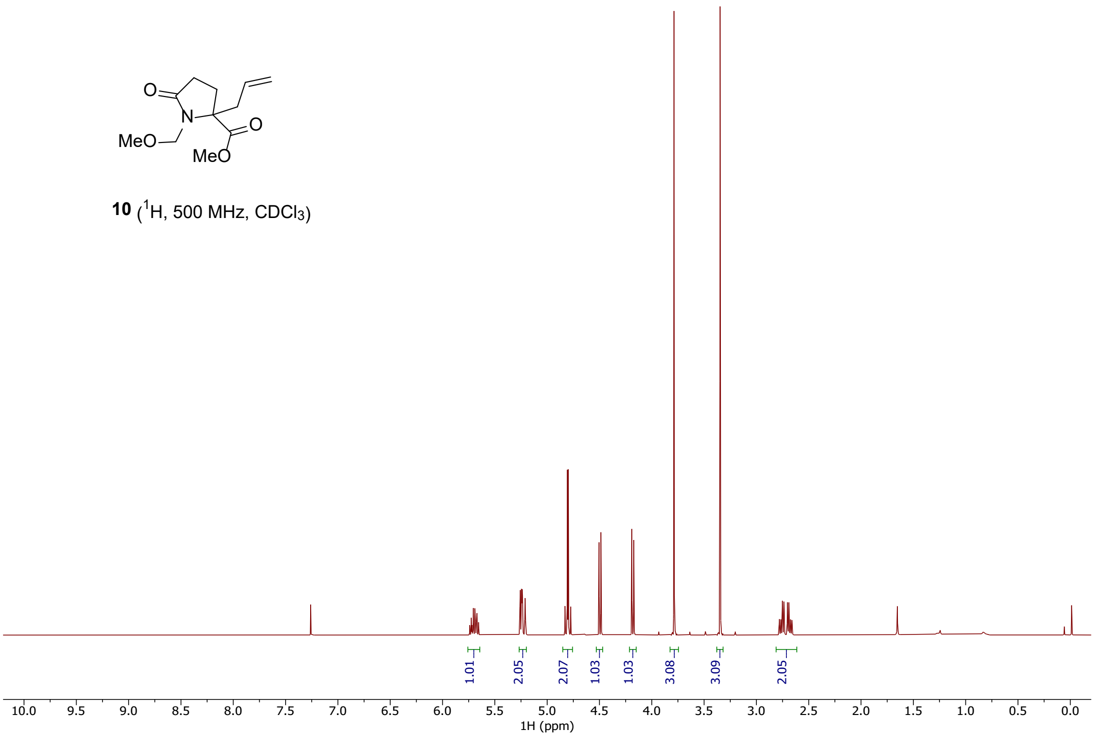

10 (^{1}H, 500 MHz, $CDCl_3$)

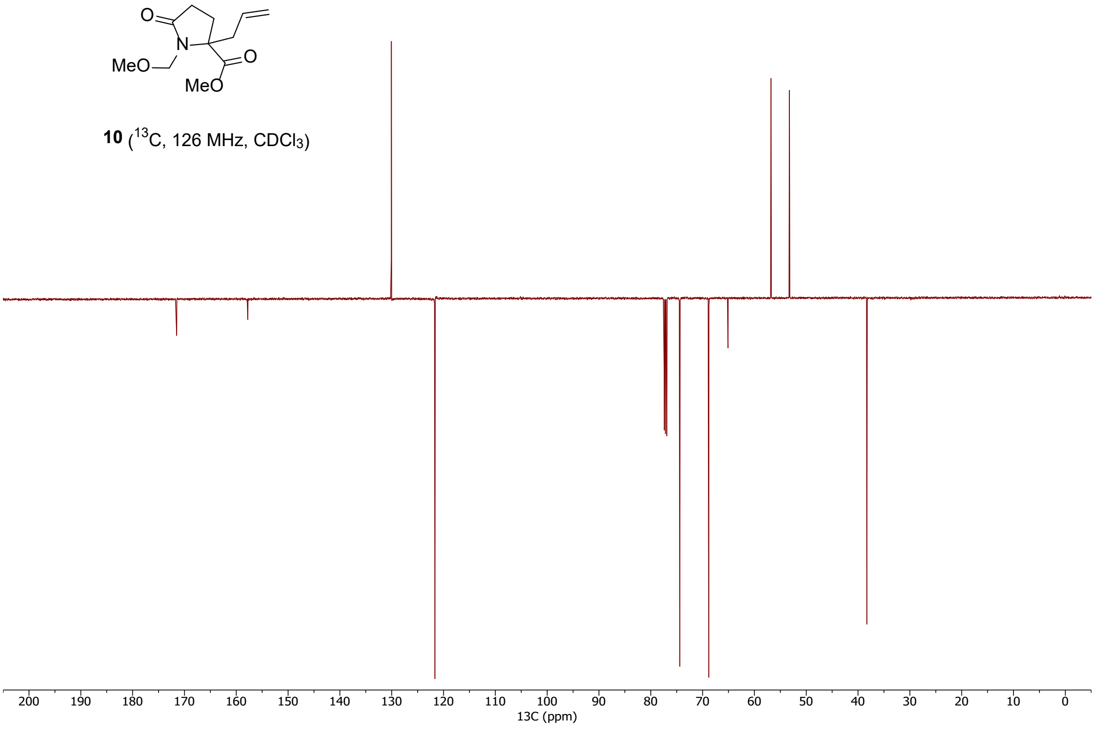

10 (^{13}C, 126 MHz, $CDCl_3$)

Gaussian Coordinates

5a (B3LYP, +0.0 kJ/mol)

8	1.785017000	1.559250000	-0.117744000
6	1.370900000	0.436768000	-0.028957000
8	2.022924000	-0.694828000	0.070093000
6	3.488263000	-0.599430000	0.077760000
7	-0.094238000	0.161967000	-0.007535000
6	-0.877790000	1.196306000	-0.038992000
6	-2.353375000	1.128330000	0.015771000
6	-2.911853000	-0.265893000	0.324745000
6	-2.061631000	-1.330565000	-0.375637000
6	-0.601259000	-1.249309000	0.061592000
1	3.801455000	0.004141000	0.929823000
1	3.820867000	-0.149690000	-0.857923000
1	3.827657000	-1.627937000	0.170039000
1	-0.369840000	2.157809000	-0.114530000
1	-2.700458000	1.509878000	-0.960365000
1	-2.686463000	1.894717000	0.729810000
1	-2.906206000	-0.432454000	1.408051000
1	-3.953587000	-0.324829000	0.001267000
1	-2.423375000	-2.336384000	-0.144384000
1	-2.126189000	-1.212957000	-1.464360000
1	-0.457525000	-1.582206000	1.094268000
1	0.050270000	-1.846479000	-0.575444000

5b (B3LYP, +94.5 kJ/mol)

6	3.392006000	0.081910000	0.491089000
8	2.135364000	0.763237000	0.107748000
6	1.118435000	0.059602000	-0.211320000
8	0.999895000	-1.268563000	-0.219043000
6	-0.436721000	-1.034188000	-0.619605000
6	-1.483693000	-1.368612000	0.413228000
6	-2.621215000	-0.320220000	0.298737000
6	-2.109410000	1.112047000	0.543825000
6	-1.022369000	1.525421000	-0.466716000
7	-0.088260000	0.401918000	-0.624985000
1	4.047124000	0.892888000	0.796839000
1	3.190024000	-0.601903000	1.315129000
1	3.784650000	-0.439861000	-0.381319000
1	-0.583142000	-1.451179000	-1.617344000
1	-1.854960000	-2.385478000	0.262134000
1	-1.030786000	-1.317174000	1.410023000
1	-3.103285000	-0.390273000	-0.685214000
1	-3.390142000	-0.567123000	1.035544000
1	-2.934470000	1.826690000	0.468686000
1	-1.708731000	1.189870000	1.561807000
1	-1.449868000	1.737138000	-1.451302000
1	-0.452859000	2.399821000	-0.145147000

6a (B3LYP, +0.0 kJ/mol)

8	-2.840133000	-0.859947000	-0.611691000
6	-1.964129000	-0.258088000	-0.056601000
8	-1.978637000	0.933560000	0.489639000
6	-3.243712000	1.671280000	0.392816000
7	-0.610433000	-0.855506000	0.103599000
6	-0.416305000	-2.011244000	-0.454463000
6	0.857692000	-2.756152000	-0.430193000
6	2.045570000	-1.996028000	0.173402000
6	1.564735000	-1.062041000	1.288765000
6	0.479266000	-0.095356000	0.781992000
6	1.028456000	0.923638000	-0.234838000
8	1.698710000	1.879202000	0.396131000
6	2.327109000	2.900058000	-0.434170000
8	0.877186000	0.814547000	-1.432933000
1	-3.052488000	2.609934000	0.906639000
1	-3.481200000	1.831330000	-0.659059000
1	-4.030146000	1.098395000	0.884394000
1	-1.292709000	-2.438293000	-0.941555000
1	1.047442000	-3.100055000	-1.456922000
1	0.630985000	-3.686515000	0.119552000
1	2.546851000	-1.422848000	-0.612788000
1	2.777709000	-2.707154000	0.563101000
1	2.386599000	-0.463175000	1.688532000
1	1.151854000	-1.638084000	2.125312000
1	0.027786000	0.441091000	1.615983000
1	1.565273000	3.414647000	-1.021172000
1	2.805914000	3.577301000	0.269441000
1	3.060072000	2.436680000	-1.096081000

6b (B3LYP, +52.2 kJ/mol)

8	-2.463481000	-0.375749000	-1.390047000
6	-1.939482000	-0.155312000	-0.322842000
8	-2.456420000	0.509472000	0.708399000
6	-3.828988000	0.972842000	0.541051000
7	-0.650568000	-0.654730000	0.003478000
6	0.229770000	-0.029431000	1.001925000
6	1.094556000	-1.130749000	1.666323000
6	1.928772000	-1.890348000	0.611105000
6	1.155207000	-2.150673000	-0.702819000
6	0.202891000	-1.027459000	-1.051408000
8	1.151817000	0.327405000	-1.122159000
6	1.108065000	0.790871000	0.062271000
8	1.766971000	1.815525000	0.450070000
6	2.635735000	2.536851000	-0.504416000
1	-3.887249000	1.655761000	-0.307179000
1	-4.066953000	1.480418000	1.473293000
1	-4.485420000	0.116816000	0.380781000
1	-0.321153000	0.576027000	1.718963000
1	1.743198000	-0.688732000	2.428389000
1	0.389927000	-1.794816000	2.175842000
1	2.834132000	-1.316004000	0.381169000
1	2.278730000	-2.838307000	1.026719000
1	0.517312000	-3.039374000	-0.617646000
1	1.841855000	-2.329381000	-1.534469000
1	-0.285752000	-1.063142000	-2.022278000
1	2.025098000	2.889541000	-1.335376000
1	3.041024000	3.361231000	0.075734000
1	3.418972000	1.860294000	-0.845541000

7a$_{eq}$ (B3LYP, +0.0 kJ/mol)

8	1.964538000	-1.602041000	0.928410000
6	2.035971000	-0.585336000	0.289957000
8	3.053086000	-0.108822000	-0.394631000
6	4.268851000	-0.924550000	-0.388467000
7	0.895208000	0.334761000	0.185759000
6	-0.235990000	-0.092827000	0.690381000
6	-1.453493000	0.782482000	0.822410000
8	-2.637334000	-0.032939000	0.669048000
6	-2.505300000	-0.996111000	-0.265004000
6	-3.769304000	-1.718208000	-0.592888000
8	-1.400062000	-1.207719000	-0.759392000
6	-1.452662000	1.974181000	-0.133153000
6	-0.057767000	2.611196000	-0.136297000
6	1.006288000	1.612014000	-0.584604000
1	4.974556000	-0.369891000	-1.001994000
1	4.627182000	-1.029360000	0.635944000
1	4.048032000	-1.901515000	-0.819116000
1	-0.168721000	-1.014439000	1.261441000
1	-1.490144000	1.115576000	1.867482000
1	-4.434905000	-1.044307000	-1.143244000
1	-4.283718000	-2.007241000	0.327608000
1	-3.546819000	-2.593040000	-1.202236000
1	-1.711628000	1.630659000	-1.140768000
1	-2.217324000	2.688853000	0.180197000
1	-0.033410000	3.465342000	-0.818587000
1	0.191695000	2.996936000	0.859826000
1	0.905676000	1.357667000	-1.644976000
1	2.011690000	1.996548000	-0.423382000

7a$_{eq}$ (M06-2x, +1.2 kJ/mol)

8	1.964161000	-1.595821000	0.928635000
6	2.035050000	-0.581755000	0.301496000
8	3.033328000	-0.109769000	-0.397741000
6	4.222052000	-0.944143000	-0.418615000
7	0.901148000	0.349416000	0.225990000
6	-0.216365000	-0.072178000	0.731564000
6	-1.451764000	0.779029000	0.817446000
8	-2.602444000	-0.053039000	0.690388000
6	-2.491577000	-0.983735000	-0.283200000
6	-3.761866000	-1.712086000	-0.560393000
8	-1.417431000	-1.153728000	-0.828742000
6	-1.443203000	1.923301000	-0.185731000
6	-0.072718000	2.596708000	-0.139887000
6	1.025828000	1.622042000	-0.537793000
1	4.932076000	-0.404379000	-1.038350000
1	4.592026000	-1.065317000	0.599269000
1	3.970692000	-1.912405000	-0.850889000
1	-0.168518000	-1.020950000	1.262934000
1	-1.492753000	1.160309000	1.846311000
1	-4.478622000	-1.017188000	-1.006520000
1	-4.189657000	-2.073997000	0.376904000
1	-3.565815000	-2.536788000	-1.242055000
1	-1.636901000	1.524429000	-1.187514000
1	-2.243395000	2.623094000	0.061963000
1	-0.039423000	3.445326000	-0.826493000
1	0.127645000	2.990551000	0.863500000
1	0.973402000	1.355968000	-1.598328000
1	2.017666000	2.021198000	-0.332308000

7b$_{eq}$ (B3LYP, +14.8 kJ/mol)

8	-1.864848000	-1.974018000	-0.061426000
6	-2.155388000	-0.813013000	-0.000553000
8	-3.325312000	-0.239341000	0.128547000
6	-4.481995000	-1.140964000	0.214300000
7	-1.099171000	0.244911000	-0.066925000
6	0.122554000	-0.168069000	-0.174417000
6	1.313739000	0.741199000	-0.223508000
8	2.364160000	0.149006000	0.544003000
6	3.125475000	-0.813268000	-0.108179000
6	4.285750000	-1.250617000	0.731404000
8	2.824956000	-1.195283000	-1.214382000
6	1.003015000	2.139481000	0.302100000
6	-0.314148000	2.609946000	-0.324902000
6	-1.479077000	1.693033000	0.044946000
1	-4.367338000	-1.784942000	1.086379000
1	-5.337094000	-0.477722000	0.316286000
1	-4.539783000	-1.732934000	-0.699270000
1	0.264179000	-1.246730000	-0.226709000
1	1.611745000	0.770537000	-1.284419000
1	3.957198000	-1.498304000	1.744302000
1	4.763759000	-2.110461000	0.263586000
1	5.006078000	-0.429573000	0.811273000
1	1.823911000	2.812654000	0.045763000
1	0.932075000	2.109166000	1.395129000
1	-0.218203000	2.663902000	-1.415974000
1	-0.563455000	3.618348000	0.016904000
1	-2.341645000	1.843271000	-0.604109000
1	-1.808439000	1.839139000	1.078255000

7b$_{eq}$ (M06-2x, +18.6 kJ/mol)

8	2.107525000	-1.453262000	0.908232000
6	1.997413000	-0.456092000	0.238358000
8	2.958006000	0.148188000	-0.439997000
6	4.258712000	-0.470950000	-0.342009000
7	0.778911000	0.239029000	0.089431000
6	0.688000000	1.403183000	-0.820247000
6	-0.719080000	1.993079000	-0.808888000
6	-1.254540000	2.051253000	0.618626000
6	-1.439303000	0.640473000	1.142004000
6	-0.309792000	-0.323305000	0.719129000
8	-1.103727000	-1.265902000	-0.252504000
6	-2.326074000	-0.938658000	-0.226021000
6	-3.366789000	-1.663386000	-0.983281000
8	-2.640202000	0.063885000	0.521526000
1	4.207371000	-1.492490000	-0.719077000
1	4.911945000	0.142538000	-0.956784000
1	4.585390000	-0.473866000	0.698003000
1	1.410523000	2.146199000	-0.473983000
1	0.983374000	1.094087000	-1.825431000
1	-1.395214000	1.406554000	-1.444689000
1	-0.680373000	2.991055000	-1.249631000
1	-2.208408000	2.580184000	0.687462000
1	-0.544780000	2.567638000	1.274552000
1	-1.617985000	0.596218000	2.216232000
1	0.019281000	-1.023775000	1.484598000
1	-4.179069000	-0.985211000	-1.243975000
1	-3.762946000	-2.451458000	-0.332841000
1	-2.925580000	-2.127730000	-1.864525000

7a$_{ax}$ (B3LYP, +4.1 kJ/mol)

8	2.112492000	-1.493599000	0.923378000
6	2.040286000	-0.489377000	0.256577000
8	3.005146000	0.094943000	-0.433805000
6	4.311476000	-0.553885000	-0.378356000
7	0.816973000	0.264035000	0.127639000
6	0.773930000	1.478603000	-0.739617000
6	-0.635143000	2.067625000	-0.811310000
6	-1.284672000	2.083996000	0.574275000
6	-1.436769000	0.662236000	1.100900000
6	-0.255980000	-0.248469000	0.747422000
8	-1.200161000	-1.334691000	-0.377437000
6	-2.388961000	-0.970992000	-0.250423000
6	-3.541942000	-1.641688000	-0.908203000
8	-2.648137000	0.055685000	0.530392000
1	4.231553000	-1.567559000	-0.772308000
1	4.953491000	0.063402000	-1.002498000
1	4.664168000	-0.575048000	0.653339000
1	1.476351000	2.200919000	-0.315548000
1	1.141729000	1.200130000	-1.728950000
1	-1.254464000	1.501554000	-1.517371000
1	-0.560612000	3.081112000	-1.214237000
1	-2.268220000	2.561628000	0.563367000
1	-0.663958000	2.646376000	1.282117000
1	-1.596634000	0.636130000	2.181088000
1	-0.001773000	-1.046727000	1.437926000
1	-4.307854000	-0.911620000	-1.176194000
1	-3.980534000	-2.346801000	-0.191544000
1	-3.200116000	-2.198743000	-1.780481000

7a$_{ax}$ (M06-2x, +0.0 kJ/mol)

8	2.107525000	-1.453262000	0.908232000
6	1.997413000	-0.456092000	0.238358000
8	2.958006000	0.148188000	-0.439997000
6	4.258712000	-0.470950000	-0.342009000
7	0.778911000	0.239029000	0.089431000
6	0.688000000	1.403183000	-0.820247000
6	-0.719080000	1.993079000	-0.808888000
6	-1.254540000	2.051253000	0.618626000
6	-1.439303000	0.640473000	1.142004000
6	-0.309792000	-0.323305000	0.719129000
8	-1.103727000	-1.265902000	-0.252504000
6	-2.326074000	-0.938658000	-0.226021000
6	-3.366789000	-1.663386000	-0.983281000
8	-2.640202000	0.063885000	0.521526000
1	4.207371000	-1.492490000	-0.719077000
1	4.911945000	0.142538000	-0.956784000
1	4.585390000	-0.473866000	0.698003000
1	1.410523000	2.146199000	-0.473983000
1	0.983374000	1.094087000	-1.825431000
1	-1.395214000	1.406554000	-1.444689000
1	-0.680373000	2.991055000	-1.249631000
1	-2.208408000	2.580184000	0.687462000
1	-0.544780000	2.567638000	1.274552000
1	-1.617985000	0.596218000	2.216232000
1	0.019281000	-1.023775000	1.484598000
1	-4.179069000	-0.985211000	-1.243975000
1	-3.762946000	-2.451458000	-0.332841000
1	-2.925580000	-2.127730000	-1.864525000

$7b_{ax}$ (B3LYP, +15.9 kJ/mol)

8	-1.965483000	-1.908877000	-0.256547000
6	-2.167558000	-0.744837000	-0.056809000
8	-3.277425000	-0.118649000	0.244027000
6	-4.480828000	-0.953588000	0.357123000
7	-1.053899000	0.251924000	-0.149363000
6	0.094880000	-0.203991000	-0.531473000
6	1.322298000	0.646719000	-0.706204000
8	2.222429000	0.234055000	0.346567000
6	3.004000000	-0.883668000	0.076078000
6	4.031230000	-1.099025000	1.145608000
8	2.821710000	-1.543434000	-0.918290000
6	1.051199000	2.141751000	-0.590647000
6	0.008545000	2.414782000	0.497091000
6	-1.299697000	1.694660000	0.186624000
1	-5.275778000	-0.253639000	0.601276000
1	-4.661119000	-1.448778000	-0.597175000
1	-4.332560000	-1.685643000	1.151158000
1	0.160641000	-1.276387000	-0.710668000
1	1.760312000	0.370855000	-1.673622000
1	3.578159000	-1.030286000	2.137932000
1	4.496380000	-2.073875000	1.003595000
1	4.795244000	-0.317352000	1.076381000
1	0.695607000	2.509226000	-1.561100000
1	1.994358000	2.650918000	-0.378663000
1	0.387760000	2.094216000	1.473855000
1	-0.208763000	3.483856000	0.571230000
1	-1.985210000	1.702910000	1.033208000
1	-1.822013000	2.128928000	-0.672254000

$7b_{ax}$ (M06-2x, +21.5 kJ/mol)

8	-1.970891000	-1.861174000	-0.339363000
6	-2.134833000	-0.712051000	-0.073252000
8	-3.214015000	-0.079525000	0.292642000
6	-4.411371000	-0.901553000	0.391921000
7	-1.004779000	0.257486000	-0.146358000
6	0.111746000	-0.199618000	-0.594514000
6	1.344168000	0.654574000	-0.773166000
8	2.252858000	0.291496000	0.268666000
6	2.886248000	-0.919891000	0.109906000
6	3.959923000	-1.116768000	1.131326000
8	2.559325000	-1.672292000	-0.769471000
6	1.046214000	2.137954000	-0.658957000
6	0.102491000	2.377747000	0.515471000
6	-1.223206000	1.673127000	0.279703000
1	-5.195685000	-0.215362000	0.697225000
1	-4.621284000	-1.341908000	-0.582496000
1	-4.247033000	-1.679325000	1.137226000
1	0.150129000	-1.261877000	-0.837742000
1	1.774679000	0.380235000	-1.743480000
1	3.564790000	-0.936058000	2.133005000
1	4.346980000	-2.130154000	1.047905000
1	4.761548000	-0.395312000	0.953059000
1	0.594067000	2.485306000	-1.595147000
1	1.991420000	2.669300000	-0.531374000
1	0.560090000	2.014622000	1.441818000
1	-0.102700000	3.442228000	0.647237000
1	-1.836559000	1.632227000	1.179203000
1	-1.813484000	2.144506000	-0.512349000

$8a_{eq}$ (B3LYP, +0.0 kJ/mol)

6	3.917418000	-1.699897000	0.322275000
6	2.627780000	-0.965812000	0.150358000
8	2.695893000	0.046673000	-0.745239000
6	1.528195000	0.890778000	-0.797800000
6	0.279562000	0.051387000	-0.673812000
7	-0.791928000	0.472857000	-0.079703000
16	-2.310079000	-0.587147000	-0.208178000
6	-2.271652000	-1.448221000	1.369360000
8	-3.394509000	0.377958000	-0.249883000
8	-1.987811000	-1.502875000	-1.292285000
6	-0.869951000	1.722178000	0.725621000
6	0.212183000	2.711937000	0.290360000
6	1.585418000	2.030401000	0.218579000
8	1.571762000	-1.205046000	0.724518000
1	3.753934000	-2.597393000	0.917162000
1	4.638522000	-1.048220000	0.827387000
1	4.336913000	-1.955060000	-0.654704000
1	1.530482000	1.282812000	-1.823287000
1	0.182278000	-0.850876000	-1.274055000
1	-1.325908000	-1.986506000	1.447681000
1	-3.115000000	-2.143601000	1.339098000
1	-2.407937000	-0.720917000	2.170524000
1	-1.873461000	2.127340000	0.583709000
1	-0.750836000	1.439111000	1.778022000
1	0.232530000	3.535443000	1.009466000
1	-0.056136000	3.145575000	-0.680287000
1	1.860150000	1.628176000	1.199974000
1	2.362844000	2.738295000	-0.078466000

$8a_{eq}$ (M06-2x, +0.0 kJ/mol)

6	3.849656000	-1.694734000	0.305622000
6	2.571121000	-0.940700000	0.161165000
8	2.640553000	0.043852000	-0.764411000
6	1.510803000	0.911457000	-0.809323000
6	0.246924000	0.093694000	-0.751767000
7	-0.817221000	0.490253000	-0.147578000
16	-2.264092000	-0.605181000	-0.192170000
6	-2.062830000	-1.446399000	1.359835000
8	-3.376664000	0.306726000	-0.158077000
8	-1.975260000	-1.494602000	-1.289662000
6	-0.909571000	1.719698000	0.678312000
6	0.194512000	2.697824000	0.297559000
6	1.550025000	1.991281000	0.263371000
8	1.534919000	-1.137107000	0.766282000
1	3.698537000	-2.542037000	0.970751000
1	4.613486000	-1.026923000	0.712966000
1	4.192225000	-2.028355000	-0.676324000
1	1.542116000	1.356859000	-1.812200000
1	0.170831000	-0.822374000	-1.338446000
1	-1.064869000	-1.888721000	1.382243000
1	-2.835208000	-2.219647000	1.376079000
1	-2.223855000	-0.731310000	2.167011000
1	-1.907278000	2.131171000	0.514668000
1	-0.820240000	1.406676000	1.725217000
1	0.200942000	3.508821000	1.028858000
1	-0.032805000	3.146076000	-0.676401000
1	1.761453000	1.527976000	1.233423000
1	2.358457000	2.689250000	0.038243000

$8b_{eq}$ (B3LYP, +18.7 kJ/mol)

6	-4.372444000	-1.276317000	-0.623210000
6	-3.173527000	-0.829274000	0.153766000
8	-2.534748000	0.234539000	-0.466393000
6	-1.463371000	0.829845000	0.264281000
6	-0.242053000	-0.049229000	0.150746000
7	0.954399000	0.412340000	0.057124000
16	2.346413000	-0.832650000	-0.251256000
6	3.176509000	-0.845773000	1.343497000
8	3.150084000	-0.163436000	-1.257152000
8	1.629004000	-2.078720000	-0.461601000
6	1.303375000	1.859500000	-0.038547000
6	0.117045000	2.748370000	0.346992000
6	-1.195608000	2.238184000	-0.260134000
8	-2.751218000	-1.283205000	1.192441000
1	-4.761161000	-2.196061000	-0.187793000
1	-5.141924000	-0.498019000	-0.585553000
1	-4.113273000	-1.429789000	-1.674354000
1	-1.715761000	0.845577000	1.335803000
1	-0.364722000	-1.132446000	0.135532000
1	2.473922000	-1.197615000	2.099892000
1	3.563202000	0.151645000	1.554562000
1	4.001914000	-1.554049000	1.225982000
1	2.165172000	2.033656000	0.610548000
1	1.628972000	2.020979000	-1.070589000
1	0.034335000	2.802579000	1.438918000
1	0.332982000	3.761615000	-0.003346000
1	-1.141820000	2.214081000	-1.354219000
1	-2.029776000	2.888614000	0.012141000

$8b_{eq}$ (M06-2x, +19.8 kJ/mol)

6	-4.345706000	-1.248729000	-0.580956000
6	-3.118643000	-0.812649000	0.150237000
8	-2.537731000	0.285217000	-0.441249000
6	-1.449143000	0.848086000	0.263934000
6	-0.252456000	-0.059018000	0.130627000
7	0.948018000	0.384331000	0.043731000
16	2.294297000	-0.826875000	-0.253509000
6	3.061292000	-0.887599000	1.347008000
8	3.123136000	-0.137436000	-1.205317000
8	1.585740000	-2.051313000	-0.521058000
6	1.326606000	1.822171000	-0.027506000
6	0.158230000	2.719223000	0.366356000
6	-1.145638000	2.238710000	-0.269833000
8	-2.629932000	-1.305460000	1.134468000
1	-4.720951000	-2.166041000	-0.132287000
1	-5.102123000	-0.462313000	-0.517855000
1	-4.114754000	-1.403739000	-1.637152000
1	-1.677925000	0.879203000	1.340522000
1	-0.393483000	-1.141000000	0.091921000
1	2.331261000	-1.256797000	2.067876000
1	3.445631000	0.101150000	1.597974000
1	3.887271000	-1.596227000	1.239694000
1	2.190551000	1.964714000	0.626320000
1	1.656335000	1.988244000	-1.057196000
1	0.059546000	2.744288000	1.457349000
1	0.394425000	3.735530000	0.043516000
1	-1.063618000	2.198530000	-1.361529000
1	-1.974002000	2.904903000	-0.023074000

8a$_{ax}$ (B3LYP, +15.2 kJ/mol)

6	3.839280000	-1.664888000	0.493950000
6	2.549511000	-1.001368000	0.132136000
8	2.694981000	0.042624000	-0.702444000
6	1.504281000	0.809136000	-1.022660000
6	0.247479000	-0.005172000	-0.764872000
7	-0.755531000	0.466179000	-0.086051000
16	-2.360169000	-0.437198000	-0.193866000
6	-2.322482000	-1.425325000	1.307964000
8	-3.347576000	0.623322000	-0.097546000
8	-2.190705000	-1.293447000	-1.358663000
6	-0.702597000	1.676189000	0.779936000
6	0.745304000	2.102660000	1.019035000
6	1.505010000	2.164819000	-0.310025000
8	1.433454000	-1.338298000	0.516663000
1	3.647366000	-2.500760000	1.164884000
1	4.509068000	-0.941579000	0.968625000
1	4.333651000	-2.018370000	-0.416376000
1	1.562676000	0.942111000	-2.108699000
1	0.042721000	-0.873766000	-1.385082000
1	-3.229346000	-2.035167000	1.272223000
1	-2.347119000	-0.761725000	2.172741000
1	-1.430576000	-2.053135000	1.287838000
1	-1.286676000	2.455089000	0.280902000
1	-1.209746000	1.434556000	1.717327000
1	0.735861000	3.083894000	1.501494000
1	1.230593000	1.408608000	1.714130000
1	2.541935000	2.480750000	-0.170419000
1	1.035072000	2.901489000	-0.972236000

8c$_{ax}$ (M06-2x, +11.5 kJ/mol)

6	-3.360237000	0.623225000	-0.591405000
16	-2.148895000	-0.519445000	0.029567000
7	-0.681993000	0.396039000	-0.055202000
6	-0.448251000	1.352787000	1.054866000
6	0.968184000	1.927743000	0.991686000
6	1.402551000	2.134131000	-0.456838000
6	1.500051000	0.786605000	-1.146286000
6	0.354117000	-0.174781000	-0.759981000
8	1.157444000	-1.214328000	0.109105000
6	2.389042000	-0.938535000	0.030994000
6	3.434246000	-1.722621000	0.719488000
8	2.705846000	0.099785000	-0.668679000
8	-1.965816000	-1.594276000	-0.927390000
8	-2.399142000	-0.759161000	1.433625000
1	-4.314931000	0.095704000	-0.537810000
1	-3.384082000	1.502700000	0.052432000
1	-3.111198000	0.870580000	-1.622648000
1	-0.636410000	0.849768000	2.006525000
1	-1.178302000	2.160580000	0.943714000
1	0.987191000	2.870367000	1.541820000
1	1.675182000	1.264639000	1.506195000
1	0.669786000	2.742337000	-0.998268000
1	2.368243000	2.640082000	-0.531900000
1	1.613113000	0.858410000	-2.227827000
1	-0.015480000	-0.810336000	-1.562574000
1	3.598097000	-1.270017000	1.704190000
1	4.365434000	-1.674778000	0.154979000
1	3.094752000	-2.748683000	0.856093000

8b$_{ax}$ (B3LYP, +18.4 kJ/mol)

6	4.133968000	-1.178654000	0.993963000
6	3.072172000	-0.897805000	-0.024732000
8	2.384411000	0.272128000	0.272718000
6	1.456144000	0.732089000	-0.730158000
6	0.196799000	-0.078773000	-0.521504000
7	-0.916402000	0.426628000	-0.124739000
16	-2.339394000	-0.777769000	0.231530000
6	-3.458969000	-0.411411000	-1.126164000
8	-1.717391000	-2.078200000	0.050131000
8	-2.868370000	-0.284873000	1.490082000
6	-1.113033000	1.861681000	0.232079000
6	0.227611000	2.529027000	0.538775000
6	1.240063000	2.234163000	-0.572970000
8	2.793165000	-1.549766000	-1.002656000
1	3.727929000	-1.099819000	2.005931000
1	4.933357000	-0.435993000	0.900535000
1	4.540362000	-2.174688000	0.822200000
1	1.842133000	0.464964000	-1.721662000
1	0.240114000	-1.157300000	-0.678704000
1	-3.767998000	0.632366000	-1.058375000
1	-4.317739000	-1.071526000	-0.972878000
1	-2.957893000	-0.646116000	-2.066018000
1	-1.786049000	1.876381000	1.092210000
1	-1.619100000	2.336136000	-0.616889000
1	0.604048000	2.172018000	1.503510000
1	0.054370000	3.604360000	0.635432000
1	0.883061000	2.638735000	-1.528078000
1	2.205482000	2.702191000	-0.366452000

8b$_{ax}$ (M06-2x, +23.2kJ/mol)

6	4.072559000	-1.163579000	0.978549000
6	2.988133000	-0.893892000	-0.013762000
8	2.379158000	0.319306000	0.219667000
6	1.446458000	0.742608000	-0.774233000
6	0.199722000	-0.090541000	-0.562843000
7	-0.896372000	0.396841000	-0.110815000
16	-2.281015000	-0.771311000	0.217844000
6	-3.435599000	-0.273948000	-1.035433000
8	-1.707976000	-2.055597000	-0.088810000
8	-2.731555000	-0.371084000	1.523899000
6	-1.089912000	1.812856000	0.305742000
6	0.254878000	2.474098000	0.572351000
6	1.194864000	2.231013000	-0.605647000
8	2.631603000	-1.592319000	-0.925611000
1	3.683656000	-1.063511000	1.994119000
1	4.869792000	-0.426784000	0.851772000
1	4.461207000	-2.166496000	0.814958000
1	1.830752000	0.487774000	-1.769034000
1	0.238329000	-1.163211000	-0.765362000
1	-3.719567000	0.764391000	-0.860625000
1	-4.301818000	-0.928401000	-0.904108000
1	-2.979705000	-0.433769000	-2.012917000
1	-1.724044000	1.784436000	1.194642000
1	-1.632162000	2.310980000	-0.506102000
1	0.688473000	2.070972000	1.493513000
1	0.083284000	3.541369000	0.727523000
1	0.758618000	2.626378000	-1.530328000
1	2.157571000	2.724811000	-0.459354000

$9b_{ax}$ (B3LYP, +0.0 kJ/mol)

8	2.637729000	1.504310000	0.592760000
16	1.794790000	0.338802000	0.762999000
7	0.158924000	0.849574000	0.180062000
6	0.069304000	1.868758000	-0.911374000
6	-1.201650000	2.700827000	-0.745195000
6	-2.432761000	1.794787000	-0.610574000
6	-2.272387000	0.893321000	0.612171000
6	-0.887309000	0.296499000	0.749625000
8	-1.597012000	-1.357537000	-0.308295000
6	-2.786965000	-1.294652000	0.011687000
6	-3.807231000	-2.359617000	-0.201713000
8	-3.239006000	-0.182773000	0.612541000
6	2.273287000	-0.954582000	-0.560974000
9	3.518923000	-1.320383000	-0.320653000
9	2.169980000	-0.384694000	-1.761127000
9	1.445541000	-1.985713000	-0.468985000
8	1.554179000	-0.358389000	2.013312000
1	0.967071000	2.484070000	-0.845286000
1	0.079552000	1.330575000	-1.864576000
1	-1.302801000	3.347504000	-1.621261000
1	-1.103312000	3.358887000	0.126201000
1	-3.348674000	2.381130000	-0.505157000
1	-2.540251000	1.173436000	-1.506956000
1	-2.470033000	1.442059000	1.540925000
1	-0.682640000	-0.357218000	1.593916000
1	-3.320207000	-3.283164000	-0.511522000
1	-4.508089000	-2.032773000	-0.977874000
1	-4.382578000	-2.512173000	0.715595000

$9b_{ax}$ (MP2, +0.5 kJ/mol)

8	2.518114000	1.606022000	0.420523000
16	1.708004000	0.436474000	0.702108000
7	0.128236000	0.778957000	0.120644000
6	-0.060481000	1.687915000	-1.036981000
6	-1.238634000	2.604234000	-0.753616000
6	-2.493580000	1.765203000	-0.508740000
6	-2.274885000	0.851105000	0.683203000
6	-0.932712000	0.130059000	0.697962000
8	-1.390064000	-1.196756000	-0.129389000
6	-2.651155000	-1.287291000	0.053340000
6	-3.446503000	-2.459919000	-0.363161000
8	-3.244342000	-0.267705000	0.617059000
6	2.239430000	-0.897343000	-0.482548000
9	3.495853000	-1.239756000	-0.223322000
9	2.130393000	-0.427110000	-1.735145000
9	1.426593000	-1.954469000	-0.335988000
8	1.535502000	-0.192765000	2.002979000
1	0.862715000	2.251402000	-1.151102000
1	-0.223573000	1.092052000	-1.939130000
1	-1.397964000	3.260117000	-1.610101000
1	-1.012741000	3.237476000	0.107665000
1	-3.372428000	2.383790000	-0.329974000
1	-2.693627000	1.154059000	-1.394076000
1	-2.454330000	1.337328000	1.640657000
1	-0.667383000	-0.319093000	1.653575000
1	-2.806972000	-3.334208000	-0.434046000
1	-3.879912000	-2.245825000	-1.342239000
1	-4.257606000	-2.615513000	0.344499000

$9c_{eq}$ (B3LYP, +19.1 kJ/mol)

6	4.873396000	-1.521245000	0.476968000
6	3.604451000	-1.081158000	-0.182511000
8	3.180711000	0.151264000	0.301175000
6	2.069462000	0.736793000	-0.373169000
6	0.804732000	0.047453000	0.074907000
7	-0.308872000	0.669408000	0.260854000
16	-1.741329000	-0.322236000	0.936525000
8	-1.150734000	-1.570042000	1.376942000
6	-2.695225000	-0.647956000	-0.698597000
9	-3.748138000	-1.377936000	-0.396557000
9	-3.052590000	0.522746000	-1.216340000
9	-1.880179000	-1.290196000	-1.525402000
8	-2.473756000	0.619837000	1.754537000
6	-0.496149000	2.151938000	0.142899000
6	0.683993000	2.793527000	-0.591049000
6	2.028404000	2.239024000	-0.104716000
8	2.967095000	-1.659637000	-1.031589000
1	5.087159000	-2.551347000	0.194315000
1	5.694882000	-0.873911000	0.151911000
1	4.794533000	-1.429659000	1.563632000
1	2.140013000	0.525644000	-1.452403000
1	0.824052000	-1.026990000	0.257525000
1	-0.603092000	2.521568000	1.167101000
1	-1.441496000	2.321030000	-0.375391000
1	0.574527000	2.639925000	-1.670971000
1	0.630195000	3.872354000	-0.419381000
1	2.166234000	2.419414000	0.967138000
1	2.859624000	2.718740000	-0.626066000

$9c_{eq}$ (M06-2x, +35.3kJ/mol)

6	4.790238000	-1.515487000	0.455453000
6	3.501253000	-1.071327000	-0.152663000
8	3.146681000	0.191503000	0.270453000
6	2.021828000	0.753160000	-0.373514000
6	0.776276000	0.055967000	0.108366000
7	-0.340135000	0.672405000	0.279812000
16	-1.734235000	-0.278903000	0.936212000
8	-1.155687000	-1.475943000	1.475780000
6	-2.549816000	-0.702755000	-0.701285000
9	-3.633814000	-1.387868000	-0.444784000
9	-2.832282000	0.428477000	-1.323408000
9	-1.686852000	-1.407840000	-1.406165000
8	-2.524593000	0.687075000	1.640077000
6	-0.548622000	2.145863000	0.119724000
6	0.621303000	2.773508000	-0.628009000
6	1.959423000	2.248993000	-0.108635000
8	2.795055000	-1.674744000	-0.918448000
1	4.975342000	-2.551778000	0.180749000
1	5.599440000	-0.879590000	0.086967000
1	4.749167000	-1.405560000	1.541244000
1	2.063486000	0.543588000	-1.454780000
1	0.807511000	-1.012979000	0.329270000
1	-0.657573000	2.538006000	1.135051000
1	-1.497721000	2.282652000	-0.401134000
1	0.524234000	2.574515000	-1.701058000
1	0.553695000	3.855805000	-0.496917000
1	2.068367000	2.431529000	0.966033000
1	2.796347000	2.733851000	-0.613802000

$9a_{eq}$ (B3LYP, +0.2 kJ/mol)

8	1.852363000	-0.868539000	-1.854346000
16	1.469749000	-0.851454000	-0.455856000
7	0.062578000	0.160793000	-0.320750000
6	-0.143998000	1.203765000	-1.377547000
6	-1.467686000	1.945421000	-1.160071000
6	-1.717570000	2.203331000	0.328551000
6	-1.860896000	0.885971000	1.075838000
6	-0.900543000	-0.218874000	0.578909000
8	-2.004474000	-1.232100000	-0.098170000
6	-3.157975000	-0.785748000	0.180757000
6	-4.407063000	-1.490438000	-0.187170000
8	-3.215590000	0.334635000	0.839345000
6	2.746083000	0.193628000	0.484351000
9	2.294273000	0.401682000	1.726298000
9	2.902272000	1.367025000	-0.132904000
9	3.889686000	-0.468595000	0.515385000
8	1.217440000	-2.028266000	0.361641000
1	0.694701000	1.900531000	-1.311449000
1	-0.109719000	0.721225000	-2.356196000
1	-2.302039000	1.382522000	-1.595377000
1	-1.421117000	2.888638000	-1.710472000
1	-0.879602000	2.753627000	0.772623000
1	-2.616330000	2.803522000	0.496430000
1	-1.790074000	1.000101000	2.158332000
1	-0.509092000	-0.882301000	1.345969000
1	-4.253218000	-2.072454000	-1.097137000
1	-4.655312000	-2.186006000	0.625055000
1	-5.228320000	-0.780871000	-0.297311000

$9b_{eq}$ (MP2, +0.0 kJ/mol)

8	-1.878663000	-0.873040000	1.842366000
16	-1.471617000	-0.859148000	0.449601000
7	-0.080403000	0.133123000	0.333026000
6	0.188875000	1.080967000	1.453355000
6	1.490146000	1.835464000	1.196758000
6	1.624212000	2.179167000	-0.281934000
6	1.793865000	0.902879000	-1.075101000
6	0.876967000	-0.235482000	-0.591031000
8	1.948759000	-1.223690000	0.043073000
6	3.109743000	-0.748834000	-0.207852000
6	4.351809000	-1.452910000	0.170156000
8	3.166419000	0.384037000	-0.843554000
6	-2.666606000	0.219895000	-0.483331000
9	-2.184942000	0.414191000	-1.724276000
9	-2.769999000	1.404990000	0.136849000
9	-3.850750000	-0.378178000	-0.539933000
8	-1.235766000	-2.027792000	-0.386753000
1	-0.647826000	1.777348000	1.492097000
1	0.221615000	0.526936000	2.390676000
1	2.350668000	1.248751000	1.530069000
1	1.486501000	2.738719000	1.806326000
1	0.725166000	2.684744000	-0.644571000
1	2.471844000	2.835452000	-0.481454000
1	1.722097000	1.038929000	-2.152285000
1	0.460476000	-0.861219000	-1.375340000
1	4.179801000	-2.038604000	1.068976000
1	4.611996000	-2.131700000	-0.644870000
1	5.157513000	-0.736866000	0.304639000

9c$_{ax}$ (B3LYP, +18.4 kJ/mol)

6	4.828484000	-1.257697000	0.723968000
6	3.624745000	-1.028400000	-0.136566000
8	3.053116000	0.215605000	0.113811000
6	2.008218000	0.623906000	-0.791332000
6	0.761864000	-0.082617000	-0.308490000
7	-0.250387000	0.534307000	0.196043000
16	-1.654317000	-0.523340000	0.847630000
8	-2.162754000	0.210299000	1.986666000
6	-2.873156000	-0.354135000	-0.625613000
9	-3.185155000	0.929995000	-0.760625000
9	-2.260037000	-0.798569000	-1.716234000
9	-3.935042000	-1.078099000	-0.342248000
8	-1.123697000	-1.871536000	0.858149000
6	-0.327293000	2.014627000	0.411534000
6	1.076334000	2.617842000	0.433313000
6	1.885594000	2.144008000	-0.778794000
8	3.150697000	-1.769533000	-0.963798000
1	5.139114000	-2.298082000	0.634762000
1	4.612334000	-1.009149000	1.766274000
1	5.641782000	-0.604199000	0.390635000
1	2.223686000	0.225611000	-1.791000000
1	0.734088000	-1.171880000	-0.353697000
1	-0.858643000	2.162465000	1.353760000
1	-0.941252000	2.420184000	-0.399543000
1	1.584047000	2.343349000	1.364314000
1	0.969161000	3.706116000	0.430862000
1	2.891417000	2.570601000	-0.776707000
1	1.400517000	2.464455000	-1.708830000

9c$_{ax}$ (M06-2x, +36.5 kJ/mol)

6	4.767656000	-1.215675000	0.684177000
6	3.532765000	-1.001658000	-0.128733000
8	3.020877000	0.266407000	0.050074000
6	1.957184000	0.631722000	-0.827731000
6	0.732611000	-0.108233000	-0.332691000
7	-0.257089000	0.485747000	0.233633000
16	-1.622587000	-0.555986000	0.830902000
8	-2.135335000	0.134850000	1.976817000
6	-2.780756000	-0.310513000	-0.625641000
9	-3.050479000	0.979023000	-0.717305000
9	-2.152283000	-0.723777000	-1.710270000
9	-3.863013000	-1.009825000	-0.403937000
8	-1.115717000	-1.897487000	0.792009000
6	-0.329103000	1.952430000	0.520474000
6	1.067690000	2.554206000	0.489643000
6	1.786801000	2.140053000	-0.791834000
8	2.989062000	-1.778945000	-0.867841000
1	5.071512000	-2.256923000	0.599422000
1	4.580762000	-0.952709000	1.727425000
1	5.561216000	-0.562642000	0.311791000
1	2.162840000	0.249383000	-1.835154000
1	0.700666000	-1.196157000	-0.425880000
1	-0.809423000	2.047736000	1.496097000
1	-0.985873000	2.387770000	-0.239379000
1	1.635343000	2.226314000	1.366817000
1	0.963972000	3.639647000	0.551169000
1	2.776709000	2.595298000	-0.861214000
1	1.215089000	2.456450000	-1.671797000

11 (B3LYP, +40.6 kJ/mol)

8	3.164059000	-1.167350000	-0.626226000
6	2.084690000	-1.114054000	-0.132471000
8	1.574682000	-1.794761000	0.880006000
6	0.161655000	-1.528217000	1.081547000
6	-0.112932000	-0.197733000	0.332796000
6	-1.456004000	-0.193867000	-0.422040000
8	-2.415532000	-0.634816000	0.377342000
6	-3.775868000	-0.659021000	-0.163839000
8	-1.581043000	0.191330000	-1.563905000
6	-0.004982000	1.042645000	1.300107000
6	-0.022450000	2.365565000	0.594622000
6	1.052520000	3.159152000	0.477763000
7	1.022243000	-0.169940000	-0.597763000
6	1.218968000	0.607382000	-1.607342000
1	-0.398471000	-2.370600000	0.671960000
1	-0.007762000	-1.451912000	2.154467000
1	-3.806897000	-1.311760000	-1.036721000
1	-4.075979000	0.353410000	-0.435900000
1	-4.388748000	-1.049576000	0.644943000
1	0.916147000	0.926066000	1.881990000
1	-0.848569000	0.951035000	1.993133000
1	-0.972576000	2.689502000	0.171738000
1	0.991557000	4.121941000	-0.020376000
1	2.013349000	2.895198000	0.914827000
1	0.432583000	1.268064000	-1.945247000
1	2.178757000	0.521871000	-2.115063000

12 (B3LYP, +30.4 kJ/mol)

8	-3.173310000	-0.968402000	-0.257570000
6	-2.023569000	-1.153881000	-0.035689000
8	-1.438911000	-2.119034000	0.682725000
6	-0.015556000	-1.956830000	0.695196000
6	0.212283000	-0.723628000	-0.120901000
6	1.574015000	-0.178676000	-0.496624000
8	2.482236000	-0.729964000	0.292925000
6	3.878239000	-0.354083000	0.057282000
8	1.746019000	0.591172000	-1.411765000
7	-0.926690000	-0.284542000	-0.552514000
6	-1.265334000	0.962607000	-1.260384000
6	-1.499372000	2.098879000	-0.221899000
6	-0.276842000	2.417935000	0.586587000
6	-0.147511000	2.144759000	1.893143000
1	0.336826000	-1.841218000	1.725549000
1	0.458736000	-2.840532000	0.252882000
1	3.986864000	0.723269000	0.188100000
1	4.162159000	-0.645631000	-0.954639000
1	4.443498000	-0.904923000	0.804752000
1	-0.446340000	1.192793000	-1.939474000
1	-2.183287000	0.756270000	-1.812594000
1	-2.344612000	1.833763000	0.421154000
1	-1.806411000	2.966707000	-0.818013000
1	0.540412000	2.907160000	0.058034000
1	-0.953550000	1.690317000	2.466323000
1	0.746129000	2.421158000	2.444742000

13 (B3LYP, +71.7 kJ/mol)

8	-3.298781000	0.106952000	-0.880514000
6	-2.384760000	-0.122554000	-0.145964000
8	-2.366437000	0.076059000	1.190357000
6	-1.079996000	-0.210507000	1.753430000
6	-0.143968000	-0.450981000	0.538508000
6	0.863799000	-1.604190000	0.800124000
6	1.804474000	-1.918487000	-0.336403000
6	3.120431000	-1.685089000	-0.306151000
7	-1.106058000	-0.638842000	-0.555655000
6	-0.579066000	-0.083026000	-1.751001000
8	0.361033000	1.029932000	-1.225989000
6	0.584626000	0.785244000	0.011399000
8	1.352553000	1.500033000	0.729780000
6	2.057838000	2.668292000	0.145393000
1	-1.151865000	-1.117695000	2.357727000
1	-0.774344000	0.627810000	2.382382000
1	1.431409000	-1.340201000	1.700411000
1	0.250641000	-2.480551000	1.039917000
1	1.370113000	-2.414646000	-1.203424000
1	3.762177000	-1.960072000	-1.137330000
1	3.606843000	-1.244582000	0.561759000
1	0.092727000	-0.732281000	-2.307876000
1	-1.335751000	0.396775000	-2.370176000
1	1.312668000	3.367944000	-0.232408000
1	2.714786000	2.310358000	-0.646622000
1	2.615804000	3.083017000	0.980127000

14 (B3LYP, +72.7 kJ/mol)

8	-3.104438000	0.015981000	-1.026921000
6	-2.130324000	-0.468461000	-0.529839000
7	-0.803316000	0.076965000	-0.626849000
6	-0.651960000	1.429351000	-1.003711000
6	-0.769140000	2.380924000	0.382264000
6	0.200935000	1.781488000	1.248291000
6	0.002753000	0.443776000	1.733554000
8	-2.069537000	-1.569131000	0.240572000
6	-0.708321000	-1.859395000	0.605633000
6	0.035130000	-0.537972000	0.335256000
6	1.506965000	-0.760772000	-0.061603000
8	2.120595000	0.385795000	-0.367164000
6	3.524064000	0.276813000	-0.776266000
8	2.007118000	-1.858537000	-0.067627000
1	0.314259000	1.632301000	-1.456564000
1	-1.483882000	1.736830000	-1.638839000
1	-1.810329000	2.243668000	0.684658000
1	-0.561635000	3.409399000	0.087286000
1	1.181355000	2.242038000	1.348614000
1	-1.004819000	0.256980000	2.114497000
1	0.767470000	0.076891000	2.418762000
1	-0.686867000	-2.180253000	1.646668000
1	-0.318034000	-2.653056000	-0.034829000
1	3.838461000	1.297475000	-0.982408000
1	4.104462000	-0.161901000	0.035928000
1	3.592267000	-0.346499000	-1.668187000

15 (B3LYP, +0.0 kJ/mol)

8	-2.959730000	-0.531045000	-1.213453000
6	-2.057174000	-0.609331000	-0.428615000
8	-1.913766000	-1.590514000	0.513619000
6	-0.893259000	-1.245281000	1.460044000
6	-0.114651000	-0.094118000	0.796279000
6	1.213642000	-0.435603000	0.126025000
8	1.554373000	-1.601698000	-0.246648000
6	2.797411000	-1.818308000	-1.025080000
8	1.931828000	0.608692000	-0.085947000
7	-0.972517000	0.270062000	-0.322957000
6	-1.057146000	1.663238000	-0.800422000
6	0.310504000	2.377542000	-0.667614000
6	1.163129000	1.826708000	0.474662000
6	0.333031000	1.157904000	1.573432000
1	-0.281022000	-2.127845000	1.651628000
1	-1.362522000	-0.913764000	2.391460000
1	3.641892000	-1.442589000	-0.448092000
1	2.841943000	-2.896014000	-1.154506000
1	2.697369000	-1.301184000	-1.979109000
1	-1.391997000	1.642820000	-1.838283000
1	-1.822637000	2.197286000	-0.224839000
1	0.889017000	2.322374000	-1.594479000
1	0.135770000	3.441180000	-0.476077000
1	1.957455000	2.495123000	0.799897000
1	-0.512031000	1.771190000	1.893151000
1	0.939816000	0.901015000	2.447114000
